# The basal forebrain to lateral habenula circuitry mediates social behavioral maladaptation

**DOI:** 10.1038/s41467-024-48378-y

**Published:** 2024-05-13

**Authors:** Jun Wang, Qian Yang, Xue Liu, Jie Li, Ya-Lan Wen, Yuzheng Hu, Tian-Le Xu, Shumin Duan, Han Xu

**Affiliations:** 1 Department of Neurobiology and Department of Psychiatry of the Second Affiliated Hospital, Zhejiang University School of Medicine, Hangzhou, 310058 China; 2 Nanhu Brain-computer Interface Institute, Hangzhou, 311100 China; 3 Liangzhu Laboratory, MOE Frontier Science Center for Brain Science and Brain-machine Integration, State Key Laboratory of Brain-machine Intelligence, Zhejiang University, 1369 West Wenyi Road, Hangzhou, 311121 China; 4 NHC and CAMS Key Laboratory of Medical Neurobiology, Zhejiang University, Hangzhou, 310058 China; 5 Lingang Laboratory, Shanghai, 200031 China; 6 Department of Psychology and Behavioral Sciences, Zhejiang University, Hangzhou, 310027 China; 7 Center for Brain Science and Department of Anatomy and Physiology, Shanghai Jiao Tong University School of Medicine, Shanghai, 200025 China

**Keywords:** Social behaviour, Neural circuits

## Abstract

Elucidating the neural basis of fear allows for more effective treatments for maladaptive fear often observed in psychiatric disorders. Although the basal forebrain (BF) has an essential role in fear learning, its function in fear expression and the underlying neuronal and circuit substrates are much less understood. Here we report that BF glutamatergic neurons are robustly activated by social stimulus following social fear conditioning in male mice. And cell-type-specific inhibition of those excitatory neurons largely reduces social fear expression. At the circuit level, BF glutamatergic neurons make functional contacts with the lateral habenula (LHb) neurons and these connections are potentiated in conditioned mice. Moreover, optogenetic inhibition of BF-LHb glutamatergic pathway significantly reduces social fear responses. These data unravel an important function of the BF in fear expression via its glutamatergic projection onto the LHb, and suggest that selective targeting BF-LHb excitatory circuitry could alleviate maladaptive fear in relevant disorders.

## Abstract (web summary)

Maladaptive fear is linked to many neuropsychiatric disorders, while its neural basis is not fully understood. Here, the authors show that the hyperactivity of the basal forebrain to lateral habenula glutamatergic circuit is crucial for social fear behavior.

## Introduction

Fear is typically an adaptive emotional response that is essential for the survival of animals including human beings. However, excessive and unnecessary fear to environmental stimuli represents a maladaptive state that has been implicated in many neuropsychiatric disorders, notably posttraumatic stress disorder (PTSD) and social anxiety disorder (SAD)^1–3^. Despite its prevalence and disabling consequences, the effective treatment options for maladaptive fear remain largely lacking. Therefore, a thorough understanding of the neural alterations underlying the emotional and behavioral maladaptation is currently necessary.

In the past decades, numerous studies have been conducted to unravel neural circuit mechanisms underlying learned fear acquired through conditioning in rodents. It is now well accepted that learned fear is encoded and regulated by widely distributed brain regions and corresponding neuronal circuitries^4^. Among which, the amygdala has been identified as a central brain structure responsible for the control of both fear learning and expression^5,6^. Besides, the medial prefrontal cortex (mPFC) is another brain region required for the retrieval of conditioned fear associated with sensory stimuli of various modalities^7,8^. The mPFC sends dense projections to both the basolateral amygdala (BLA) and the paraventricular nucleus of the thalamus (PVT). Interestingly, it was recently found that the mPFC to BLA projection is responsible for fear memory retrieval at early time points, whereas the mPFC to PVT projection is responsible for the maintenance of long-term fear memories^9^. This observation of a time-dependent shift in fear circuits reveals an extra complexity to the circuits underlying fear responses. Furthermore, the ventral hippocampus and the periaqueductal gray (PAG), among others have also been reported to participate in the expression of acquired fear memory^6,10,11^. Despite these mounting progresses, key brain regions and neural circuits underlying fear behavioral maladaptation are still not fully understood.

The basal forebrain (BF) is located in the rostroventral forebrain and is enriched with cholinergic projection neurons^12,13^. Recent animal studies have established a causal role of BF cholinergic neurons in synaptic plasticity and also behavioral learning including conditioned fear learning^14–17^. Interestingly, a human brain imaging study shows increased activity of the BF in PTSD patients during supraliminal processing of trauma-related words^18^. Similarly, abnormal activation of the BF structure in response to an angry face stimulus is recently reported in individuals with higher social anxiety^19^. These brain imaging studies suggest the engagement of the BF in the processing of negative emotions. However, whether and how the BF is directly involved in fear expression remains an important yet unresolved question.

Except for cholinergic neurons, the BF contains two other major neuronal subtypes, that is, glutamatergic neurons and GABAergic neurons^13,20^. Although previous studies have mainly focused on cholinergic neurons, the functions of BF glutamatergic neurons and GABAergic neurons start to be unraveled in recent years. For instance, the glutamatergic neurons and parvalbumin (PV)-expressing GABAergic neurons have been implicated in sleep-wake control^13^, and somatostatin (SST)-expressing GABAergic neurons play a role in hedonic feeding^21^ and also prosocial behavior^22^. However, the activities of the BF neuronal subtypes upon fear expression are not investigated, and their respective contribution to fear behavioral manifestation is not known either.

In the present study, we employed a social fear conditioning paradigm and induced robust social fear in mice^23–25^. We found that social fear behavior was associated with hyperactivity of BF glutamatergic neurons. Furthermore, the glutamatergic projection from the BF to the lateral habenula (LHb) was potentiated following social fear conditioning and was activated during social fear expression. More importantly, selective inhibition of the BF-LHb excitatory pathway substantially reduced social fear responses. Together, we report a previously unidentified function of the BF in the control of fear expression and social behavioral maladaptation.

## Results

### Social fear behavior is associated with BF hyperactivity

To induce social fear behavior, we subjected adult male mice to a social fear conditioning (SFC) paradigm^23–25^. During the SFC, an electric foot shock (1 s, 0.6 mA) was delivered each time as the experimental mouse investigated the stimulus mouse (Supplementary Fig. 1a). Consistent with previous studies, we found that mice experienced the SFC developed robust social fear and social avoidance when assessed with a three-chamber social interaction assay. In specific, conditioned mice spent much less time in both the social chamber and the social zone, and displayed an increased percentage of stretched postures during social approaches (Supplementary Fig. 1b–j). Besides, the SFC did not affect either locomotion or anxiety-like behavior as revealed with an open-field test (Supplementary Fig. 1k–n). Note that the social fear and avoidance behavior was observed when the conditioned mouse oriented toward an unfamiliar stimulus mouse, suggesting social behavioral adaptation to general social stimulus but not only to the specific mouse associated with conditioning. These observations confirmed that the SFC is able to induce behavioral alterations specifically in social domain.

**Fig. 1:**
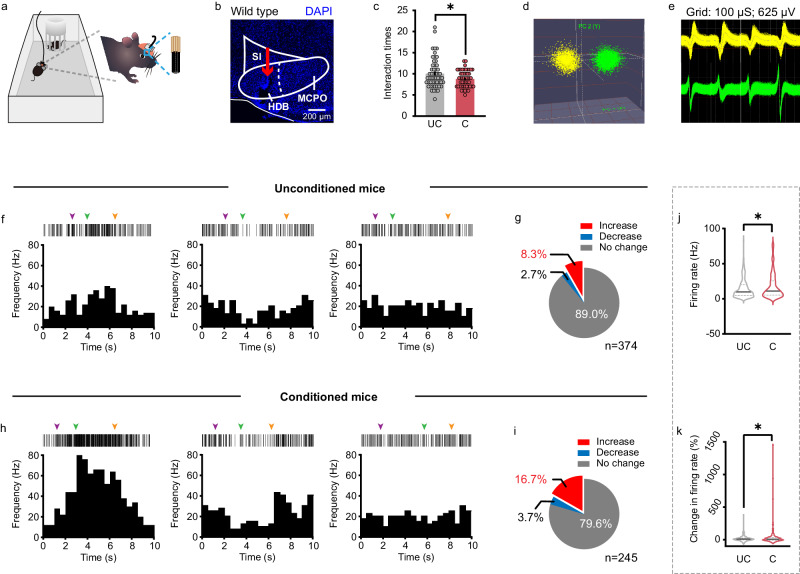
Social fear expression is associated with BF hyperactivity. **a** Schematic illustration of electrophysiological recording from a mouse subjected to a social approach-avoidance test. Enlargement shows multichannel tetrode implantation. **b** Example brain section showing the tract of tetrode implantation indicated by the red arrow. All mice (*n* = 23) have been checked independently with tips of tetrode in the BF.HDB, horizontal limb of the diagonal band of Broca; MCPO, magnocellular preoptic nucleus; SI, substantia innominate. **c** Social interaction times in unconditioned (*n* = 53 tests from 13 mice) and conditioned mice (*n* = 50 tests from 10 mice). Error bars indicate mean ± SEM. *P* = 0.0292, two-sided unpaired t-test. **d,** **e** Example extracellular waveform sorting. Two clusters of recorded neurons from a tetrode formed in three-dimensional space using a principal component analysis (**d**), and superimposed waveforms of two simultaneously recorded neurons from each cluster (**e**). **f** Raster plots (top) and peri-stimulus time histograms (PSTH; bottom) of example Increase (left), Decrease (middle) and No change (right) BF neurons during a social approaching epoch of an unconditioned mouse. The inverted arrows indicate the onset of approach (purple), the most proximity with the stimulus mouse (green), and the retreat from the stimulus mouse (orange), respectively. **g** Proportions of all recorded BF neurons from unconditioned mice with significantly Increase, Decrease or No change in firing rates during social interaction with a stimulus mouse. **h**, **i** The same as (**f**, **g**) but for conditioned mice. **j** The mean firing rate during social interaction of all recorded BF neurons in unconditioned (*n* = 374 neurons from 13 mice) and conditioned mice (*n* = 245 neurons from 10 mice). Solid lines indicate the median and dotted lines indicate the quartiles. *P* = 0.0113, two-sided unpaired *t*-test. **k** The percentage of change in firing rate [(social-baseline)/baseline × 100%] of all recorded BF neurons in both groups. Solid lines indicate the median and dotted lines indicate the quartiles. *P* = 0.0484, two-sided unpaired *t*-test. *P < 0.05. Source data are provided as a Source Data file.

To probe the real-time spiking activity of BF neurons during social fear, we performed multichannel electrophysiological recordings in freely behaving mice while they were engaged in a social approach-avoidance task in a single-chamber apparatus (20 × 40 × 20 cm), where a small cylinder-shaped acrylic cage was placed at middle of one side (Fig. 1a). Specifically, a total of 23 mice were implanted with microdrives containing eight adjustable tetrodes aimed at the BF. Of which, 10 mice were randomly selected to experience the SFC (C) and the remaining 13 mice without SFC experience were set as unconditioned controls (UC). The placement of tetrodes implantation in the BF was verified by *post hoc* microscopic inspection (Fig. 1b). To record more neurons, tetrodes were lowered by ~40 μm at the end of each daily recording session. During the test, only those social investigations exceeding 2 s were analyzed to accurately characterize neuronal activity. Note that the number of social investigations was reduced in conditioned mice (UC: 10.06 ± 0.48; C: 8.80 ± 0.28; P = 0.0292, unpaired t test) (Fig. 1c).

Spike activities were sorted into single-unit firing based on principal component analysis in a three-dimensional space (Fig. 1d, e). A total of 619 well-isolated neurons were analyzed, including 374 neurons from unconditioned mice and 245 neurons from conditioned mice. To avoid confounding effect of the difference in social interaction times between unconditioned and conditioned mice, we examined spiking activity of the first six interaction bouts for all tests. We found diverse firing patterns of BF neurons in both groups of mice. When compared with baseline activity, recorded neurons displayed Increase, Decrease, or No change in their firing rates during social interactions in both unconditioned mice (Fig. 1f) and conditioned mice (Fig. 1h). However, intriguingly, a notable fraction of BF neurons showed increased firing rates during social interaction in conditioned mice (41 out of 245, 16.7%), significantly more than the fraction in unconditioned mice (31 out of 374, 8.3%) (*P* = 0.0019, Fisher’s exact test) (Fig. 1g, i). Consistently, the average firing rate of BF neurons during social interaction was higher in conditioned mice as compared with that in unconditioned mice (UC: 14.72 ± 0.73 Hz, *n* = 374; C: 18.02 ± 1.16 Hz, *n* = 245; *P* = 0.0113, unpaired *t* test) (Fig. 1j). Similarly, the percentage of change in firing rate [(social-baseline)/baseline × 100%] of BF neurons was higher in conditioned mice as well (UC: 17.80 ± 2.42%; C: 31.84 ± 7.95%; *P* = 0.0484, unpaired *t*-test) (Fig. 1k). These findings demonstrated that a subset of BF neurons was intensely recruited during social fear expression.

### Inhibition of BF glutamatergic neurons reduces social fear

The BF contains three major genetically defined cell types: cholinergic neurons, glutamatergic neurons and GABAergic neurons^13,20^. In order to determine the activity of different cell types in social fear, we used fiber photometry to record Ca^2+^ signals from vGluT2, ChAT and vGAT neurons by expressing the Cre-dependent AAV-DIO-jGCaMP8s into the BF of vGluT2-Cre, ChAT-Cre or vGAT-Cre mice, respectively (Supplementary Fig. 2a, b). Following the SFC, fluorescence signals were monitored during social fear expression in a three-chamber social interaction task. We found that BF vGluT2 neurons were robustly activated during social interaction but not neutral cage investigation (Supplementary Fig. 2e, f). Both the peak and the area under curve (AUC) of the fluorescence signal were significantly higher during social interaction compared to neutral cage investigation (Supplementary Fig. 2g, h). In comparison, ChAT neurons exhibit only slight responses to both social interactions and neutral cage investigations (Supplementary Fig. 2i–l). Similarly, vGAT neurons exhibited mild responses to both social interactions and neutral cage investigations, and there was no significant difference between two conditions (Supplementary Fig. 2m–p). Moreover, mice that expressed EYFP did not show obvious fluorescence changes upon social or neutral cage interactions (Supplementary Fig. 2q–t), confirming that the signals observed in vGluT2-GCaMP mice were Ca^2+^ in nature but not motion artifacts. Together, these findings demonstrated a robust activation of BF vGluT2 neurons during social fear expression.

**Fig. 2:**
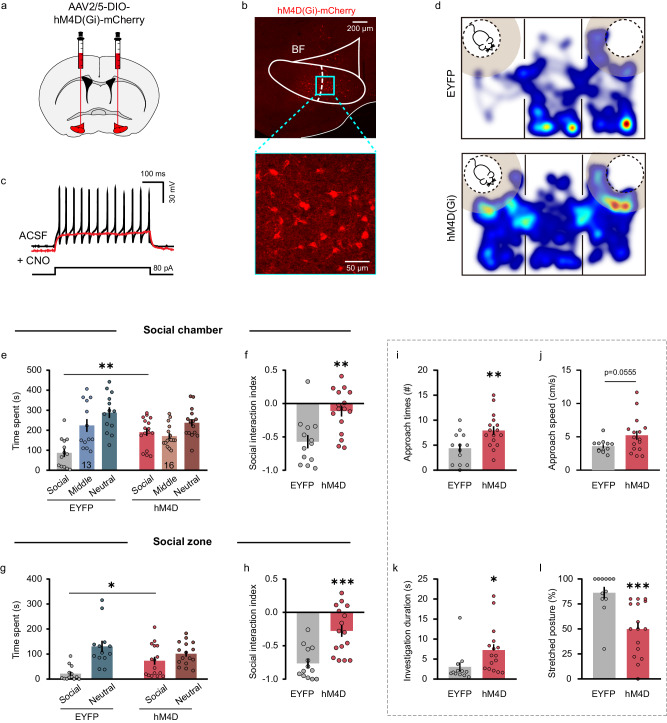
Chemogenetic inhibition of BF vGluT2 neurons reduces social fear. **a** Schematic illustration of hM4D(Gi)-mCherry virus injection in the bilateral BF of vGluT2-Cre mice. **b** Representative image showing the expression of hM4D(Gi)-mCherry in the BF. All mice (*n* = 16) have been checked independently with similar virus expression in the BF. **c** Example patch-clamp recording showing CNO application hyperpolarized an hM4D-expressing vGluT2 neuron and eliminated its firing (red line). **d** Representative heatmaps showing movement traces of an EYFP-expressing mouse (top) and an hM4D-expressing mouse (bottom) in three-chamber social interaction test. **e** Quantification of time spent by EYFP- and hM4D-expressing mice in each chamber. *n* = 13 mice for EYFP group; n = 16 mice for hM4D group. F_interaction_(2, 54) = 5.75, *P* = 0.0055; F_chamber_(2, 54) = 10.96, *P* = 0.0001; F_group_(1, 27) = 0.51, *P* = 0.4825; time spent in social chamber (EYFP vs. hM4D): *P* = 0.0033, two-way ANOVA followed by Bonferroni’s multiple comparisons. **f** Social interaction index [(time spent in the social chamber - time spent in the neutral chamber) / (time spent in the social chamber + time spent in the neutral chamber)] was increased in hM4D mice. *P* = 0.0013, two-sided unpaired *t*-test. **g** The same as (**e**) but for the 8 cm social zone. F_interaction_(1, 27) = 11.59, *P* = 0.0021; F_zone_(1, 27) = 33.53, *P* < 0.0001; F_group_(1, 27) = 0.38, *P* = 0.5436; time spent in social zone (EYFP vs. hM4D): *P* = 0.0436, two-way ANOVA followed by Bonferroni’s multiple comparisons. **h** The same as (**f**) but for the 8-cm social zone. *P* = 0.0002, two-sided unpaired *t* test. **i**–**l** Comparison of the approach times (**i**) (*P* = 0.0075), approach speed (**j**) (*P* = 0.0555), investigation duration (**k**) (*P* = 0.0404) and percentage of stretched postures (**l**) (*P* = 0.0006). n = 13 mice for EYFP group; n = 16 mice for hM4D group. Two-sided unpaired *t*-test. Error bars indicate mean ± SEM. *P < 0.05, **P < 0.01, ***P < 0.001. Source data are provided as a Source Data file.

To further determine the precise role of BF cell types in social fear, we selectively inhibited each of these three BF cell types and examined their influences on animals’ social behavioral outcomes. To do this, we first bilaterally injected AAV carrying Cre-dependent hM4D(Gi) or a control virus carrying EYFP into the BF of vGluT2-Cre mice (Fig. 2a, b). Patch-clamp recording in brain slices showed that application of CNO (10 μM) inhibited the current injection-induced spiking of BF vGluT2 neurons expressing hM4D-mCherry, indicating that the chemogenetic intervention could effectively inhibit those neurons (Fig. 2c). Four weeks after viral infusion, a three-chamber social interaction task was employed to assess the behavioral effect following inhibition of BF glutamatergic neurons in mice experienced SFC (Fig. 2d). We found that the time spent in the social chamber (EYFP: 87.12 ± 20.80 s, *n* = 13; hM4D: 190.70 ± 18.47 s, *n* = 16; *P* = 0.0033, two-way ANOVA followed by Bonferroni’s *post hoc* test) (Fig. 2e) and the social interaction index (EYFP, −0.57 ± 0.10; hM4D, −0.11 ± 0.08; *P* = 0.0013, unpaired *t*-test) (Fig. 2f) of hM4D-expressing mice were significantly increased as compared with those in the EYFP group. When 8 cm vicinity social zone was analyzed, increases in the social interaction time (EYFP, 21.96 ± 8.08 s; hM4D, 69.24 ± 16.30 s; *P* = 0.0436, two-way ANOVA followed by Bonferroni’s *post hoc* test) (Fig. 2g) and the social interaction index (EYFP, −0.76 ± 0.07; hM4D, −0.28 ± 0.09; *P* = 0.0002, unpaired *t*-test) (Fig. 2h) were also detected in hM4D-expressing group as compared with EYFP group. Besides, hM4D-expressing mice showed more approach times (EYFP: 4.39 ± 0.86; hM4D: 7.94 ± 0.86; *P* = 0.0075, unpaired *t*-test) (Fig. 2i), a marginally significant increase in approach speed (EYFP: 3.59 ± 0.28; hM4D: 5.24 ± 0.67; *P* = 0.0555, unpaired *t*-test) (Fig. 2j), an increase in the mean duration of individual investigation (EYFP: 3.04 ± 1.16; hM4D: 7.258 ± 1.446; *P* = 0.0404, unpaired *t*-test) (Fig. 2k), and a less percentage of stretched postures towards stimulus mice (EYFP: 86.22 ± 5.90; hM4D: 49.86 ± 6.71; *P* = 0.0006, unpaired *t*-test) (Fig. 2l). Intracardiac perfusion followed by microscopic inspection was performed on each mouse to verify virus expression in the BF, and those with virus expression confined to the BF were used for behavioral analysis (Supplementary Fig. 3a, b). In addition, open field test was performed to assess locomotion and anxiety-like behavior in the hM4D and EYFP-expressing mice following the SFC (Supplementary Fig. 3c). We found no difference in the total distance (Supplementary Fig. 3d), time in center (Supplementary Fig. 3e), or center entries (Supplementary Fig. 3f) between two groups, suggesting that inhibition of BF vGluT2 neurons had no side effect on animal’s locomotion or anxiety-like behavior. Together, these findings indicate that chemogenetic inhibition of BF vGluT2 neurons reduced social fear in mice following the SFC.

Similarly, we next examined the role of BF ChAT and vGAT neurons on social fear expression by chemogenetic inhibition of these neurons. In contrast to BF vGluT2 neurons, inhibition of ChAT or vGAT neurons had no significant effect on social fear behaviors (Supplementary Figs. 4 and 5). The BF ChAT hM4D-expressing mice showed similar time spent in both social chamber and social zone (Supplementary Fig. 4d–g), approach times (Supplementary Fig. 4h), approach speed (Supplementary Fig. 4i), the mean duration of individual investigations (Supplementary Fig. 4j), and a slightly increased percentage of stretched postures towards stimulus mice (Supplementary Fig. 4k). Moreover, chemogenetic inhibition of BF ChAT neurons reduced total distance moved in the open field test (Supplementary Fig. 4l, m), without altering time in center (Supplementary Fig. 4n) and center entries (Supplementary Fig. 4o). As a comparison, the BF vGAT hM4D-expressing mice showed similar behavioral performance in social fear expression test as compared with the EYFP-expressing control mice (Supplementary Fig. 5a–k), but markedly reduced total distance moved (Supplementary Fig. 5l, m), time in center (Supplementary Fig. 5n) and center entries (Supplementary Fig. 5o) in the open field test. These observations suggest that although inhibition of BF ChAT or vGAT neurons affected locomotion or general anxiety-like behavior, these manipulations did not alter social fear expression in mice following the SFC.

To further confirm the effect of BF vGluT2 neurons inhibition on social fear behavior, we also employed an optogenetic approach. Cre-dependent GtACR1 or a control virus carrying EYFP was bilaterally injected into the BF of vGluT2-Cre mice (Supplementary Fig. 6a, b), and blue light stimuli ( ~ 5 mW, 10 ms pulses at 20 Hz) were applied for the entire duration of the three-chamber social interaction test. We found that optogenetic inhibition of BF vGluT2 neurons also effectively increased the social interaction time and interaction index both in social chamber and social zone (Supplementary Fig. 6c–g). The approach times, approach speed, and mean duration of individual social investigation were increased, and the percentage of stretched postures was decreased in GtACR1-expressing mice as compared to those in the control group (Supplementary Fig. 6h–k). Also, optogenetic inhibition of BF vGluT2 neurons did not alter locomotion or anxiety-like behavior as assessed with an open-field test (Supplementary Fig. 6l–o). To know whether the increase in social time after inhibiting BF vGluT2 neurons was due to an impact on animal sociability, we performed optogenetic inhibition in unconditioned naïve mice (Supplementary Fig. 7a, b). Three-chamber social interaction assay showed that either social interaction time or interaction index in the social chamber (Supplementary Fig. 7d, e) or social zone (Supplementary Fig. 7h, i) was not changed in GtACR1-expressing mice as compared to that in the EYFP group. Moreover, there was no difference in the approach times and mean duration of individual social investigation to the social chamber (Supplementary Fig. 7f, g) or social zone (Supplementary Fig. 7j, k) between GtACR1-expressing mice and EYFP-expressing mice. These data suggest that optogenetic inhibition of BF vGluT2 neurons did not affect animal’s sociability under our experimental conditions. Taken together, both chemogenetic and optogenetic studies demonstrate that BF vGluT2 neurons are necessary for the regulation of social fear behavior induced by SFC.

To further examine the role of BF vGluT2 on social fear under more naturalistic conditions, we also employed a sub-chronic social defeat paradigm to induce social fear^26^. We found that BF vGluT2 neurons were robustly activated during social interactions with a CD1 stimulus mouse compared to neutral cage investigations (Supplementary Fig. 8a–d). Moreover, chemogenetic inhibition of BF vGluT2 neurons reduced social fear expression induced by social defeat (Supplementary Fig. 8e–h). Interesting, we found that BF vGluT2 neurons were also responsive to shock-paired odor following a classical CS (chocolate odor)-US (foot shock) association (Supplementary Fig. 9a–d), and inhibition of these neurons suppressed odor elicited fear expression (Supplementary Fig. 9e–k). These observations indicate that BF vGluT2 neurons have a general role in both socially relevant fear behaviors and non-social fear learning.

### BF glutamatergic neurons are activated by social fear expression

**Fig. 3:**
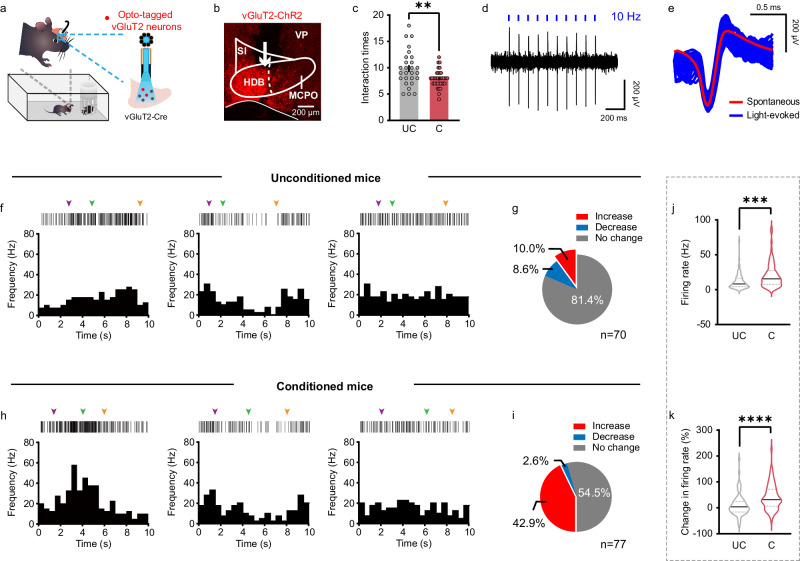
Social fear expression activates BF glutamatergic neurons. **a** Schematic illustration of electrophysiological recording of opto-tagged BF vGluT2 neurons. **b** Example brain section showing the tract of optrode implantation indicated by the white arrow. VP, ventral pallidum. All mice (*n* = 17) have been checked independently with tips of optrode in the BF. **c** Social interaction times in unconditioned (*n* = 27 tests from 8 mice) and conditioned mice (*n* = 36 tests from 9 mice). Error bars indicate mean ± SEM. *P* = 0.0034, two-sided unpaired t-test. **d** Example trace of light-evoked spikes from an opto-tagged vGluT2 neuron in the BF. Blue ticks, light pulses at 10 Hz. **e** Overlay of light-evoked (blue) and averaged spontaneous (red) spike waveforms from the example unit. **f** Raster plots (top) and PSTH (bottom) of example Increase (left), Decrease (middle) and No change (right) BF vGluT2 neurons during a social approaching epoch of an unconditioned mouse. The inverted arrows indicate the onset of approach (purple), the most proximity with the stimulus mouse (green), and the retreat from the stimulus mouse (orange), respectively. **g** Proportions of all opto-tagged BF vGluT2 neurons from unconditioned mice with significantly Increase, Decrease or No change in firing rates during social interaction with a stimulus mouse. **h**, **i** The same as (**f**, **g**) but for conditioned mice. **j** The mean firing rate during social investigation of all opto-tagged BF vGluT2 neurons in unconditioned (*n* = 70 neurons from 8 mice) and conditioned mice (*n* = 77 neurons from 9 mice). Solid lines indicate the median and dotted lines indicate the quartiles. *P* = 0.0006, two-sided unpaired *t*-test. **k** The percentage of change in firing rate [(social-baseline)/baseline × 100%] of all opto-tagged BF vGluT2 neurons in both groups. Solid lines indicate the median and dotted lines indicate the quartiles. *P* < 0.0001, two-sided unpaired *t*-test. **P < 0.01, ***P < 0.001, ****P < 0.0001. Source data are provided as a Source Data file.

The aforementioned fiber photometry registered the bulk activity of BF vGluT2 neurons. To further examine the real-time spiking activity of individual BF vGluT2 neurons during social fear expression, in vivo electrophysiological recordings combined with optogenetic tagging were conducted in free-moving mice. We virally expressed ChR2 in BF vGluT2 neurons and then implanted an optrode consisting of one optic fiber surrounded by 8 tetrodes (Fig. 3a). A total of 17 mice implanted with optrode were divided into conditioned (*n* = 9) and unconditioned (*n* = 8) groups. The placement of optrode in the BF was verified by *post hoc* microscopic inspection (Fig. 3b). Blue light stimuli (470 nm, 1 to 2 ms, 0.1–1.0 mW) at 10 or 20 Hz were applied at the end of each recording session, and single units exhibiting reliable light-evoked spikes with short latencies were identified as vGluT2 neurons. A total of 147 vGluT2 neurons were identified, including 70 neurons from unconditioned mice and 77 from conditioned mice. Representative responses and waveforms to the light stimuli are shown in Fig. 3d, e, respectively. The number of social interactions was reduced in conditioned mice (UC: 9.85 ± 0.65; C: 7.89 ± 0.27; P = 0.0034, unpaired *t*-test) (Fig. 3c). We examined spiking activity of the first six social interaction bouts, and found diverse firing patterns of BF vGluT2 neurons in both groups of mice. When compared with baseline activity, vGluT2 neurons displayed Increase, Decrease, or No change in their firing rates during social interactions in both unconditioned mice (Fig. 3f, g) and conditioned mice (Fig. 3h, i). Interestingly, we found that a majority of the BF vGluT2 neurons showed increased firing rates during social interaction in conditioned mice (33 out of 77, 42.9%), significantly more than the fraction in unconditioned mice (7 out of 70, 10.0%) (*P* < 0.0001, Fisher’s exact test) (Fig. 3g, i). Consequently, the average firing rate of BF vGluT2 neurons during social interaction was higher in conditioned mice as compared with that in unconditioned mice (UC: 11.73 ± 1.37 Hz, *n* = 70; C: 20.55 ± 2.04 Hz, *n* = 77; *P* = 0.0006, unpaired *t*-test) (Fig. 3j). Besides, the percentage of change in firing rate was higher in conditioned mice as well (UC: 11.43 ± 5.15%; C: 42.33 ± 5.66%; *P* < 0.0001, unpaired *t*-test) (Fig. 3k). These observations reveal a close association between the hyperactivity of BF glutamatergic neurons and social fear behavior.

### BF glutamatergic neurons innervate the LHb and the VTA

**Fig. 4:**
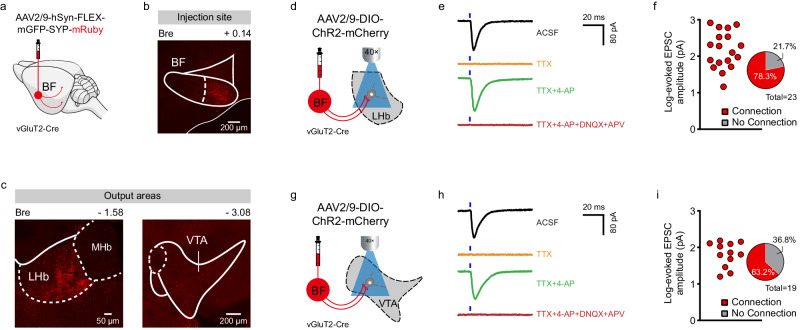
BF glutamatergic neurons project to the LHb and VTA. **a** Schematic illustration of AAV2/9-hSyn-GFP-Synaptophysin-mRuby injection in the unilateral BF of vGluT2-Cre mice. **b** A representative image showing SYP-mRuby expression in the BF. All mice (*n* = 4) have been checked independently with similar virus expression in the BF. **c** Representative images showing the distribution of mRuby labeled axons in the LHb (left) and VTA (right). MHb, medial habenular nucleus. All mice (*n* = 4) have been checked independently with similar virus expression. **d** Schematic illustration of optogenetic activation of axon terminals of BF vGluT2 neurons and patch-clamp recording in LHb neurons. **e** Example traces of postsynaptic responses of BF vGluT2 to LHb projections induced by optical stimuli (as indicated by blue ticks) under bath of artificial cerebrospinal fluid (ACSF; black line), and in addition of tetrodotoxin (TTX, 1 µM; orange line), TTX + 4-aminopyridine (4-AP, 100 µM; green line), or TTX + 4-AP + DNQX (10 µM) + AP-5 (20 µM) (red line). DNQX, 6,7-Dinitroquinoxaline-2,3 (1H,4H)-dione; AP-5, DL-2-Amino-5-phosphonopentanoic acid. **f** Statistics of the amplitude of postsynaptic currents and proportion of responsive neurons recorded from the LHb neurons (*n* = 23 neurons from 2 mice). **g**–**i** The same as (**d**–**f**) but for BF glutamatergic to VTA projections (*n* = 19 neurons from 4 mice). Source data are provided as a Source Data file.

Among many downstream targets of BF glutamatergic neurons^20^, the LHb and the ventral tegmental area (VTA) represent the two major regions that are closely linked to emotional processing^27,28^. To explore the neural circuitry mechanism underlying social fear expression by BF glutamatergic neurons, we therefore mainly focused on their projections to these two brain regions. To verify structural connectivity between BF glutamatergic neurons and the LHb/VTA, we first used anterograde synaptic tracer AAV2/9-hSyn-GFP-Synaptophysin (SYP)-mRuby to label their axon terminals using vGluT2-Cre mice (Fig. 4a, b). At 6 weeks after unilateral virus injection in the BF, mice were euthanized and 40 µm frozen brain sections containing the LHb or the VTA were collected. We found that dense SYP: mRuby puncta were expressed across the entire LHb and VTA (Fig. 4c), which is consistent with previous findings^20,29^.

We next characterized functional connectivity between BF glutamatergic neurons and the LHb with patch-clamp recording in brain slice preparations. Briefly, we stimulated axon terminals expressing ChR2 of presynaptic BF vGluT2 neurons with blue light, and simultaneously recorded from LHb neurons (Fig. 4d). Typically, a brief blue light stimulation elicited an inward current in LHb neurons, which was totally blocked by bath application of a sodium channel blocker tetrodotoxin (TTX) (Fig. 4e). Addition of potassium channel blocker 4-aminopyridine (4-AP) augmented the postsynaptic current that was completely eliminated by glutamatergic receptor antagonists DNQX and AP-5 (Fig. 4e). These observations suggest that BF glutamatergic neurons make monosynaptic excitatory connections with LHb neurons. Although the amplitude of evoked excitatory postsynaptic currents (EPSCs) varied, a large majority of all recorded neurons (18 out of 23 neurons from 2 mice, 78.3%) showed clear responses (Fig. 4f). To dissect functional connectivity between BF vGluT2 and the VTA neurons, brain slice recording was also performed in this projection pathway (Fig. 4g). As a comparison, BF glutamatergic neurons also made monosynaptic excitatory connections with VTA neurons (Fig. 4h), and 63.2% of all recorded VTA neurons (12 out of 19 neurons from 4 mice) responded to photostimuli (Fig. 4i). Together, BF glutamatergic neurons are frequently connected with both LHb and VTA neurons and are therefore able to strongly innervate the LHb and the VTA.

### Social fear selectively activates BF-LHb glutamatergic pathway

**Fig. 5:**
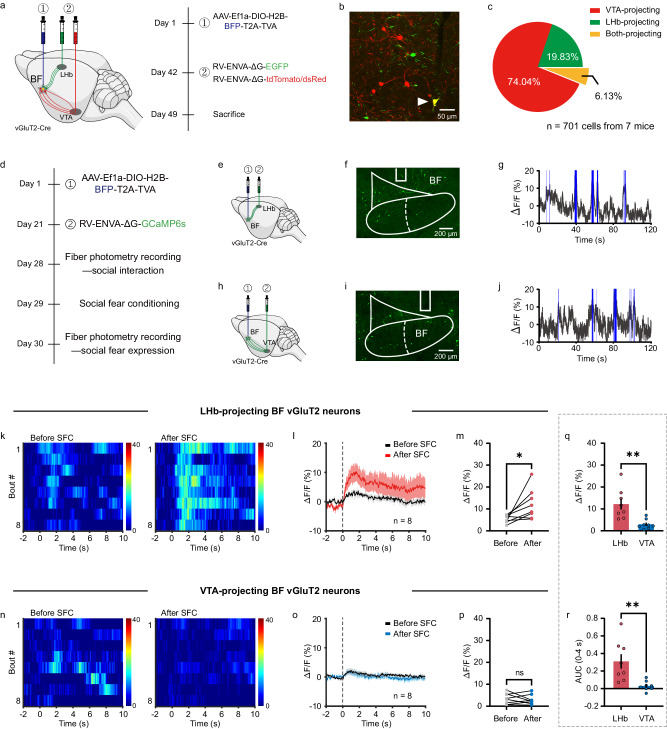
Social fear expression selectively activates LHb-projecting BF glutamatergic neurons. **a** Schematic illustration and timeline of rabies virus (RV)-based tracing of LHb-projecting and VTA-projecting BF vGluT2 neurons. **b** Representative image showing the LHb-projecting (green) and VTA-projecting (red) vGluT2 neurons in the BF. All mice (*n* = 7) have been checked independently with similar virus expression in the BF. **c** Proportion of BF vGluT2 neurons that project to the LHb (green), VTA (red) or both (yellow). **d** Timeline of RV-GCaMP6s injection and fiber photometry recording in a three-chamber social interaction test before and after the SFC. **e**, **f** Schematic illustration (**e**) and representative image (**f**) of RV-GCaMP6s expression in LHb-projecting BF vGluT2 neurons. All mice (*n* = 8) have been checked independently with similar virus expression in the BF. **g** A representative trace of spontaneous fluorescence signals recorded in LHb-projecting BF vGluT2 neurons. Blue lines indicate periods with spontaneous calcium events. **h**–**j** The same as (**e**–**g**) but for VTA-projecting BF vGluT2 neurons. All mice (*n* = 8) have been checked independently with similar virus expression in the BF. **k** Heatmap of Ca^2+^ signals of a mouse with GCaMP6s expression in LHb-projecting BF vGluT2 neurons. Each row represents one bout, and the color scale at the right indicates ΔF/F. **l** The peri-event plot of the mean Ca^2+^ transient during social interactions before or after the SFC (*n* = 8 mice). The thick line indicates the mean, and the shaded area indicates SEM. **m** Statistical comparison of peak fluorescence signals before and after SFC. *n* = 8 mice. *P* = 0.0282, two-sided paired *t*-test. **n**–**p** The same as (**k**–**m**) but for VTA-projecting BF vGluT2 neurons (*n* = 8 mice). **p** *P* = 0.5875, two-sided paired *t*-test. **q**, **r** Statistical comparison of peak (**q**) (*P* = 0.0030) and area under curve (**r**) (*P* = 0.0052) of fluorescence signals. *n* = 8 mice in each group, two-sided unpaired *t*-test. Error bars indicate mean ± SEM. ns, no significant difference, *P < 0.05, **P < 0.01. Source data are provided as a Source Data file.

To further explore the downstream circuit mechanism of BF vGluT2 neurons underlying social fear expression, we first examined the BF vGluT2 neurons projecting to either LHb or VTA with a retrograde neuronal tracing strategy using rabies virus (RV). In brief, a Cre-dependent virus encoding avian tumor virus receptor A (TVA) was injected into the BF of vGluT2-Cre mice, and six weeks later RV-EnvA-ΔG-DsRed/tdTomato or RV-EnvA-ΔG-EGFP was injected into ipsilateral VTA and LHb, respectively (Fig. 5a). After waiting for an additional week, mice were euthanized and BF vGluT2 neurons monosynaptically projecting to these two downstream regions were examined (Fig. 5b). Among all retrogradely labelled BF vGluT2 neurons, we observed that 74.04% cells (519 out of 701) projected to VTA only, 19.83% cells (139 out of 701) projected to LHb only, and a tiny fraction of cells (6.13%, 43 out of 701) projected to both VTA and LHb (Fig. 5c).

To determine the involvement of BF-LHb and BF-VTA glutamatergic pathway in social fear behavior, we then measured the activity of LHb- and VTA-projecting BF vGluT2 neurons with fiber photometry. AAV-EF1a-DIO-H2B-BFP-T2A-TVA was injected into the BF of vGluT2-Cre mice, and three weeks later RV-EnvA-ΔG-GCaMP6s was injected into VTA or LHb, and an optical fiber was implanted into the BF (Supplementary Fig. 10a, b, e, f). Nissl staining revealed that the number of neurons in the recording side was similar to the contralateral side (Supplementary Fig. 10c, d, g, h), suggesting no obvious cell loss following RV expression. During social fear expression in a three-chamber social interaction task, we found that the LHb-projecting BF vGluT2 neurons were robustly activated when the conditioned mice interacting with a stimulus mouse as compared with interacting with the opposite empty neutral cage (Supplementary Fig. 10i–k). In contrast, the VTA-projecting BF vGluT2 neurons were not responsive during either social interaction or neutral interaction (Supplementary Fig. 10l–n). Both the peak and AUC of the fluorescence signals were significantly higher in LHb-projecting cells during social fear compared to VTA-projecting BF vGluT2 neurons (Supplementary Fig. 10o, p).

To investigate dynamic changes in the activity of BF vGluT2 neurons associated with SFC, we performed fiber photometry recordings before and after conditioning (Fig. 5d). Representative RV expressions in LHb-projecting and VTA-projecting BF vGluT2 neurons are shown in Fig. 5f, i, respectively. Note that only recordings with spontaneous calcium events during the first 10 min habituation session were included for further analysis (Fig. 5g, j). We observed a slight activation in LHb-projecting vGluT2 neurons during interaction with a stimulus mouse before SFC, whereas an obviously larger activation was detected after SFC (Fig. 5k, l). Specifically, the peak of Ca^2+^ transients was significantly higher during social fear expression after the SFC than that before the SFC (Social fear: 12.20 ± 2.45%; Social: 5.39 ± 0.61%; *n* = 8, *P* = 0.0282, paired *t*-test) (Fig. 5m). In contrast, the VTA-projecting BF vGluT2 neurons had a slight activity during social interaction both before and after the SFC (Social fear: 2.98 ± 0.78%; Social: 3.48 ± 0.87%; *n* = 8, *P* = 0.5875, paired *t*-test) (Fig. 5n–p). When comparing the Ca^2+^ transients during social fear, we found that both the peak (LHb: 12.20 ± 2.45%; VTA: 2.98 ± 0.78%; *P* = 0.0030, unpaired *t*-test) and the AUC (LHb: 0.31 ± 0.08; VTA: 0.03 ± 0.02; *P* = 0.0052, unpaired *t*-test) were significantly larger in LHb-projecting BF vGluT2 neurons (Fig. 5q, r). These data indicated that social fear expression selectively activated LHb-projecting rather than VTA-projecting BF glutamatergic neurons.

Next, to further examine the direct involvement of BF-LHb or BF-VTA excitatory pathway during social fear, we also measured the activity of axon terminals of BF vGluT2 neurons in the LHb or VTA in another set of experiments. To achieve this, AAV2/9-hSyn-DIO-jGCaMP8s was injected to the BF of vGluT2-Cre mice, and four weeks later an optical fiber was implanted into the LHb or VTA for fiber photometry to record the activity of their axon terminals (Supplementary Fig. 11a–f). We found that in comparison with neutral interaction, social interaction significantly activated BF vGluT2 neuronal terminals in the LHb (Supplementary Fig. 11g–i) but not in the VTA (Supplementary Fig. 11j–l). In addition, both the peak and the AUC of Ca^2+^ transients during social interaction recorded in the LHb were higher than those in the VTA (Supplementary Fig. 11m, n). Together, these data corroborate findings from recording glutamatergic cell bodies (Fig. 5) and uncover that social fear expression selectively activated BF-LHb but not BF-VTA glutamatergic pathway.

### Inhibition of BF-LHb glutamatergic pathway alleviates social fear

**Fig. 6:**
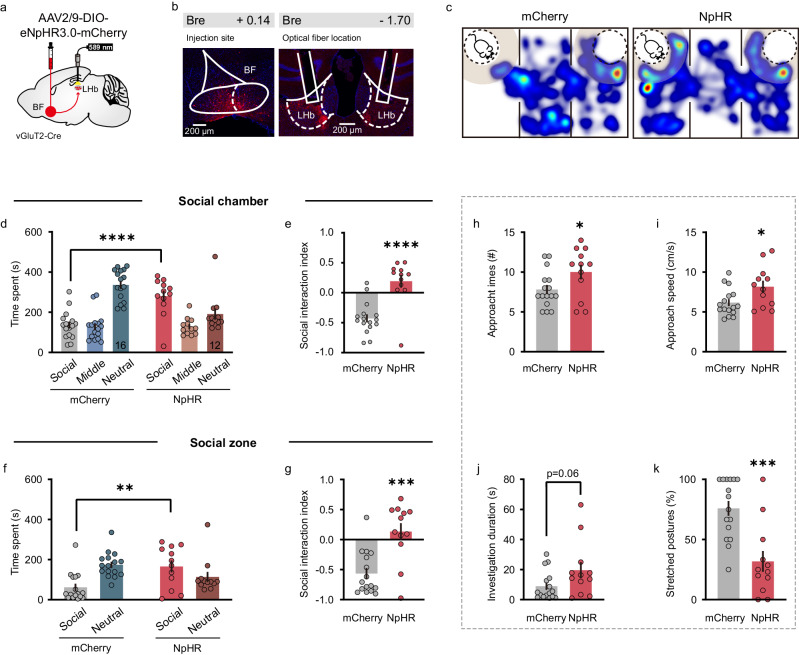
Optogenetic inhibition of BF-LHb glutamatergic pathway alleviates social fear. **a** Schematic illustration of eNpHR3.0-mCherry virus injection in the BF of vGluT2-Cre mice and optical fibers implantation in the LHb. **b** Representative images showing NpHR expression in the BF, and placement of optical fibers in the LHb with NpHR-expressing axon terminals. All mice (*n* = 12) have been checked independently with similar virus expression. **c** Heatmaps showing the movement traces of an mCherry-expressing control mouse (left) and an NpHR-expressing mouse (right) in a three-chamber social interaction test. **d** Quantification of time spent by mCherry- and NpHR-expressing mice in each chamber. *n* = 16 for mCherry group; *n* = 12 for NpHR group. F_interaction_(2, 52) = 16.48, *P* < 0.0001; F_chamber_(2, 52) = 14.61, *P* < 0.0001; F_group_(1, 26) = 0.53, *P* = 0.4730; time spent in social chamber (mCherry vs. NpHR): *P* < 0.0001, two-way ANOVA followed by Bonferroni’s multiple comparisons. **e** Social interaction index was significantly increased in NpHR mice. *n* = 16 mice for mCherry group; *n* = 12 mice for NpHR group. *P* < 0.0001, two-sided unpaired *t*-test. **f** The same as (**d**) but for the 8 cm social zone. F_interaction_(1, 26) = 11.37, *P* = 0.0023; F_zone_(1, 26) = 1.52, *P* = 0.2294; F_group_(1, 26) = 1.38, *P* = 0.2506; time spent in social zone (mCherry vs. NpHR): *P* = 0.0026, two-way ANOVA followed by Bonferroni’s multiple comparisons. **g** The same as (**e**) but for the 8 cm social zone. *n* = 16 mice for mCherry group; *n* = 12 mice for NpHR group. *P* = 0.0002, two-sided unpaired *t*-test. **h**–**k** Quantification of the approach times (**h**) (*P* = 0.0420), approach speed (**i**) (*P* = 0.0207), investigate duration (**j**) (*P* = 0.0595) and percentage of stretched postures (**k**) (*P* = 0.0002). *n* = 16 mice for mCherry group; *n* = 12 mice for NpHR group. Two-sided unpaired *t*-test. Error bars indicate mean ± SEM. *P < 0.05, **P < 0.01, ***P < 0.001, ****P < 0.0001. Source data are provided as a Source Data file.

To test whether BF-LHb glutamatergic projection is necessary for social fear expression, we inhibited this pathway by expressing Cre-dependent halorhodopsin (eNpHR3.0) in BF vGluT2 neurons and implanting optical fibers bilaterally in the LHb (Fig. 6a, b). Constant yellow light ( ~ 5 mW) was delivered during the entire three-chamber social interaction test in both NpHR- and mCherry-expressing mice. Representative heatmaps of the movement traces of an mCherry-expressing mouse and an NpHR-expressing mouse are shown in Fig. 6c. As compared to the control mice expressing mCherry, those expressing NpHR exhibited significant increases in the time spent in the social chamber (mCherry: 136.15 ± 17.40 s, *n* = 16; NpHR: 280.25 ± 27.39 s, *n* = 12; *P* < 0.0001, two-way ANOVA followed by Bonferroni’s *post hoc* test) (Fig. 6d), and the social interaction index (mCherry: −0.43 ± 0.07; NpHR: 0.19 ± 0.11; *P* < 0.0001, unpaired *t* test) (Fig. 6e). When social zone was analyzed, increases in the social interaction time (mCherry: 61.83 ± 17.85 s; NpHR: 165.91 ± 29.64 s; *P* = 0.0026, two-way ANOVA followed by Bonferroni’s *post hoc* test) (Fig. 6f) and the social interaction index (mCherry: −0.57 ± 0.09; NpHR: 0.13 ± 0.14; *P* = 0.0002, unpaired *t* test) (Fig. 6g) were also detected in NpHR-expressing group. Besides, NpHR-expressing mice showed more approach times (mCherry: 7.81 ± 0.57; NpHR: 10.00 ± 0.90; *P* = 0.0420, unpaired *t*-test) (Fig. 6h), faster approach speed (mCherry: 6.17 ± 0.43 cm/s; NpHR: 8.17 ± 0.74 cm/s; *P* = 0.0207, unpaired *t*-test) (Fig. 6i), a marginally significant increase in mean investigation duration (mCherry: 8.93 ± 2.40; NpHR: 19.54 ± 5.35; *P* = 0.0595, unpaired *t*-test) (Fig. 6j), and a reduction in the percentage of stretched postures (mCherry: 75.9 ± 6.18%; NpHR: 31.74 ± 8.67%; *P* = 0.0002, unpaired *t*-test) (Fig. 6k) as compared with mCherry group. Viral expression and placement of optical fiber were post-mortem checked, and only those with virus expression confined to the BF and fiber implanted in the LHb were analyzed for behavioral analysis (Supplementary Fig. 12a, c). Photostimulation (589 nm, 250 ms) efficiently hyperpolarized and suppressed their firing activities evoked by depolarizing current injection in an NpHR-expressing vGluT2 neuron (Supplementary Fig. 12b). In addition, an open field test was also performed to assess locomotion and anxiety-like behavior in NpHR and mCherry-expressing mice following the SFC (Supplementary Fig. 12d). We found that the total movement distance (Supplementary Fig. 12e), time in center (Supplementary Fig. 12f), as well as the center entries (Supplementary Fig. 12g) were similar between NpHR-expressing mice and mCherry-expressing mice. Furthermore, to test whether the increase in social time after inhibiting BF-LHb glutamatergic projection was due to an impact on animal’s sociability, we performed optogenetic inhibition in unconditioned naïve mice (Supplementary Fig. 13a, b). Three-chamber social interaction assay showed that either social interaction time or interaction index in the social chamber (Supplementary Fig. 13d, e) or social zone (Supplementary Fig. 13h, i) was not changed in NpHR-expressing mice as compared to that in the mCherry group. Moreover, there was no difference in the entry times and mean duration of individual social investigation to the social chamber (Supplementary Fig. 13f, g) or social zone (Supplementary Fig. 13j, k) between NpHR-expressing mice and mCherry-expressing mice. Together, these results demonstrated that selective inhibition of BF-LHb glutamatergic projection ameliorated social fear without changing sociability in mice.

As a comparison, we also examined the potential contribution of BF-VTA glutamatergic projection in social fear behavior. The same optogenetic manipulation was employed to inhibit BF-VTA glutamatergic projection and animals’ social fear was assessed with a three-chamber social interaction assay (Supplementary Fig. 14a–c). We found that optogenetic inhibition of BF-VTA glutamatergic pathway did not alter social interaction time and interaction index either in social chamber or in social zone (Supplementary Fig. 14d–g). The approach times, approach speed, and mean duration of individual social investigation as well as percentage of stretched postures were similar between NpHR-expressing mice and mCherry-expressing control mice (Supplementary Fig. 14h–k). These results echo the finding that social fear did not activate BF-VTA glutamatergic pathway (Fig. 5 and Supplementary Figs. 10 and 11), and suggest that BF-VTA glutamatergic pathway was not required for social fear expression.

The above loss-of-function studies suggest an essential role of the BF-LHb glutamatergic circuit in regulating social fear expression. To further establish functional role of this circuit in social fear, we then optogenetically activated this pathway in social fear conditioned mice. In specific, we expressed Cre-dependent channelrhodopsin (ChR2) in BF vGluT2 neurons and implanted optical fibers bilaterally in the LHb (Supplementary Fig. 15a, b). Photostimuli (10 ms pulses at 20 Hz) were delivered during the entire three-chamber social interaction test in both ChR2- and mCherry-expressing mice. We found that optogenetic activation of BF-LHb glutamatergic pathway did not alter social interaction time and interaction index either in social chamber or in social zone (Supplementary Fig. 15d–g). The approach times, approach speed, and mean duration of individual social investigation as well as percentage of stretched postures were similar between ChR2-expressing mice and mCherry-expressing control mice (Supplementary Fig. 15h–k). This observation is likely due to a ceiling effect such that extra activation of BF-LHb glutamatergic projection did not further exacerbate animals’ fear behavioral responses.

### SFC potentiates BF-LHb glutamatergic synaptic strength

**Fig. 7:**
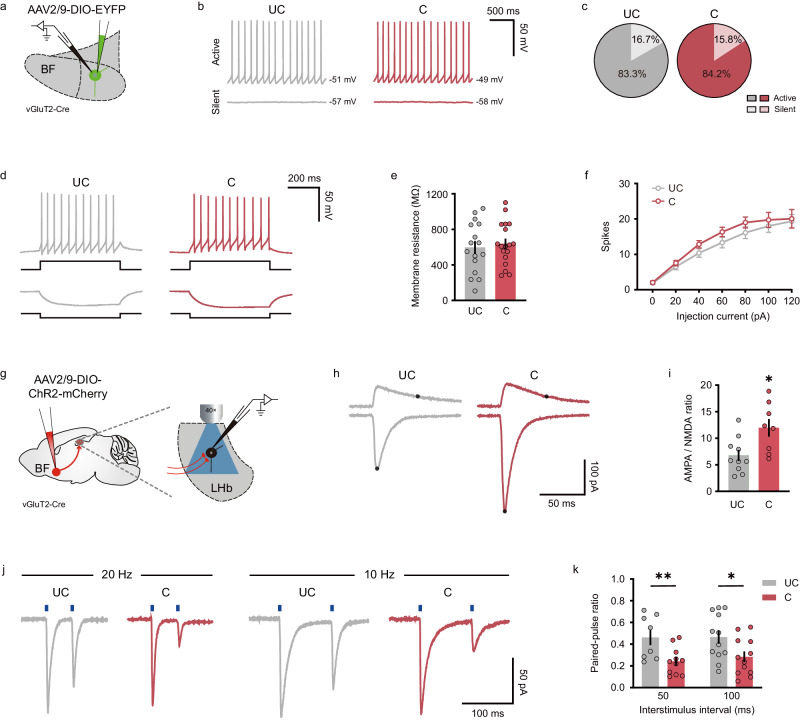
SFC potentiates BF-LHb glutamatergic functional connection. **a** Schematic illustration of brain slice recordings in BF vGluT2 neurons. **b** Representative membrane potential traces of active (top) and silent (bottom) neurons from unconditioned (left) and conditioned (right) mice. The numerical voltage at the right of each trace indicates resting membrane potential. **c** Proportion of active and silent neurons. Unconditioned, *n* = 18 neurons from 3 mice; conditioned, *n* = 19 neurons from 4 mice. **d** Representative traces of positive (60 pA, top) and negative (−30 pA, bottom) current injections in BF vGluT2 neurons from an unconditioned (left) and a conditioned (right) mouse. **e** Comparison of membrane resistance between unconditioned and conditioned groups. Unconditioned, *n* = 16 neurons from 3 mice; conditioned, *n* = 18 neurons from 4 mice. *P* = 0.9973, two-sided unpaired *t*-test. **f** The number of action potentials in response to various current injections. Unconditioned, *n* = 18 neurons from 3 mice; conditioned, *n* = 19 neurons from 4 mice. **g** Schematic illustration of optogenetic activation of axon terminals of BF vGluT2 neurons and patch-clamp recording in LHb neurons. **h** Representative traces of postsynaptic AMPA (bottom) and NMDA (top) currents from an unconditioned (left) and a conditioned (right) mouse. The black dots on traces indicate the amplitudes of AMPA and NMDA currents. **i** Quantification of AMPA/NMDA ratio in both groups. Unconditioned, *n* = 10 neurons from 4 mice; conditioned, *n* = 8 neurons from 4 mice. *P* = 0.0138, two-sided unpaired *t*-test. **j** Representative traces of postsynaptic currents from an unconditioned (gray) and a conditioned mouse (red) elicited by 20 Hz (left) and 10 Hz (right) paired-pulse light stimuli. **k** Comparison of paired-pulse ratio (PPR) of postsynaptic currents between two groups. 50 ms: unconditioned, *n* = 8 neurons from 3 mice; conditioned, *n* = 11 neurons from 3 mice; *P* = 0.0092. 100 ms: unconditioned, *n* = 12 neurons from 3 mice; conditioned, *n* = 12 neurons from 4 mice; *P* = 0.0300, two-sided unpaired *t* test. Error bars indicate mean ± SEM. *P < 0.05, **P < 0.01. Source data are provided as a Source Data file.

Social behavioral adaption and maladaptation in response to environmental challenges are usually accompanied by cellular and synaptic plasticity^30,31^. Given that inhibition of BF-LHb glutamatergic projection did not alter sociability in naïve mice (Supplementary Fig. 13), but markedly reduced social fear expression in mice following the SFC (Fig. 6), we reasoned that the SFC experience could affect the intrinsic excitability of BF vGluT2 neurons and/or synaptic connectivity between BF vGluT2 neurons and the LHb. To address this question, we first performed patch-clamp recordings from BF vGluT2 neurons and examined their intrinsic excitability (Fig. 7a). We found that in both conditioned and unconditioned mice, BF glutamatergic neurons can be divided into two distinct categories: active neurons firing spontaneous action potentials and silent neurons displaying no spontaneous action potentials (Fig. 7b). The majority of recorded neurons are active neurons, and the fraction of active neurons was similar between conditioned and unconditioned mice (UC: 83.3%, 15 out of 18 neurons from 3 mice; C: 84.2%, 16 out of 19 neurons from 4 mice; *P* > 0.9999, Fisher’s exact test) (Fig. 7c). We next examined the intrinsic excitability of BF glutamatergic neurons by injecting depolarizing and hyperpolarizing currents into cells (Fig. 7d), and found that neither the membrane resistance (Fig. 7e) nor the number of spikes elicited by various steps of depolarizing currents (Fig. 7f) was altered in conditioned mice as compared with that in the unconditioned group. Besides, the resting membrane potential was also similar between these two groups of mice (UC: −52.64 ± 1.25, *n* = 18; C: −52.64 ± 0.96, *n* = 19, *P* = 0.9973, unpaired *t*-test). These measurements suggest that the SFC did not change the intrinsic excitability of BF glutamatergic neurons.

Next, we optically stimulated axon terminals of ChR2-expressing BF vGluT2 neurons and recorded LHb neurons to measure synaptic properties of BF-LHb glutamatergic projections (Fig. 7g). LHb neurons were held at different holding potentials to isolate AMPA receptor current and NMDA receptor current, respectively (Fig. 7h). We found that the AMPA/NMDA ratio in conditioned mice was almost two folds larger than that in the unconditioned mice (UC: 6.75 ± 1.06, *n* = 10 neurons from 4 mice; C: 11.96 ± 1.65, *n* = 8 neurons from 4 mice; *P* = 0.0138, unpaired *t*-test) (Fig. 7i), suggesting a functional augmentation of postsynaptic AMPA receptors. We also examined presynaptic alteration using a classical paired-pulse stimulation paradigm (Fig. 7j), and found a significant reduction in paired-pulse ratio (PPR) at both 50 ms (UC: 0.46 ± 0.07, *n* = 8 neurons from 3 mice; C: 0.24 ± 0.04, *n* = 11 neurons from 3 mice; *P* = 0.0092, unpaired *t*-test) and 100 ms (UC: 0.47 ± 0.06, *n* = 12 neurons from 3 mice; C: 0.28 ± 0.05, *n* = 12 neurons from 4 mice; *P* = 0.0300, unpaired *t*-test) inter-stimulus intervals in conditioned mice (Fig. 7k), suggesting a presynaptic increase in glutamate release probability following the SFC. Together, these observations suggest that the SFC potentiated BF-LHb glutamatergic functional connection via both pre- and post-synaptic mechanisms.

## Discussion

In the present study, we identified the BF as an important locus for social fear behavior regulation. This function is achieved through its glutamatergic neurons projecting to the LHb. These findings expand our understanding with regard to the function of BF region and complement the existing neural networks underlying fear behavioral manifestation. Besides, our data suggest that BF glutamatergic neurons and BF-LHb excitatory projections are potential therapeutic targets for alleviating maladaptive fear often observed in neuropsychiatric disorders including social phobia.

### The BF acts as an important locus for social fear behavior

Fear behavior relies on complex cognitive processes that involve sensory perception, risk assessment, behavioral decision making and so on^32–34^. It is therefore not surprising that fear expression is controlled by a number of distributed brain networks notably the amygdala, mPFC and ventral hippocampus among others^4^. Except for those well studied brain structures, increasing studies have been identifying other brain regions such as the PVT and hence expanding the existing fear regulation network^35,36^.

Traditionally, the BF contains subregions including the medial septum (MS), vertical and horizontal limbs of the diagonal band (VDB and HDB), the magnocellular preoptic nucleus (MCPO) and substantia innominata (SI). However, there is no consensus on the definition of the BF as it is anatomically complex and lacks clear boundaries. For instance, some studies suggest that the SI overlaps with the nucleus basalis, the ventral pallidum (VP), extended amygdala, and other structures in this region^37^, which may also be included in the BF. In this study, we targeted the BF subregions including the HDB, MCPO and part of SI, which were previously linked to multiple functions with cell-type specificity including sleep-wake control^13^, high-fat food intake^21^ and social behavior^29^. Notably, existing literature reveals distinct functions of BF subregions. For example, the SI encodes aversive information and bi-directionally modulates negative reinforcement learning^38^. In comparison, it was found that the SI is activated by aversive stimuli and inhibited by reward stimuli, which is involved in modulating depressive-like behaviors^39^. Moreover, the MS was found to mediate aversion induced by both auditory and somatosensory stimuli^40^, and can transmit innately aversive signals via a bottom-up multimodal sensory pathway, allowing animals to efficiently avoid unfavorable environments^41^. Except for these emotional functions, a recent study demonstrated that vGluT2 cells mainly in the VDB are important for anorexia-like phenotypes elicited by external threat^42^. In the present study, we discovered that social fear is associated with hyperactivity of the BF brain region. A large percentage of BF neurons in social fear conditioned mice exhibited elevated firing activity when confronted with a stimulus mouse (Fig. 1). On top of that, we further demonstrated the detailed cellular and circuit mechanisms that underlie BF’s function in fear regulation as discussed below. These findings together established that BF neurons are not only responsive to fearful stimuli but are required for fear expression.

### BF glutamatergic population is the key player in social fear

The BF is composed of three major neuronal populations: cholinergic neurons, glutamatergic neurons and GABAergic neurons^13^. Although the BF was previously implicated in a variety of fundamental functions including arousal, attention and cognitive processing, these functions were mostly attributed to its cholinergic population^12,13,16,17,43,44^. Based on several lines of evidence, our study unravels a crucial role of the BF in the regulation of fear expression, and such function is mediated by a non-cholinergic population, that is, glutamatergic neurons. First, selective inhibition of BF glutamatergic neurons but not the other two cell types in conditioned mice markedly increased the social interaction time assessed with a three-chamber social interaction test, indicating decreased social fear responses (Fig. 2, and Supplementary Figs. 4, 5). Second, during social fear expression, a lot more glutamatergic neurons in conditioned mice responded with increased firing rate than those in unconditioned control animals (Fig. 3). Interestingly, a recent study found that BF vGluT2 neurons are activated by threatening stimuli of different sensory modalities as well as tail suspension^45^, and optogenetic activation of BF vGluT2 but not the other two cell types produces place avoidance^45,46^. Therefore, it is likely that BF vGluT2 neurons generally encode sensory stimuli with negative valence, either congenital or acquired through learning. Except for social fear, we showed that BF vGluT2 neurons are also responsive to and necessary for non-social fear induced by odor-foot shock association (Supplementary Fig. 9). Therefore, BF vGluT2 neurons have a general role in both socially relevant fear behaviors and non-social fear learning. Although the present study focused on learned fear, it will be interesting to know whether BF glutamatergic neurons are equally important for innate fear as well.

There are complex local synaptic connections among subtypes of BF neurons, of which glutamatergic neurons and GABAergic neurons form reciprocal connections^13^. Intriguingly, recent studies including ours start to point out a functional dissociation between BF glutamatergic neurons and GABAergic neurons in valence detection and processing. For instance, optogenetic stimulation of GABAergic neurons and glutamatergic neurons in the medial septum (MS, a sub-region of BF) produces place preference and place avoidance, respectively^40^. Moreover, BF GABAergic neurons are activated by rewarding stimuli^41^, yet glutamatergic neurons are activated by aversive stimuli^45^. In terms of social behavior, we recently found that BF somatostatin (SST)-positive GABAergic neurons mediate prosocial behavior^22^. In striking contrast, here we discovered that BF glutamatergic neurons are responsible for social fear behavior (Figs. 2, 3). Therefore, the functional dissociation between these two non-cholinergic populations in the BF holds true in social behavioral regulation as revealed in our studies.

In addition to GABAergic neurons, BF glutamatergic neurons also make functional connections with cholinergic neurons^13^. Here we found that BF cholinergic neurons were not activated by social fear (Supplementary Fig. 2), and chemogenetic inhibition of cholinergic neurons did not attenuate social fear expression (Supplementary Fig. 4). This observation suggests that cholinergic population does not directly regulate fear expression of learned fear memory. However, it should be noted that prior studies have provided a comprehensive picture of the BF ChAT neurons underlying the acquisition and extinction of learned fear memory^16,17^. Therefore, even though cholinergic neurons do not participate in social fear expression, they are likely involved in the processes of social fear acquisition.

### The fear processing circuitry involving BF glutamatergic neurons

Neurons implement their functions through their innervations upon downstream targets. A neural tracing study revealed that BF glutamatergic neurons project to a wide range of brain regions including the MS, lateral hypothalamic area (LH), VTA and LHb^20^. In corroborating this study, we observed a dense distribution of axon terminals throughout the LHb area (Fig. 4c). Employing in vitro patch-clamp recordings, we further proved monosynaptic functional connectivity between BF glutamatergic neurons and LHb neurons (Fig. 4e). Consistently, we also found that axon terminals of BF glutamatergic neurons in the LHb were largely activated during social fear expression (Fig. 5 and Supplementary Fig. 11). The LHb represents a central hub for processing negative emotions, particularly depression^27^. Interestingly, emerging evidence starts to suggest that LHb plays a role in fear regulation as well. For example, LHb neurons are activated by looming, and inhibition of those neurons reduces the probability of escape to the visual stimuli^47^. Also, LHb neurons became gradually responsive to conditioning sound stimulus associated with an electric foot shock, and chemogenetic inhibition of LHb suppressed the freezing behavior in response to the conditioning stimulus^48,49^. Similarly, pharmacological inactivation of LHb also reduces social avoidance induced by social defeat stress^50^. Therefore, LHb neurons are not only able to encode aversive stimuli but also involved in the control of both innate and acquired fear responses.

Given that the fear encoding nature of LHb neurons, it is conceivable that excitation of BF glutamatergic neurons is able to drive LHb neurons and hence induce aversive responses. In line with this idea, inhibition of axon terminals of BF glutamatergic neurons in the LHb effectively alleviated social fear in social fear conditioned animals (Fig. 6). In addition to the hyperactivity of LHb-projecting glutamatergic neurons, functional potentiation of BF-LHb glutamatergic pathway was also observed in social fear conditioned mice (Fig. 7). As a consequence, both cellular excitation and synaptic potentiation could work together to drive animal’s social fear behavioral output. Therefore, the BF-LHb glutamatergic circuitry mediates social fear responses observed in the current study. Among several downstream targets of the LHb, the rostromedial tegmental nucleus (RMTg) and VTA participate in depression^27^, while the median raphe nucleus (MnR) and laterodorsal tegmental nucleus (LDT) have been recently shown to regulate fear behavior^51,52^. The exact downstream targets of the LHb in social fear regulation are to be determined.

Except for the LHb, the VTA is another downstream target of BF glutamatergic neurons^20^. The VTA is traditionally regarded as a brain structure important for emotions with positive valence^53,54^, yet recent studies suggest that the VTA is also implicated in encoding aversive stimuli^55,56^. Moreover, it is reported that activation of BF-VTA glutamatergic pathway produces place avoidance^45^. It was found that BF glutamatergic neurons were activated by various threatening stimuli and participated in the control of wakefulness^45^ and anorexia-like phenotypes^42^ via their projections to the VTA. However, neither the VTA-projecting BF glutamatergic neurons nor the axon terminals of BF glutamatergic neurons in the VTA are responsive to social stimuli in conditioned mice (Fig. 5 and Supplementary Fig. 11). Consistently, inhibition of BF-VTA glutamatergic projections did not affect social behavior in conditioned mice (Supplementary Fig. 14). Interestingly, we found that LHb-projecting and VTA-projecting BF glutamatergic neurons are largely non-overlapping (Fig. 5). Therefore, it is likely that there exist structural basis for the observed pathway differential neural activities and modulation effects in social fear. It seems that LHb-projecting and VTA-projecting BF glutamatergic neurons are differentially wired in the network to perform distinct functions.

Except for the BF-LHb glutamatergic projection, other circuits involving BF glutamatergic neurons and/or other cell types may also contribute to social fear modulation. For example, PAG as a well-known executive hub for defensive behavior that has been implicated in mediating social fear^26^ and other types of fear responses^32,57^. In addition, an excitatory projection from the BF to the lateral hypothalamus was linked to food-odor related stimuli, and potently elicited hypophagia^58^. It was found that GABAergic projections from the BF to the LHb modulate aggression reward^59^. Future studies are required to uncover comprehensive circuits of the BF in mediating social fear behaviors.

Animals make behavioral decision and hence take appropriate action based on environmental context. Then how is sensory information conveyed to BF glutamatergic neurons at the first place? Previous anatomic study demonstrated that BF glutamatergic neurons are directly contacted by a variety of upstream inputs^20^, some of which such as the amygdala are well known to participate in defensive behaviors^5^. Those brain structures could carry social threatening information and provide direct inputs to the BF. On the other hand, rodents rely on sensory information of different modalities, especially olfaction, to direct their social behaviors^60^. Given the crucial necessity of the piriform cortex (Pir) in olfactory processing^61^, it is also possible that a direct projection from the Pir to the BF relays aversive olfactory information to glutamatergic neurons and hence produces social fear behavior. In the future, it will be interesting to determine the exact upstream(s) of BF glutamatergic neurons in support of their fear driving function.

## Methods

### Animals

All the conducted experiments were approved by the Animal Care and Use Committee of Zhejiang University. Male vGluT2-Cre (Jackson Laboratory Strain 016963), vGAT-Cre (Jackson Laboratory Strain 016962), ChAT-Cre (Jackson Laboratory Strain 006410), C57BL/6 J wild-type mice (2-4 months old), and aggressive CD1 mice (7–9 months old, retired breeders) were used in this study. All animals were group housed in a 12 h light/dark cycle with food and water ad libitum except for those allocated to social fear conditioning (SFC) or implanted with chronic microelectrodes as described below. All mice were housed in a stable environment (23-25 °C ambient temperature and 50% humidity). All animals were habituated to the experimenter by handling for at least 3 consecutive days before behavioral tests.

### Social fear conditioning paradigm

The social fear conditioning was conducted to induce social fear in mice^23–25^. The conditioning chamber consisted of a white 30 × 30 × 50 cm Plexiglas box, with two identical acrylic bottomed cages placed at two opposing corners. The stainless-steel grid floor was connected to a shock delivery for foot shocks. Experimental mice were single-housed for one week before the conditioning started. On the first day, the experimental mouse was placed in the chamber 10 min for acclimation. Twenty-four hours later, mouse was placed in the chamber for another 5 min acclimation period, followed by introducing an unfamiliar male stimulus mouse in one of the cages placed at the corner. The experimental mouse was allowed to freely investigate the stimulus mouse for 2 min. Then during a 20 min social fear conditioning period, an electric foot shock (1 s, 0.6 mA) was delivered each time as the experimental mouse investigated the stimulus mouse. In another subset of experiment, the stimulus mouse was replaced with an object with odor (chocolate) during the fear conditioning day to develop a non-social learned fear mouse model.

### Sub-chronic social defeat paradigm

The sub-chronic social defeat was conducted as another paradigm to induce social fear in mice^26^. In brief, an unfamiliar aggressive male CD-1 intruder mouse was introduced to a wire cup placed in the home cage of singly-housed experimental mice for 5 min. Then, the wire cup was removed to allow the intruder invariably attacked the resident for another 10 min. The defeat procedure was repeated for three consecutive days. After a 7-day recovery, mice were subjected to a 10 min social preference-avoidance test, during that another unfamiliar aggressive CD1 was confined within a cylinder-shaped acrylic cage (10 cm in diameter) placed at the middle of the wall in a 50 × 50 cm open field arena. Time spent in 8 cm social zone and two opposite 9 cm corners were recorded and analyzed using EthoVision XT.

### Behavioral tests

#### Three-chamber social interaction test

The apparatus of three-chamber social interaction test consisted of a 60 × 40 × 20 cm Plexiglas box which was equally divided into three compartments. Dividing walls contained a 10 cm wide rectangular opening in the middle to enable free access of the testing mouse to each chamber. Two identical cylinder-shaped acrylic cages (10 cm in diameter) were placed in the corner of each side compartment. The lower 10 cm wall of the acrylic cages had evenly distributed slots (1 cm width) to allow the testing mouse to interact with the stimulus mouse which was placed inside the acrylic cage. A testing mouse was initially placed in the middle compartment to freely explore the entire apparatus for 10 min. Then, an unfamiliar stimulus mouse (age, sex and strain were matched with the testing mouse) was placed inside the acrylic cage in one of the side compartments designated as the social chamber. The opposite compartment with an empty acrylic cage was designated as the neutral chamber. The testing mouse was allowed to freely explore the apparatus for another 10 min, during that the location and overall movement of the testing mouse were automatically recorded by a video camera and analyzed with the EthoVision XT video tracking software (Noldus, Netherland). The amount of time that the testing mouse spent in each chamber or the immediate vicinity (8 cm) of the stimulus cages (termed as “zone”) was measured. The social interaction index was calculated as the difference in the time spent in the social and neutral chambers (zones), divided by the sum of the time spent in both chambers (zones). Social approach times, approach speed and stretched postures behavior were quantified during the 10 min interaction period. After each session, the apparatus and cylinder cages were thoroughly cleaned with 75% ethanol to prevent olfactory cue bias.

#### Open field test

An open field arena (42 × 42 × 50 cm) was used to evaluate locomotion and anxiety-like behaviors of the testing mouse. The testing mouse was initially placed in the center of the arena and allowed to freely explore the arena for 10 min, during that the movement of the mouse was automatically recorded and analyzed by the Ethovision XT video tracking system. The total distance traveled, time spent in the center zone (21 cm × 21 cm square) and the number of center entries were analyzed. The arena was thoroughly cleaned with 75% ethanol between tests.

### Virus injection

AAV2/9-EF1α-DIO-EYFP (6.10 × 10^12^ genomic copies per ml), AAV2/9-EF1α-DIO-mCherry (3.08 × 10^12^ genomic copies per ml), AAV2/9-EF1α-double floxed-hChR2(H134R)-mCherry (1.70 × 10^12^ genomic copies per ml), AAV2/9-EF1α-DIO-eNpHR3.0-mCherry (1.35 × 10^12^ genomic copies per ml), AAV2/5-hSyn-DIO-hM4D(Gi)-mCherry (3.80 × 10^12^ genomic copies per ml), AAV2/9-hSyn-DIO-hM4D(Gi)-EGFP (2.60 × 10^12^ genomic copies per ml), AAV2/9-EF1α-DIO-GtACR1-EGFP (4.50 × 10^12^ genomic copies per ml), AAV2/9-EF1α-DIO-H2B-BFP-T2A-TVA (2.60 × 10^12^ genomic copies per ml), RV-EnvA-ΔG-DsRed (2 × 10^9^ pfu/ml), RV CVS-EnvA-ΔG-tdTomato (2 × 10^9^ pfu/ml), RV CVS-EnvA-ΔG-EGFP (2 × 10^9^ pfu/ml), RV CVS-EnvA-ΔG-GCaMP6s (3 × 10^8^ pfu/ml), AAV2/9-hSyn-DIO-jGCaMP8s (2.01 × 10^12^ genomic copies per ml) and AAV2/9-hSyn-FLEX-mGFP-Synaptophysin-mRuby (4.80 × 10^12^ genomic copies per ml) were purchased from Taitool (Shanghai), BrainCase (Shenzhen) or BrainVTA (Wuhan). The virus solutions were aliquoted over ice into 4 µl vials and stored immediately in a −80 °C freezer after arrival.

Mice were anesthetized with isoflurane (4% for induction, 1% for maintenance) and placed in a stereotaxic frame (Stoelting Co., IL, USA). The skull was exposed and a small craniotomy was made with a dental drill under a surgical microscope. The following coordinates relative to bregma were used to target the caudal portion of the BF (including the horizontal limb of the diagonal band of Broca, magnocellular preoptic nucleus and substantia innominata) (AP: +0.15 mm; ML: ±1.40 mm; DV: −5.70 mm), the LHb (AP: −1.60 mm; ML: ±0.45 mm; DV: −2.60 mm), and the VTA (AP: −3.00 mm; ML: ±0.75 mm; DV: −4.50 mm). A small volume of virus solution (50 to 100 nL) was injected into the target regions at a slow rate (30–50 nL/min) using a glass micropipette (tip diameter ~15 μm) attached to a Nanoliter pressure microsyringe pump and a micro controller (World Precision Instrument). The injection pipette was remained in place for another 10 min at the end of the infusion. Experiments were conducted at least 1 week, 4 weeks and 6 weeks after virus injection for RV detection, BF cell bodies and axon terminals manipulation or fiber photometry recording, respectively.

### Optical fiber implantation

The optical fiber implantation was carried out at least 2 weeks ahead of behavioral testing. An optical fiber (inside diameter (I.D.): 200 μm, numerical aperture (NA): 0.37; Inper Inc., Hangzhou) was placed in a ceramic ferrule (outside diameter (O.D.): 1.25 mm) and inserted toward the targeted brain regions (with the tip 200 μm above) through the craniotomy. For optogenetic manipulation, two optical fibers were implanted to target bilateral BF, LHb or VTA using the following coordinates: BF (AP: +0.15 mm; ML: ±1.40 mm; DV: −5.50 mm), LHb (AP: −1.60 mm; ML: ±0.88 mm at a 10° angle; DV: −2.43 mm), and VTA (AP: −3.00 mm; ML: ±1.54 mm at a 10° angle; DV: −4.35 mm). The ceramic ferrule was secured to the skull using 3 M Vetbond tissue adhesive and dental cement.

### Optogenetic manipulation

For optogenetic manipulation experiments, optical fibers were connected to a 589 nm or 470 nm laser generator (Inper Inc., Hangzhou). The laser beam was split into two beams through a commutator and connected to the implanted optical fibers with an optical fiber sleeve. The testing mouse was then placed in the three-chamber, two-chamber, or open field apparatus for behavioral tests. For optogenetic manipulation, the power of photostimulation was 1–5 mW measured at the tip of the optical fiber and was constantly delivered (for NpHR-expressing studies) or flashed at 20 Hz with 10 ms pulses (for GtACR1- or ChR2-expressing studies) for the entire duration of the test in this study. Control mice injected with mCherry or EYFP virus underwent the same procedure and received the same amount of photostimuli.

### Chemogenetic manipulation

For chemogenetic inhibition experiments, Clozapine-N-Oxide (CNO; Sigma, C0832) was dissolved in saline (0.9% NaCl solution) to a concentration 0.75 mg/mL. CNO (5 mg/kg body weight) was administered intraperitoneally (*i.p*. injection) to the testing mice 50 min prior to behavioral testing. To avoid potential confounding effect of CNO metabolite, the control mice injected with EYFP under the same operation was performed before behavioral tests.

### Fiber photometry

To record fluorescent signals emitted by GCaMP (a genetically encoded calcium indicator), a fiber photometry system (ThinkerTech, Nanjing) was employed^24,29^. Briefly, laser beam generated by a 488 nm laser (OBIS 488LS; Coherent) was reflected by a dichroic mirror (MD498; Thorlabs) with a low-level power (20–40 μW) at the tip of optical fiber to minimize bleaching and kept constant during a recording session. The fluorescent signals were bandpass filtered, converted to voltage signals, digitalized at 100 Hz and then recorded using a custom-written script in LabView. Animals underwent fiber photometry recording were conducted in a three-chamber social interaction or open field social preference-avoidance (for defeated mice) task. Behavior-coupled fluorescent events were determined by peri-stimulus time histograms (PSTH) calculated as ΔF/F. Mice that expressed EYFP in the BF vGluT2 neurons were also recorded by fiber photometry during social fear behavioral expression to exclude the potential influence of motion artifacts. Spontaneous calcium event analysis was performed for each photometric recording data to verify the effectiveness of the data. Data with spontaneous calcium events during the first 10 min habituation session, identified as ΔF/F rose above 2.91 median absolute deviations from baseline^62^, were included for further analysis. Post-mortem examination was utilized to confirm GCaMP expression and optical fiber placement.

### In vivo electrophysiological recording

#### Surgical implantation of tetrodes/optrodes

After a craniotomy (0.8–1.0 mm in diameter) and the dura mater was removed, a custom-made 8 movable tetrodes array was inserted into the BF. Each tetrode was made of four twisted fine platinum/iridium wires (12.5 µm diameter, California Fine Wire) and threaded through a silica tube (75 µm inner, 152 µm outer diameter; Polymicro Technologies). Each wire was soldered to a 36-pin connector (Omnetics, USA), and the other four pins were soldered with two pairs of copper micro-wires ( ~ 100 µm diameter) for grounding and reference. The wire tips were electroplated with gold by passing cathodal current to reduce impedance to a final value of 300–400 kΩ at 1 kHz by controlling of a NanoZ impedance testing software (Plexon Inc., USA). For optogenetic tagging of vGluT2 neurons in a subset of vGluT2-Cre mice with AAV-DIO-ChR2-mCherry expressed in the BF, tetrodes were replaced with optrodes which consisted of one optic fiber surrounded by 8 tetrodes, with the tips of microwires protruding ~ 300 µm beyond the optic fiber. The tetrode/optrode was fixed to the skull with four miniature skull screws, cyanoacrylate glue and dental cement. Mice were then placed on a heating pad to wake up and thereafter single-housed.

#### Electrophysiological data acquisition

After a 7-day recovery from the surgery of electrode implantation, mice were acclimated to the headstage and cables connected to the electrode on their heads for several days before electrophysiological data acquisition. To explore the BF neuronal activity in social interaction behavior, recordings were performed when subject mouse was freely exploring a single-chamber social approach-avoidance apparatus (20 × 40 × 20 cm), where a small cylinder-shaped acrylic cage was placed at the middle of one side. A testing mouse with micro-electrode implantation was placed into the apparatus and allowed to acclimate for 10 min. Then an unfamiliar stimulus mouse was introduced within the cylinder cage, and the testing mouse was allowed to freely explore the entire apparatus for another 10 min, during those multichannel electrophysiological signals and behavioral data were simultaneously recorded using an OmniPlex neural recording data acquisition system and a CinePlex behavioral research system (Plexon Inc., USA), respectively. Spiking activities with amplitude larger than 3.5 standard deviations from the mean were digitized at 40 kHz, bandpass filtered from 250 Hz to 8000 Hz, and stored on a computer for further offline analysis. At the end of each daily experiment, tetrodes were lowered by ~40 μm so that different units were recorded across successive behavioral tests. Animals used for in vivo recording experiments were perfused after recordings and the positions of the recording site were verified.

#### Spike sorting and firing rate analysis

Spike signals were sorted using Offline Sorter software (Plexon Inc.). Principal component analysis was automatically calculated and plotted in a three-dimensional space. Manual checking was then performed to ensure that the spike waveforms were consistent and that the cluster boundaries were clearly separated. If the waveforms in a cluster exhibit a clear refractory period of more than 2 ms, they were considered to be generated from a single unit. Only well-isolated units (L ratio < 0.2, isolation distance > 15) were included in the data analysis. Units with mean firing rate higher than 0.5 Hz were included for further analysis.

To quantify the difference in firing rates during social interaction between unconditioned and conditioned mice, spike rate during a 2 s period before the onset of a social approach epoch was defined as baseline firing rate, and that during a 2 s period after social interaction started was defined as firing rate of social investigation. To avoid confounding effect of the difference in social interaction times between unconditioned and conditioned mice, the first six interaction bouts for each behavioral test were averaged to calculate a mean firing rate for both baseline and social interaction. Significant changing of firing rate between baseline and social interaction was determined by paired *t*-test for each single unit. To ensure accuracy, only interactions with duration of longer than 2 s and an interval from previous visit of more than 2 s were used for spike rate analysis.

#### Optogenetic tagging of vGluT2 neurons

For electrophysiological identification of vGluT2 neurons, blue light pulses (470 nm, 1–2 ms duration, 0.1–1.0 mW at fiber tip) were delivered at the end of each recording session at high frequencies (10, 20 Hz). Units were considered as light responsive if they exhibited time-locked spiking with high reliability ( > 90%), and short first-spike latency ( < 5 ms) upon light pulses illumination.

### Brain slices electrophysiological recording

#### Brain slice preparation

vGluT2-Cre mice expressing AAV2/9-DIO-ChR2-mCherry, AAV2/5-DIO-hM4D(Gi)-mCherry or AAV2/9-DIO-eNpHR3.0-mCherry in the BF for at least 6 weeks were used for brain slice recordings. Mice were deeply anesthetized with pentobarbital sodium and transcardially perfused 20 mL oxygenated ice-cold modified ACSF (composition: 87 mM NaCl、2.5 mM KCl、1.25 mM NaH_2_PO_4_、26 mM NaHCO_3_、1 mM CaCl_2_·2H_2_O、2 mM MgSO_4_·7H_2_O、75 mM sucrose、10 mM glucose). Coronal slices containing the BF, LHb or VTA at 300 μm thickness were dissected by a vibrating microtome (Leica VT1200s, Germany). The brain slices recovered for 30 min with water bath at 32 °C, then incubated at room temperature for at least 1 h with oxygenated standard ACSF (composition: 119 mM NaCl、2.5 mM KCl、1.25 mM NaH_2_PO_4_、24 mM NaHCO_3_、2 mM CaCl_2_·2H_2_O、2 mM MgSO_4_·7H_2_O、12.5 mM glucose; osmolarity: 300–305 mOsm/kg). All chemicals used in slice preparation were purchased from Sigma.

#### Whole-cell patch clamp recordings

A brain slice was transferred to the recording chamber (Warner Instruments Inc., Hamden, CT) and was continuously perfused with oxygenated standard ACSF at a rate of 2 mL/min. Whole-cell recordings were obtained from visually identified neurons using a 40× water-immersion objective on an upright microscope (Olympus, Japan) equipped with IR-DIC optics and a CCD camera. Patch electrodes (3–6 MΩ) were pulled from borosilicate glass capillaries (1.5 mm O.D.) with a P-97 puller (Sutter Instrument, Novata, CA), and filled with internal solution (composition: 130 mM K-gluconate, 5 mM KCl, 2 mM MgCl_2_·6H_2_O,0.6 mM EGTA, 10 mM HEPES, 4 mM Mg-ATP and 0.3 mM Na-GTP; pH, 7.2–7.3; osmolarity, 285-290 mOsm/kg) for current-clamp recordings. For voltage-clamp recordings, the patch electrodes were filled with internal solution (composition: 110 mM CsMeSO_3_, 20 mM TEA-Cl, 15 mM CsCl, 0.5 mM EGTA, 10 mM HEPES, 4 mM Mg-ATP, 0.3 mM Na-GTP, 4 mM QX-314, and 1 mM spermine; pH, 7.2–7.3; osmolarity, 285–290 mOsm/kg). Data were collected using a Multiclamp 700B amplifier (Molecular Devices, Sunnyvale, CA), low-pass filtered at 5 kHz and digitally samples at 10 kHz on-line and analyzed off-line with pClamp9 software (Molecular Devices). Only cells with stable series resistance ( < 20% change) were used for analysis.

To examine electrophysiological characteristics of BF vGluT2 neurons, spontaneous activity was monitored in a current-clamp mode after stable recording formed. Neurons were roughly categorized into active or silent group according to whether they have spontaneous action potentials. For measuring spike activity, steady-state currents were injected in 20 pA increments from 0 to 120 pA. Membrane resistance was calculated from the change in voltage evoked after a -30 pA hyperpolarizing current injection. To evaluate the validity of CNO on hM4D(Gi)-expressing neurons, current-clamp mode with 80 pA current injection was conducted. Resting membrane potential and number of current-induced action potentials were compared before and after CNO (10 µM) administration. To evaluate the validity of NpHR in BF vGluT2 neurons, current-clamp mode with 100 pA current injection was conducted and delivered a yellow light stimulation (589 nm, 250 ms). To clarify the characteristics of BF-LHb/VTA glutamatergic connection, LHb or VTA neurons were held at −70 mV in a voltage-clamp mode and blue light pulses (470 nm, 2 ms pulse width) generated by LED (Mightex, Toronto, Canada) were delivered every 20 s to stimulate the axon terminals of BF vGluT2 neurons projecting to the LHb/VTA. Tetrodotoxin (TTX, 1 μM, Sigma-Aldrich) and 4-aminopyridine (4-AP, 100 μM, Sigma-Aldrich) were added for verifying monosynaptic responses evoked by blue light pulse. 6,7-dinitroquinoxaline-2,3-dione (DNQX, 10 µM, Sigma-Aldrich) and DL-2-Amino-5-phosphonopentanoic acid (AP-5, 20 µM, Sigma-Aldrich) were bath administrated to determine excitatory connections in BF-LHb/VTA glutamatergic pathway. To examine the paired-pulse ratio (PPR) of the excitatory postsynaptic currents (EPSCs), cells were held at -70 mV in a voltage-clamp mode and two blue light pulses (470 nm, 2 ms pulse width) with 50 or 100 ms interval were delivered every 20 s. The PPR was defined as amplitude of the second EPSC divided by the first one. To record AMPA current, cells were held at −70 mV in a voltage-clamp mode, and then slowly changed the holding potential to + 40 mV to record NMDA current. To minimize the influence of AMPA current, the amplitude of NMDA current was analyzed at 50 ms after stimuli.

### Virus tracing

For anterograde tracing, AAV2/9-hSyn-FLEX-mGFP-Synaptophysin-mRuby was injected to trace the downstream outputs of BF vGluT2 neurons. At 6 weeks after virus injection, mice were euthanized and perfused. Then 40 µm frozen brain sections were collected. Fluorescent images of BF and LHb were captured using a confocal microscope (Olympus FV1200) with a 10× objective. Boundaries of brain regions were defined based on DAPI staining and The Mouse Brain in Stereotaxic Coordinates, 3rd Edition.

For retrograde tracing, AAV2/9-EF1α-DIO-H2B-BFP-T2A-TVA was injected into the BF of vGluT2-Cre mice. Six weeks later, 30 nL of EnvA-pseudotyped rabies virus (RV-EnvA-ΔG-DsRed/RV CVS-EnvA-ΔG-tdTmato and RV CVS-EnvA-ΔG-EGFP) were injected into the VTA or LHb using the previously defined coordinates for tracing VTA- and LHb-projecting BF vGluT2 neurons, respectively. After euthanized and perfused, 40 µm frozen brain sections were collected and a confocal microscope was used to capture figures. Scans from each channel were collected in multitrack mode to avoid cross-talk between channels. To evaluate potential neuronal loss after RV infection, slices were stained with NeuroTrace 640/660 Deep-Red Fluorescent Nissl Stain (diluted 1:1000 in PBS, Cat. # N21483, ThermoFisher Scientific). The number of neurons between the RV-GCaMP recording side and the contralateral side were calculated by ImageJ software.

### Statistical analyses

Data are presented as mean ± SEM unless otherwise noted. Statistical comparisons were conducted with GraphPad Prism 9 (La Jolla, CA). Generally, one-way or two-way ANOVA followed by Bonferroni *post hoc* test was performed for multiple comparisons, student’s *t*-test was performed for two-group comparisons, and Fisher’s exact test was performed for analysis of contingency tables as specified in figure legends. A *P*-value of < 0.05 was considered as the criterion for a significant statistical difference. *P < 0.05, **P < 0.01, ***P < 0.001, ****P < 0.0001.

### Reporting summary

Further information on research design is available in the Nature Portfolio Reporting Summary linked to this article.

### Supplementary information

Supplementary Information

Peer Review File

Reporting Summary

### Source data

Source Data

## Data Availability

There are no restrictions on data availability in the manuscript. The data generated in this study are provided in the Source Data file. Source data are provided with this paper.

## References

[1] Leichsenring F, Leweke F (2017). Social Anxiety Disorder. New Engl. J. Med..

[2] Nees F, Witt SH, Flor H (2018). Neurogenetic Approaches to Stress and Fear in Humans as Pathophysiological Mechanisms for Posttraumatic Stress Disorder. Biol. Psychiatry.

[3] Sheynin J (2017). Greater avoidance behavior in individuals with posttraumatic stress disorder symptoms. Stress.

[4] Herry C, Johansen JP (2014). Encoding of fear learning and memory in distributed neuronal circuits. Nat. Neurosci..

[5] Phelps EA, LeDoux JE (2005). Contributions of the amygdala to emotion processing: from animal models to human behavior. Neuron.

[6] Tovote P, Fadok JP, Luthi A (2015). Neuronal circuits for fear and anxiety. Nat. Rev. Neurosci..

[7] Burgos-Robles A, Vidal-Gonzalez I, Quirk GJ (2009). Sustained Conditioned Responses in Prelimbic Prefrontal Neurons Are Correlated with Fear Expression and Extinction Failure. J. Neurosci..

[8] Karalis N (2016). 4-Hz oscillations synchronize prefrontal-amygdala circuits during fear behavior. Nat. Neurosci..

[9] Do-Monte FH, Quinones-Laracuente K, Quirk GJ (2015). A temporal shift in the circuits mediating retrieval of fear memory. Nature.

[10] Kjelstrup KG (2002). Reduced fear expression after lesions of the ventral hippocampus. Proc. Natl. Acad. Sci. USA.

[11] De Oca BM, DeCola JP, Maren S, Fanselow MS (1998). Distinct regions of the periaqueductal gray are involved in the acquisition and expression of defensive responses. J Neurosci.

[12] Ballinger EC, Ananth M, Talmage DA, Role LW (2016). Basal Forebrain Cholinergic Circuits and Signaling in Cognition and Cognitive Decline. Neuron.

[13] Xu M (2015). Basal forebrain circuit for sleep-wake control. Nat. Neurosci..

[14] Conner JM, Culberson A, Packowski C, Chiba AA, Tuszynski MH (2003). Lesions of the Basal forebrain cholinergic system impair task acquisition and abolish cortical plasticity associated with motor skill learning. Neuron.

[15] Guo W, Robert B, Polley DB (2019). The Cholinergic Basal Forebrain Links Auditory Stimuli with Delayed Reinforcement to Support Learning. Neuron.

[16] Crimmins BE (2023). Basal forebrain cholinergic signaling in the basolateral amygdala promotes strength and durability of fear memories. Neuropsychopharmacology.

[17] Knox D (2016). The role of basal forebrain cholinergic neurons in fear and extinction memory. Neurobiol. Learn Mem..

[18] Rabellino D, Densmore M, Frewen PA, Theberge J, Lanius RA (2016). The innate alarm circuit in post-traumatic stress disorder: Conscious and subconscious processing of fear- and trauma-related cues. Psychiatry Res. Neuroimaging..

[19] Zhu X (2023). Functional Connectivity Between Basal Forebrain and Superficial Amygdala Negatively Correlates with Social Fearfulness. Neuroscience.

[20] Do JP (2016). Cell type-specific long-range connections of basal forebrain circuit. eLife.

[21] Zhu C (2017). Somatostatin Neurons in the Basal Forebrain Promote High-Calorie Food Intake. Cell Rep..

[22] Li YD (2021). Ventral pallidal GABAergic neurons control wakefulness associated with motivation through the ventral tegmental pathway. Mol. Psychiatry.

[23] Toth I, Neumann ID, Slattery DA (2012). Social fear conditioning: a novel and specific animal model to study social anxiety disorder. Neuropsychopharmacology: official publication of the American College of Neuropsychopharmacology.

[24] Xu H (2019). A Disinhibitory Microcircuit Mediates Conditioned Social Fear in the Prefrontal Cortex. Neuron.

[25] Zheng J, Tian Y, Xu H, Gu L, Xu H (2021). A Standardized Protocol for the Induction of Specific Social Fear in Mice. Neurosci. Bull..

[26] Franklin TB (2017). Prefrontal cortical control of a brainstem social behavior circuit. Nat. Neurosci..

[27] Hu H, Cui Y, Yang Y (2020). Circuits and functions of the lateral habenula in health and in disease. Nat. Rev. Neurosci..

[28] Brandao ML, Coimbra NC (2019). Understanding the role of dopamine in conditioned and unconditioned fear. Rev. Neurosci..

[29] Wang J (2021). Basal forebrain mediates prosocial behavior via disinhibition of midbrain dopamine neurons. Proc. Natl. Acad. Sci. USA..

[30] Diaz V, Lin D (2020). Neural circuits for coping with social defeat. Curr. Opin. Neurobiol..

[31] Wei D, Talwar V, Lin D (2021). Neural circuits of social behaviors: Innate yet flexible. Neuron.

[32] Gross CT, Canteras NS (2012). The many paths to fear. Nat. Rev. Neurosci..

[33] Adolphs R (2013). The Biology of Fear. Curr. Biol..

[34] LeDoux JE, Pine DS (2016). Using Neuroscience to Help Understand Fear and Anxiety: A Two-System Framework. Am. J Psychiatry.

[35] Beas BS (2018). The locus coeruleus drives disinhibition in the midline thalamus via a dopaminergic mechanism. Nat. Neurosci..

[36] Penzo MA (2015). The paraventricular thalamus controls a central amygdala fear circuit. Nature.

[37] Agostinelli LJ, Geerling JC, Scammell TE (2019). Basal forebrain subcortical projections. Brain Struct. Func..

[38] Cui Y (2017). A Central Amygdala-Substantia Innominata Neural Circuitry Encodes Aversive Reinforcement Signals. Cell Rep..

[39] Cui Y (2022). Reward ameliorates depressive-like behaviors via inhibition of the substantia innominata to the lateral habenula projection. Sci. Adv..

[40] Zhang GW (2018). Transforming Sensory Cues into Aversive Emotion via Septal-Habenular Pathway. Neuron.

[41] Shen L (2022). A bottom-up reward pathway mediated by somatostatin neurons in the medial septum complex underlying appetitive learning. Nat. Commun..

[42] Cai J (2023). An excitatory projection from the basal forebrain to the ventral tegmental area that underlies anorexia-like phenotypes. Neuron.

[43] Jones BE (2008). Modulation of cortical activation and behavioral arousal by cholinergic and orexinergic systems. Ann. NY Acad. Sci..

[44] Thiele A, Bellgrove MA (2018). Neuromodulation of Attention. Neuron.

[45] Cai P (2022). A glutamatergic basal forebrain to midbrain circuit mediates wakefulness and defensive behavior. Neuropharmacology.

[46] McKenna JT (2021). Characterization of basal forebrain glutamate neurons suggests a role in control of arousal and avoidance behavior. Brain Struct. Funct..

[47] Lecca S (2020). Heterogeneous Habenular Neuronal Ensembles during Selection of Defensive Behaviors. Cell Rep..

[48] Wang D (2017). Learning shapes the aversion and reward responses of lateral habenula neurons. Elife.

[49] Velazquez-Hernandez, G. & Sotres-Bayon, F. Lateral Habenula Mediates Defensive Responses Only When Threat and Safety Memories Are in Conflict. *eNeuro* **8**10.1523/ENEURO.0482-20.2021 (2021). 10.1523/ENEURO.0482-20.2021 PMC8059882 33712440

[50] Sachs BD, Ni JR, Caron MG (2015). Brain 5-HT deficiency increases stress vulnerability and impairs antidepressant responses following psychosocial stress. Proc. Natl. Acad. Sci. USA..

[51] Yang H (2016). Laterodorsal tegmentum interneuron subtypes oppositely regulate olfactory cue-induced innate fear. Nat. Neurosci..

[52] Szonyi A (2019). Median raphe controls acquisition of negative experience in the mouse. Science.

[53] Schultz W, Dayan P, Montague PR (1997). A neural substrate of prediction and reward. Science.

[54] Fiorillo CD (2013). Two dimensions of value: dopamine neurons represent reward but not aversiveness. Science.

[55] de Jong JW (2019). A Neural Circuit Mechanism for Encoding Aversive Stimuli in the Mesolimbic Dopamine System. Neuron.

[56] Barbano MF (2020). VTA Glutamatergic Neurons Mediate Innate Defensive Behaviors. Neuron.

[57] Tovote P (2016). Midbrain circuits for defensive behaviour. Nature.

[58] Patel JM (2019). Sensory perception drives food avoidance through excitatory basal forebrain circuits. eLife.

[59] Golden SA (2016). Basal forebrain projections to the lateral habenula modulate aggression reward. Nature.

[60] Chen P, Hong W (2018). Neural Circuit Mechanisms of Social Behavior. Neuron.

[61] Bekkers JM, Suzuki N (2013). Neurons and circuits for odor processing in the piriform cortex. Trends Neurosci..

[62] Bavley CC (2020). Cocaine- and stress-primed reinstatement of drug-associated memories elicit differential behavioral and frontostriatal circuit activity patterns via recruitment of L-type Ca^2+^ channels. Mol. Psychiatry.

