# Supplementary material for: The basal forebrain to lateral habenula circuitry mediates social behavioral maladaptation
Source: Nat Commun. 2024 May 13;15:4013. doi: 10.1038/s41467-024-48378-y (PMC11091113; doi:10.1038/s41467-024-48378-y)
Supplement: Supplementary file 1 — Supplementary Information [file 41467_2024_48378_MOESM1_ESM.pdf]

Supplementary figures and legends

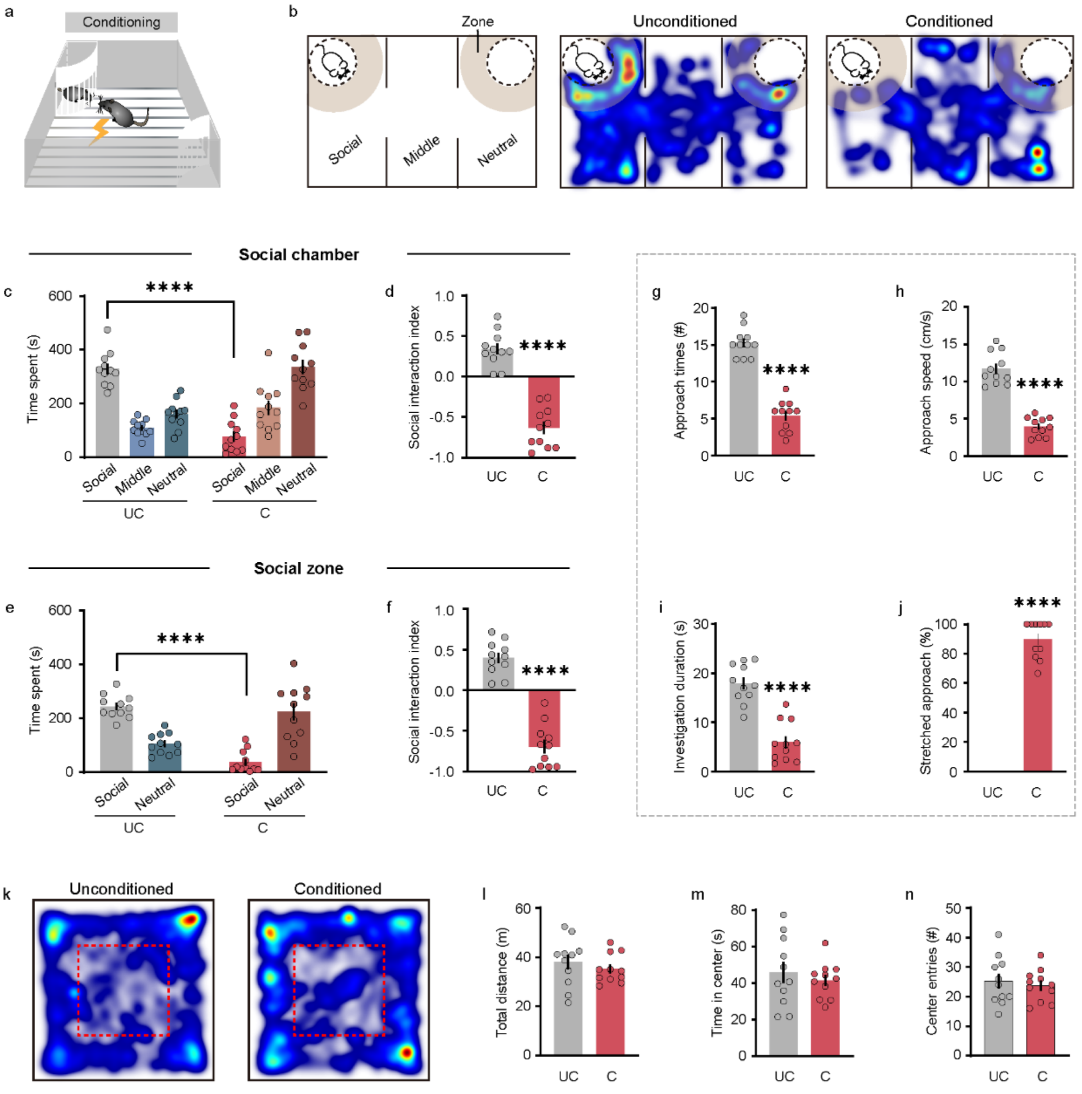

Supplementary Fig. 1 Induction of social fear by social fear conditioning (SFC).

(**a**) Schematic diagram of the SFC paradigm.

(**b**) Schematic diagram of the three-chamber social interaction test (left), and representative heatmaps showing the movement traces of an unconditioned mouse (middle) and a conditioned mouse (right).

(**c**) Quantification of time spent in each chamber by unconditioned (UC) mice and conditioned (C) mice. n = 11 for each group. $F_{interaction}(2, 40) = 41.19$, $P < 0.0001$; $F_{chamber}(2, 40) = 8.58$, $P = 0.0008$; $F_{group}(1, 20) = 0.24$, $P = 0.6265$; time spent in social

chamber (UC vs. C): $P < 0.0001$, two-way ANOVA followed by Bonferroni's multiple comparisons.

(**d**) Social interaction index was significantly decreased in conditioned mice compared to that in the unconditioned mice. $P < 0.0001$, two-sided unpaired *t* test.

(**e**) Quantification of time spent in social zone and neutral zone (8 cm vicinity of the social or neutral cage) in unconditioned and conditioned mice. $F_{interaction}(1, 20) = 60.59$, $P < 0.0001$; $F_{zone}(1, 20) = 1.31$, $P = 0.2657$; $F_{group}(1, 20) = 6.46$, $P = 0.0194$; time spent in social zone (UC vs. C): $P < 0.0001$, two-way ANOVA followed by Bonferroni's multiple comparisons.

(**f**) Social interaction index was significantly decreased in conditioned mice compared to that in the unconditioned mice. $P < 0.0001$, two-sided unpaired *t* test.

(**g-j**) Quantification of the approach times (**g**) ($P < 0.0001$), approach speed (**h**) ($P < 0.0001$), duration of social investigation (**i**) ($P < 0.0001$), and proportion of stretched postures (**j**) ($P < 0.0001$) in unconditioned and conditioned mice. n = 11 for each group. Two-sided unpaired *t* test.

(**k**) Representative heatmaps showing the movement traces of an unconditioned mouse (left) and a conditioned mouse (right) in open field test.

(**l-n**) Quantification of the total distance moved (**l**) ($P = 0.4383$), time spent in center (**m**) ($P = 0.5083$), number of entries into the center (**n**) ($P = 0.6256$) in unconditioned and conditioned mice. n = 11 for each group. Two-sided unpaired *t* test.

Error bars indicate mean ± SEM. ****$P < 0.0001$. Source data are provided as a Source Data file.

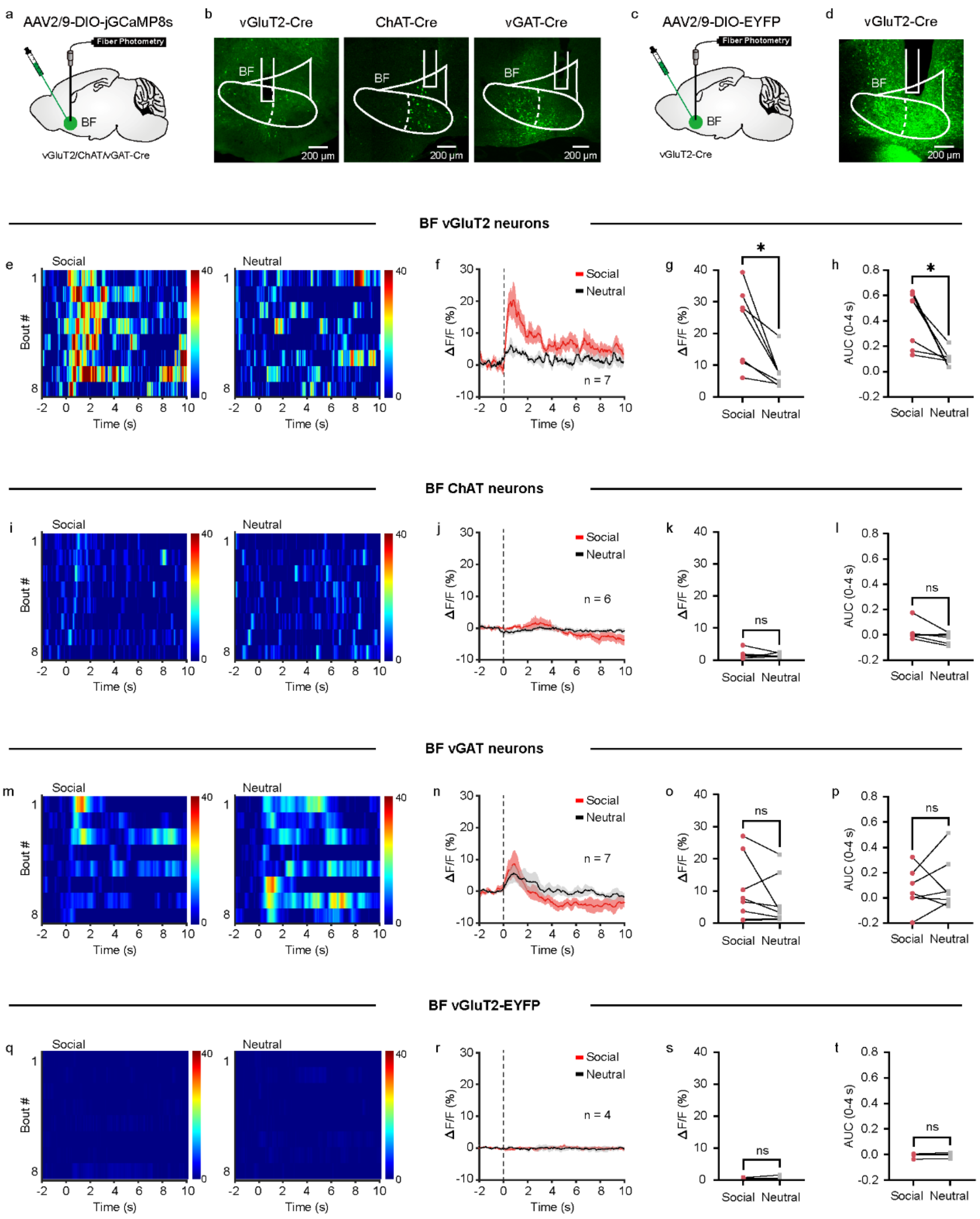

Supplementary Fig. 2 Activity of BF vGluT2, ChAT, and vGAT neurons during social fear expression.

(**a**) Schematic illustration of AAV-hSyn-DIO-jGCaMP8s virus injection in the BF and fiber photometry recording of three types of neurons in vGluT2-, ChAT-, or vGAT-Cre mouse.

(**b**) Representative images showing GCaMP virus expression and placement of optical

fiber in the BF of a vGluT2-Cre (left), ChAT-Cre (Middle), or vGAT-Cre mouse (right).

(**c**) Schematic illustration of AAV-hSyn-DIO-EYFP virus injection and fiber photometry recording in the BF of vGluT2-Cre mouse.

(**d**) Representative images showing EYFP virus expression and placement of optical fiber in the BF of vGluT2-Cre mouse.

(**e**) Heatmap of Ca^{2+} signals from BF vGluT2 neurons in a conditioned mouse. Ca^{2+} signals were aligned to the onset of individual interactions with a social stimulus (left) or an empty neutral cage (right). Each row represents one interaction bout, and the color scale at the right indicates ΔF/F.

(**f**) The peri-event plot of the mean BF vGluT2 Ca^{2+} transient during social or neutral interactions for the entire test group (n = 7 mice). The thick line indicates the mean, and the shaded area indicates SEM.

(**g**) Statistical comparison of BF vGluT2 peak fluorescence signals between social and neutral interactions. n = 7 mice. P = 0.0136, two-sided paired t test.

(**h**) Statistical comparison of area under curve (0-4 s) of BF vGluT2 fluorescence signals. P = 0.0120, two-sided paired t test.

(**i-l**) The same as (**e-h**) but for recordings made in the ChAT-Cre mice (n = 6 mice). (**k**) P = 0.6746; (**l**) P = 0.1001, two-sided paired t test.

(**m-p**) The same as (**e-h**) but for recordings made in the vGAT-Cre mice (n = 7 mice). (**o**) P = 0.2283; (**p**) P = 0.6112, two-sided paired t test.

(**q-t**) The same as (**e-h**) but for recordings made in the BF vGluT2 EYFP-expressing mice (n = 4 mice). (**s**) P = 0.6718; (**t**) P = 0.3699, two-sided paired t test.

Error bars indicate mean ± SEM. ns, no significant difference, *P < 0.05. Source data are provided as a Source Data file.

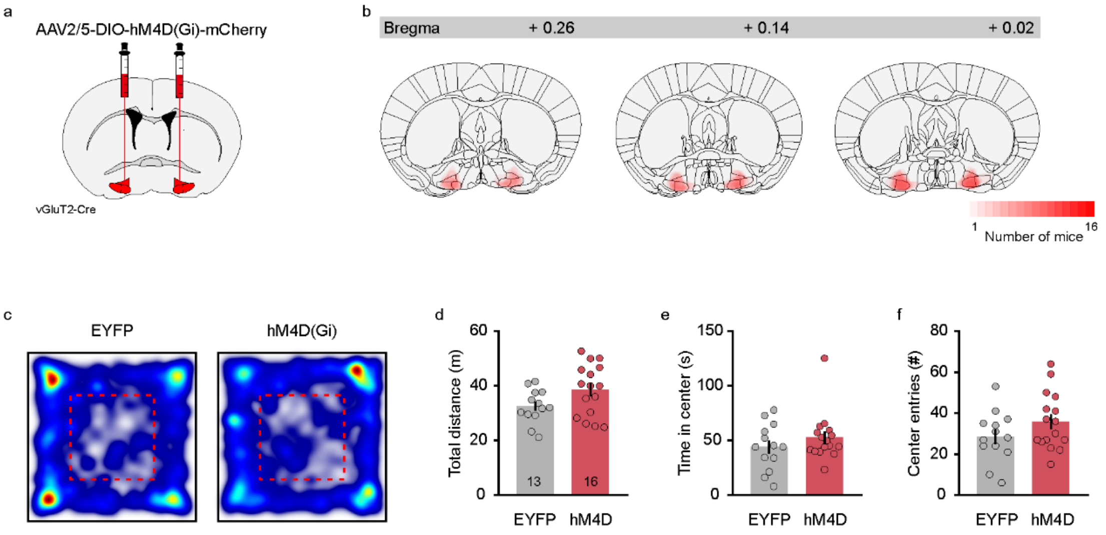

Supplementary Fig. 3 Chemogenetic inhibition of BF vGluT2 neurons does not alter locomotion or general anxiety-like behavior.

(**a**) Schematic illustration of hM4D(Gi)-mCherry virus injection in the bilateral BF of vGluT2-Cre mice.

(**b**) Overlay of hM4D(Gi)-mCherry expression in the BF of 16 vGluT2-Cre mice. Red, viral targeting.

(**c**) Representative heatmaps showing the movement traces of an EYFP-expressing mouse (left) and an hM4D-expressing mouse (right) in an open field test.

(**d-f**) Quantification of the total distance (**d**) ($P = 0.0594$), time in center (**e**) ($P = 0.2985$) and center entries (**f**) ($P = 0.1503$) of EYFP and hM4D mice in an open field test. n = 13 for EYFP group, n = 16 for hM4D group. There is no significant difference between two groups, two-sided unpaired *t* test.

Error bars indicate mean ± SEM. Source data are provided as a Source Data file.

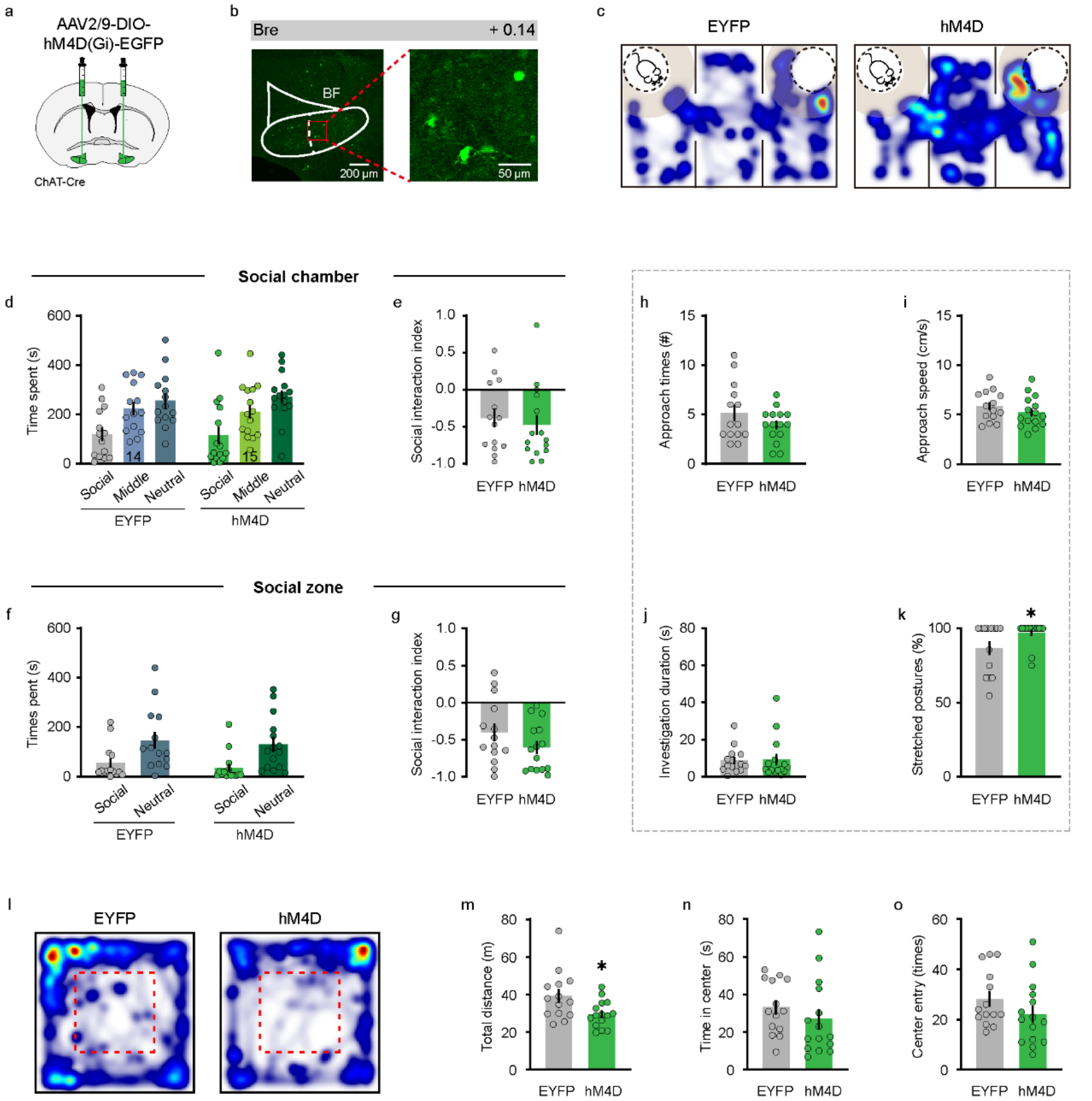

Supplementary Fig. 4 Chemogenetic inhibition of BF ChAT neurons does not alter social fear expression.

(**a**) Schematic illustration of hM4D(Gi)-EGFP virus injection in the bilateral BF of ChAT-Cre mice.

(**b**) Representative image showing the expression of hM4D(Gi)-EGFP in the BF.

(**c**) Representative heatmaps showing movement traces of an EYFP-expressing mouse (left) and an hM4D(Gi)-expressing mouse (right) in a three-chamber social interaction test.

(**d**) Quantification of time spent by EYFP- and hM4D-expressing mice in each chamber. n = 14 for EYFP group; n = 15 for hM4D group. $F_{interaction}(2, 54) = 0.08$, $P = 0.9192$;

$F_{chamber}(2, 54) = 8.97$, $P = 0.0004$; $F_{group}(1, 27) = 0.34$, $P = 0.5671$; two-way ANOVA followed by Bonferroni's multiple comparisons.

(**e**) Social interaction index was unchanged in hM4D(Gi) mice. n = 14 for EYFP group; n = 15 for hM4D group. *P* = 0.6060, two-sided unpaired *t* test.

(**f**) The same as (**d**) but for the 8 cm social zone. $F_{interaction}(1, 27) = 0.01$, $P = 0.9437$; $F_{zone}(1, 27) = 15.96$, $P = 0.0004$; $F_{group}(1, 27) = 0.49$, $P = 0.4882$; two-way ANOVA followed by Bonferroni's multiple comparisons.

(**g**) The same as (**e**) but for the 8 cm social zone. n = 14 for EYFP group; n = 15 for hM4D group. *P* = 0.1612, two-sided unpaired *t* test.

(**h-k**) Comparison of the approach times (**h**) (*P* = 0.1823), approach speed (**i**) (*P* = 0.2957), investigation duration (**j**) (*P* = 0.8953) and percentage of stretched postures (**k**) (*P* = 0.0468). Two-sided unpaired *t* test.

(**l**) Representative heatmaps showing the movement traces of an EYFP-expressing mouse (left) and an hM4D-expressing mouse (right) in an open field test.

(**m-o**) Quantification of the total distance (**m**) (*P* = 0.0190), time in center (**n**) (*P* = 0.3476) and center entries (**o**) (*P* = 0.1878) of EYFP and hM4D mice in an open field test. = 14 for EYFP group; n = 15 for hM4D group. Two-sided unpaired *t* test.

Error bars indicate mean ± SEM. $^*P < 0.05$. Source data are provided as a Source Data file.

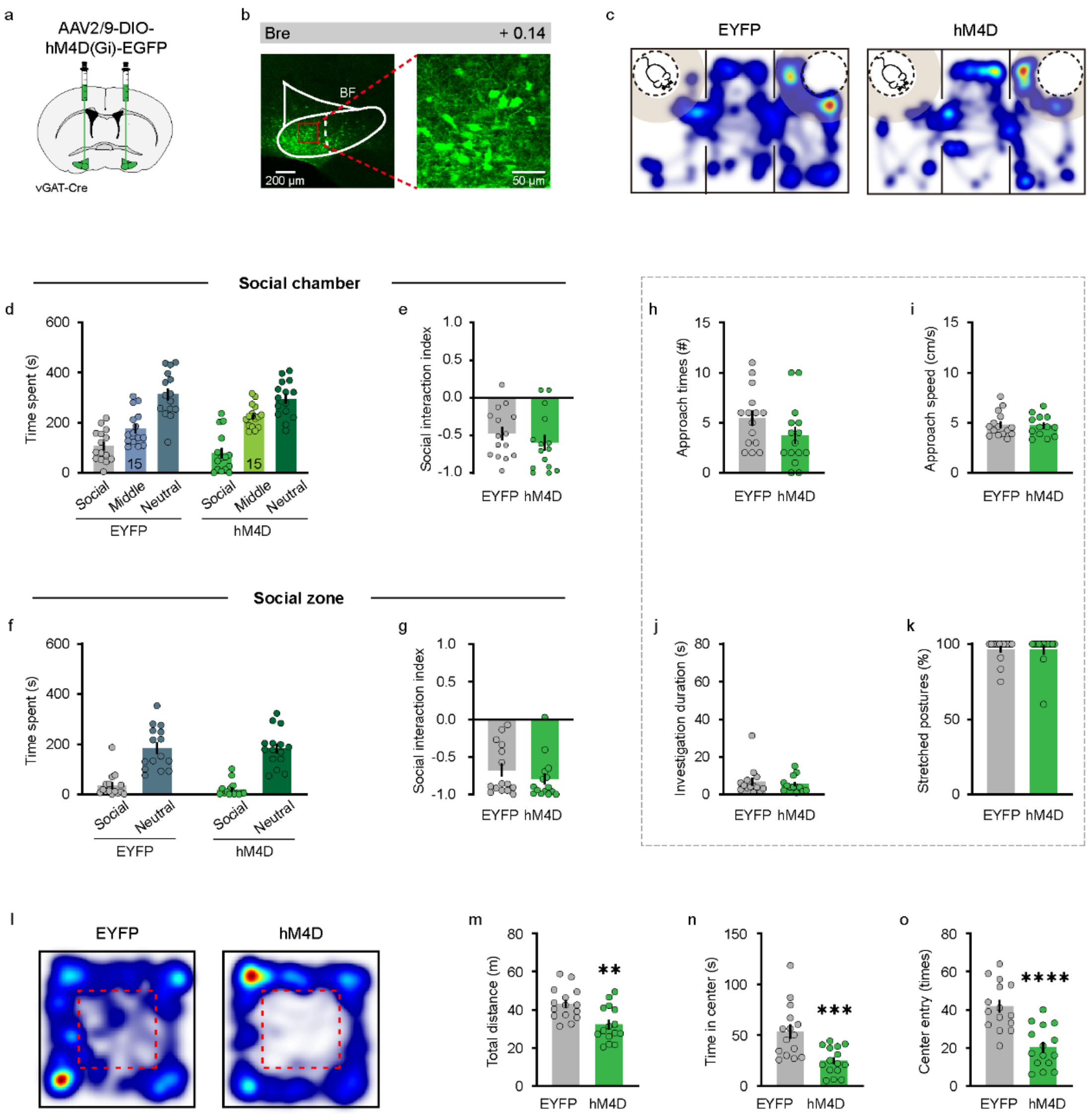

Supplementary Fig. 5 Chemogenetic inhibition of BF vGAT neurons does not alter social fear expression.

(**a**) Schematic illustration of hM4D(Gi)-EGFP virus injection in the bilateral BF of vGAT-Cre mice.

(**b**) Representative image showing the expression of hM4D(Gi)-EGFP in the BF.

(**c**) Representative heatmaps showing movement traces of an EYFP-expressing mouse (left) and an hM4D(Gi)-expressing mouse (right) in a three-chamber social interaction test.

(**d**) Quantification of time spent by EYFP- and hM4D-expressing mice in each chamber. n = 15 for EYFP group; n = 15 for hM4D group. $F_{interaction}(2, 56) = 1.75$, $P = 0.1836$;

$F_{chamber}(2, 56) = 42.78$, $P < 0.0001$; $F_{group}(1, 28) = 0.84$, $P = 0.3679$; two-way ANOVA followed by Bonferroni's multiple comparisons.

(**e**) Social interaction index was unchanged in hM4D(Gi) mice. n = 15 for each group. *P* = 0.3702, two-sided unpaired *t* test.

(**f**) The same as (**d**) but for the 8 cm social zone. $F_{interaction}(1, 28) = 0.18$, $P = 0.6754$; $F_{zone}(1, 28) = 76.85$, $P < 0.0001$; $F_{group}(1, 28) = 0.28$, $P = 0.5990$; two-way ANOVA followed by Bonferroni's multiple comparisons.

(**g**) The same as (**e**) but for the 8 cm social zone. n = 15 for each group. *P* = 0.2927, two-sided unpaired *t* test.

(**h-k**) Comparison of the approach times (**h**) (*P* = 0.1253), approach speed (**i**) (*P* = 0.8355), investigation duration (**j**) (*P* = 0.6821) and percentage of stretched postures (**k**) (*P* = 0.8984) of EYFP and hM4D mice upon approaching a stimulus mouse. n = 15 for each group. Two-sided unpaired *t* test.

(**l**) Representative heatmaps showing the movement traces of an EYFP-expressing mouse (left) and an hM4D-expressing mouse (right) in an open field test.

(**m-o**) Quantification of the total distance (**m**) (*P* = 0.0025), time in center (**n**) (*P* = 0.0008) and center entries (**o**) (*P* < 0.0001) of EYFP and hM4D mice in an open field test. n = 15 for each group. Two-sided unpaired *t* test.

Error bars indicate mean ± SEM. ***P* < 0.01, ****P* < 0.001, *****P* < 0.0001. Source data are provided as a Source Data file.

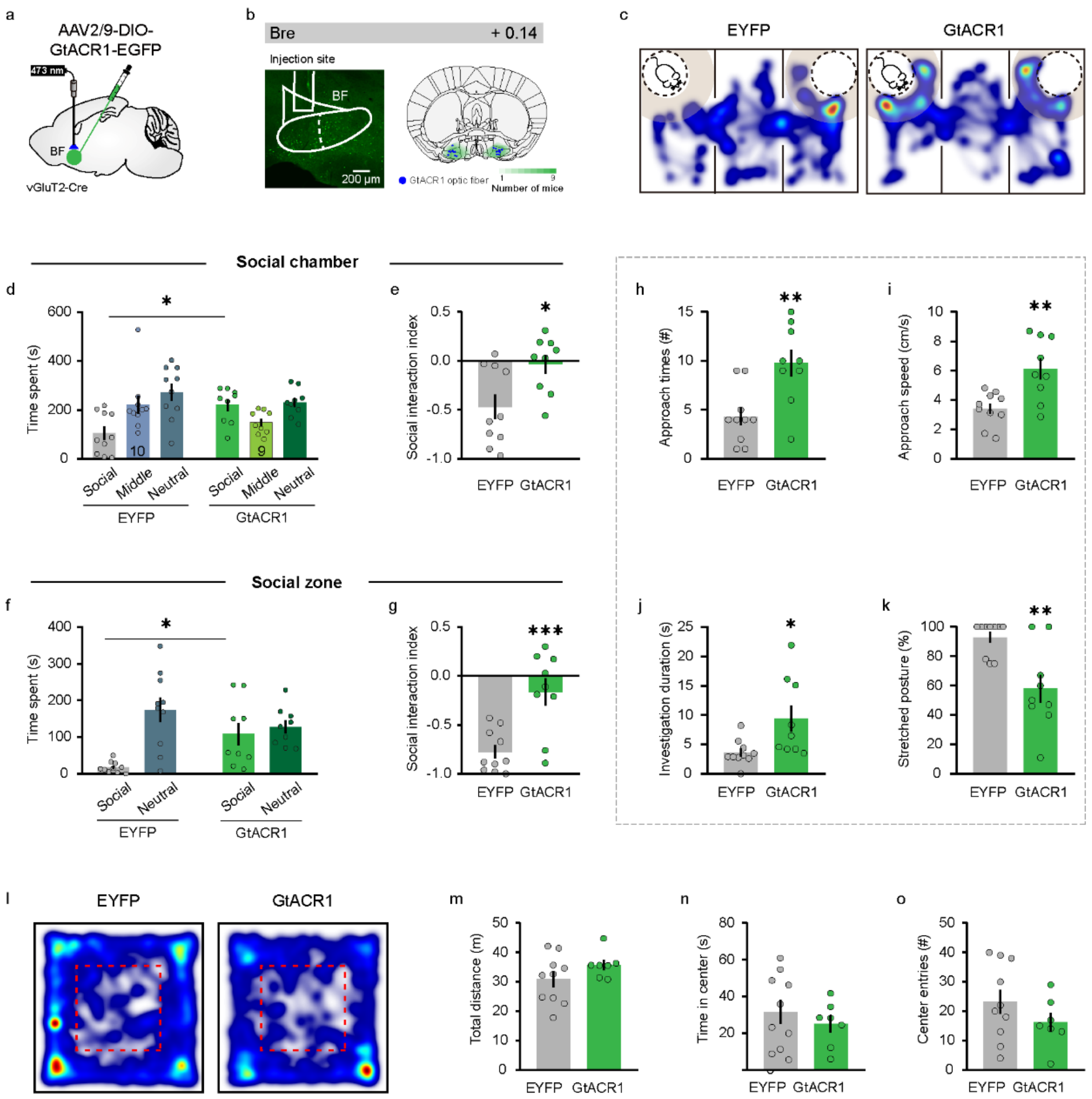

Supplementary Fig. 6 Optogenetic inhibition of BF vGluT2 neurons reduces social fear in conditioned mice.

(**a**) Schematic illustration of optogenetic inhibition of BF vGluT2 neurons.

(**b**) Representative image showing GtACR1-EGFP expression in the BF (left). Overlay of GtACR1-EGFP expression and placement of optical fibers in the BF of 9 vGluT2-Cre mice (right). Green, viral targeting. Blue dots: tips of optical fibers.

(**c**) Representative heatmaps showing the movement traces.

(**d**) Quantification of time spent by EYFP- and GtACR1-expressing mice in each chamber. n = 10 for EYFP group; n = 9 for GtACR1 group. $F_{interaction}(2, 34) = 4.32$, $P = 0.0213$; $F_{chamber}(2, 34) = 3.63$, $P = 0.0372$; $F_{group}(1, 17) = 0.042$, $P = 0.8408$; time spent in social chamber (EYFP vs. GtACR1): $P = 0.0163$, two-way ANOVA followed by

Bonferroni's multiple comparisons.

(**e**) Quantification of social interaction index. n = 10 for EYFP group; n = 9 for GtACR1 group. *P* = 0.0149, two-sided unpaired *t* test.

(**f**) The same as (**d**) but for the 8 cm social zone. $F_{interaction}(1, 17) = 8.04$, *P* = 0.0114; $F_{zone}(1, 17) = 13.16$, *P* = 0.0021; $F_{group}(1, 17) = 0.38$, *P* = 0.3716; time spent in social zone (EYFP vs. GtACR1): *P* = 0.0248, two-way ANOVA followed by Bonferroni's multiple comparisons.

(**g**) The same as (**e**) but for the 8 cm social zone. n = 10 for EYFP group; n = 9 for GtACR1 group. *P* = 0.0008, two-sided unpaired *t* test.

(**h-k**) Quantification of the approach times (**h**) (*P* = 0.0031), approach speed (**i**) (*P* = 0.0028), investigation duration (**j**) (*P* = 0.0186) and proportion of stretched postures (**k**) (*P* = 0.0023). Two-sided unpaired *t* test.

(**L**) Representative heatmaps show the movement traces in an open field test.

(**m-o**) Quantification of the total distance (**m**) (*P* = 0.1718), time in center (**n**) (*P* = 0.4684) and center entries (**o**) (*P* = 0.2304). n = 10 for EYFP group; n = 7 for GtACR1 group. Two-sided unpaired *t* test.

Error bars indicate mean ± SEM. **P* < 0.05, ***P* < 0.01, ****P* < 0.001. Source data are provided as a Source Data file.

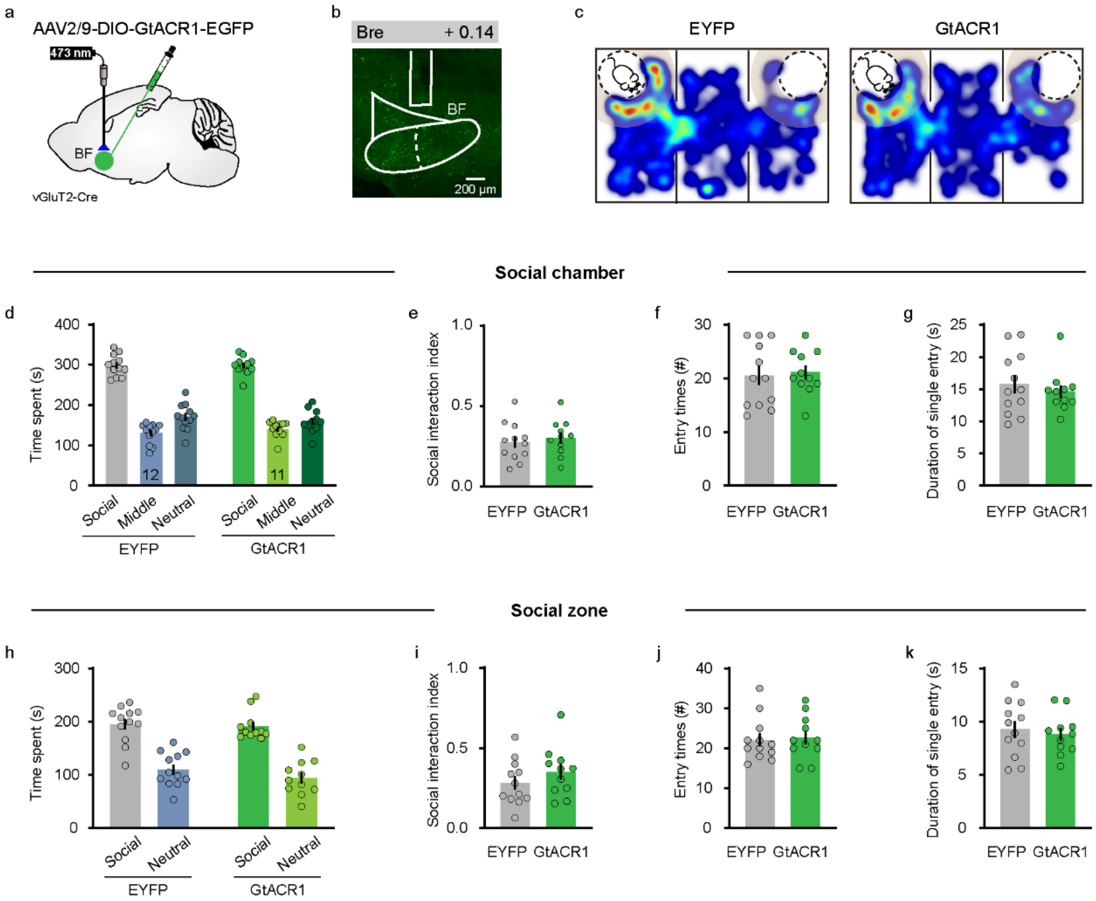

Supplementary Fig. 7 Optogenetic inhibition of BF vGluT2 neurons does not affect social interaction in unconditioned mice.

(**a**) Schematic illustration of GtACR1-EGFP virus injection and optical fibers implantation in the bilateral BF of vGluT2-Cre mice.

(**b**) Representative image showing GtACR1-EGFP expression and placement of optical fiber in the BF.

(**c**) Representative heatmaps showing the movement traces of an EYFP-expressing mouse (left) and a GtACR1-expressing mouse (right) in a three-chamber social interaction test.

(**d**) Quantification of time spent by EYFP- and GtACR1-expressing mice in each chamber. n = 12 for EYFP group; n = 11 for GtACR1 group. $F_{interaction}(2, 42) = 0.60$, *P* = 0.5531; $F_{chamber}(2, 42) = 163.80$, *P* < 0.0001; $F_{group}(1, 21) = 0.44$, *P* = 0.5120; two-way ANOVA followed by Bonferroni's multiple comparisons.

(**e-g**) Quantification of the social interaction index (**e**) (*P* = 0.5753), entry times (**f**) (*P* = 0.7863) and duration of single entry (**g**) (*P* = 0.5294) of EYFP and GtACR1 mice. n = 12 for EYFP group; n = 11 for GtACR1 group. Two-sided unpaired t test.

(**h-k**) The same as (**d-g**) but for the 8 cm social zone. $F_{interaction}(1, 21) = 0.57$, $P = 0.4589$; $F_{zone}(1, 21) = 123.90$, $P < 0.0001$; $F_{group}(1, 21) = 0.93$, $P = 0.3459$; two-way ANOVA followed by Bonferroni's multiple comparisons (**h**), or two-sided unpaired *t* test (**i-k**). (**i**) *P* = 0.2898, (**j**) *P* = 0.8123, (**k**) *P* = 0.6428. n = 12 for EYFP group; n = 11 for GtACR1 group.

Error bars indicate mean ± SEM. Source data are provided as a Source Data file.

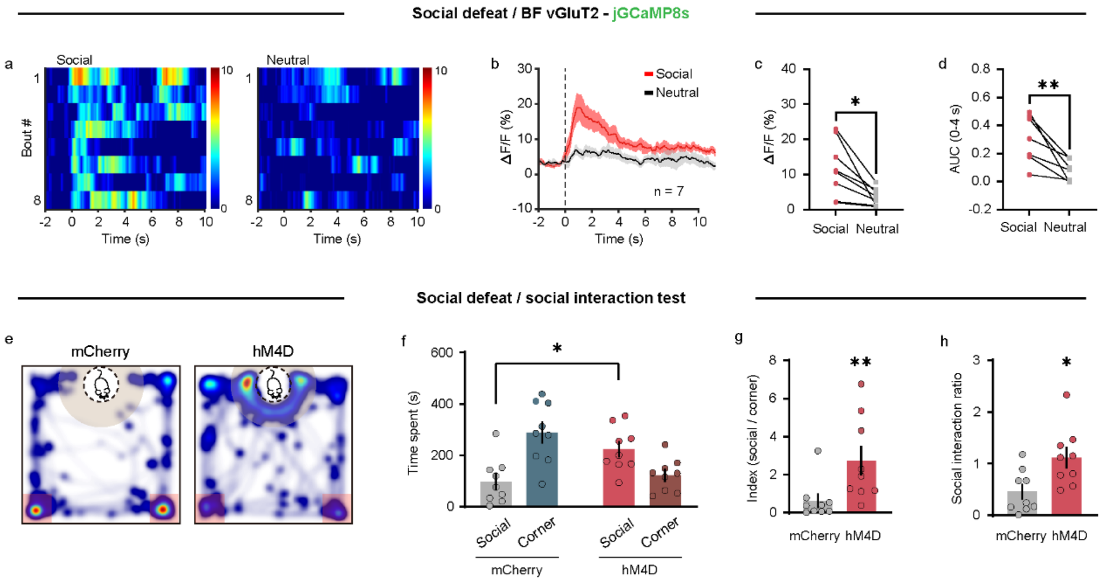

Supplementary Fig. 8 The role of BF vGluT2 neurons in social fear induced by sub-chronic social defeat.

(**a**) Heatmap of BF vGluT2 Ca^{2+} signals in social preference-avoidance test after sub-chronic social defeat. Ca^{2+} signals were aligned to the onset of individual interactions with a CD1 social stimulus (left) or an empty neutral cage (right). Each row represents one interaction bout, and the color scale at the right indicates ΔF/F.

(**b**) The peri-event plot of the mean Ca^{2+} transient during social or neutral interactions for the entire test group (n = 7 mice). The thick line indicates the mean, and the shaded area indicates SEM.

(**c**) Statistical comparison of peak fluorescence signals between social and neutral interactions. n = 7 mice. $P = 0.0120$, two-sided paired t test.

(**d**) Statistical comparison of area under curve (0-4 s) of BF vGluT2 fluorescence signals between social and neutral interactions. n = 7 mice. $P = 0.0093$, two-sided paired t test.

(**e**) Representative heatmaps showing the movement traces of an mCherry-expressing mouse (left) and an hM4D-expressing mouse (right) in a social preference-avoidance

test.

(**f**) Quantification of time spent by mCherry- and hM4D-expressing mice in social and corner zones. n = 9 for mCherry group; n = 9 for hM4D group. $F_{interaction}(1, 16) = 13.27$, $P = 0.0022$; $F_{zone}(1, 16) = 1.22$, $P = 0.2854$; $F_{group}(1, 16) = 1.87$, $P = 0.1908$; time spent in social zone (mCherry vs. hM4D): $P = 0.0123$, two-way ANOVA followed by Bonferroni's multiple comparisons.

(**g**) Quantification of social interaction index [(time spent in social zone) / (time spent in corner zones)] between mCherry and hM4D mice. n = 9 mice for each group. $P = 0.0040$, two-sided unpaired *t* test.

(**h**) Quantification of social interaction ratio (time spent in social zone with social stimulus divided by without social stimulus) between mCherry and hM4D mice. n = 9 mice for each group. $P = 0.0119$, two-sided unpaired *t* test.

Error bars indicate mean ± SEM. *$P < 0.05$, **$P < 0.01$. Source data are provided as a Source Data file.

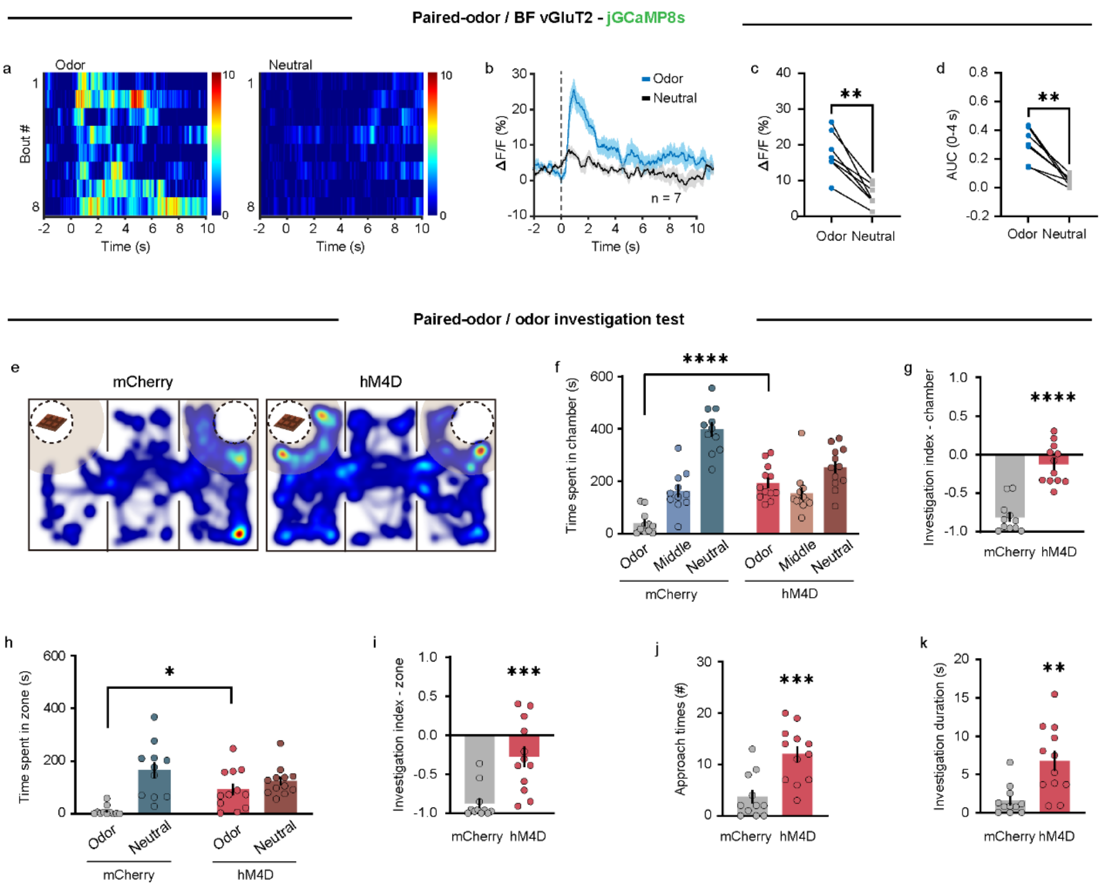

Supplementary Fig. 9 The role of BF vGluT2 neurons in fear expression induced by odor-foot shock association.

(**a**) Heatmap of BF vGluT2 Ca^{2+} signals in three-chamber test after odor-paired fear learning. Ca^{2+} signals were aligned to the onset of individual investigations with a paired-odor (left) or an empty neutral cage (right). Each row represents one investigation bout, and the color scale at the right indicates ΔF/F.

(**b**) The peri-event plot of the mean Ca^{2+} transient during odor or neutral investigations for the entire test group (n = 7 mice). The thick line indicates mean, and the shaded area indicates SEM.

(**c, d**) Statistical comparison of peak (**c**) (P = 0.0011) and area under curve (**d**) (P = 0.0013) of fluorescence signals between odor and neutral investigations. n = 7 mice., two-sided paired t test.

(**e**) Representative heatmaps showing the movement traces of an mCherry-expressing mouse (left) and an hM4D-expressing mouse (right).

(**f**) Quantification of time spent by mCherry- and hM4D-expressing mice in each chamber. n = 11 for mCherry group; n = 12 for hM4D group. $F_{interaction}(2, 42) = 15.65$, $P < 0.0001$; $F_{chamber}(2, 42) = 34.09$, $P < 0.0001$; $F_{group}(1, 21) = 1.10$, $P = 0.3071$; time spent in odor chamber (mCherry vs. hM4D): $P < 0.0001$, two-way ANOVA followed by Bonferroni's multiple comparisons.

(**g**) Quantification of investigation index. $P < 0.0001$, two-sided unpaired t test.

(**h**) The same as (**f**) but for the 8 cm odor interaction zone. $F_{interaction}(1, 21) = 8.85$, $P = 0.0072$; $F_{zone}(1, 21) = 20.22$, $P = 0.0002$; $F_{group}(1, 21) = 0.96$, $P = 0.3389$; time spent in odor zone (mCherry vs. hM4D): $P = 0.0157$, two-way ANOVA followed by Bonferroni's multiple comparisons.

(**i-k**) Quantification of odor investigation index (**i**) ($P = 0.0007$), approach times (**j**) ($P = 0.0006$) and investigation duration (**k**) ($P = 0.0020$) of mCherry and hM4D mice. n = 11 for mCherry group; n = 12 for hM4D group. Two-sided unpaired t test.

Error bars indicate mean ± SEM. $^*P < 0.05$, $^{**}P < 0.01$, $^{***}P < 0.001$, $^{****}P < 0.0001$.

Source data are provided as a Source Data file.

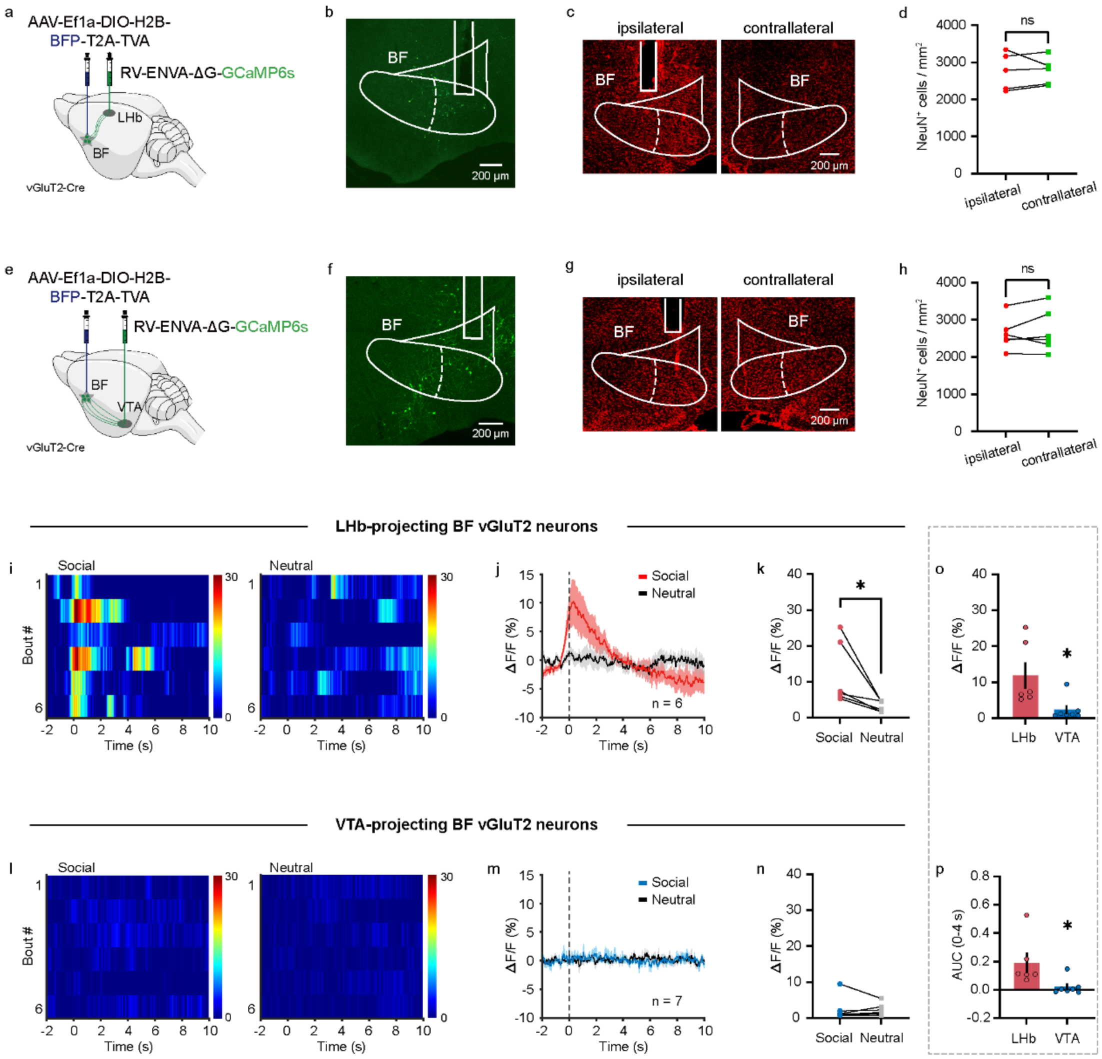

Supplementary Fig. 10 The LHb-projecting but not VTA-projecting BF vGluT2 neurons respond to social fear expression.

(**a, b**) Schematic illustration (**a**) and representative image (**b**) of RV-GCaMP6s expression in LHb-projecting BF vGluT2 neurons.

(**c**) Nissl staining for BF of the recording side (left) and the contralateral side (right).

(**d**) Number of neurons in BF between the recording side and the contralateral side. n = 5 mice. *P* = 0.9788, two-sided paired *t* test.

(**e-h**) The same as (**a-d**) but for VTA-projecting BF vGluT2 neurons. (**h**) n = 6 mice. *P* = 0.4888, two-sided paired *t* test.

(**i**) Heatmap of Ca^{2+} signals of a mouse with GCaMP6s expression in LHb-projecting

BF vGluT2 neurons. The Ca^{2+} signals were aligned to the onset of individual social interaction with a social stimulus (left) or a neutral cage (right). Each row represents one bout, and the color scale at the right indicates ΔF/F.

(**j**) The peri-event plot of the mean Ca^{2+} transient during social or neutral interactions for the entire test group (n = 6 mice). The thick line indicates the mean, and the shaded area indicates SEM.

(**k**) Statistical comparison of peak fluorescence signals between social and neutral interactions. n = 6 mice. $P = 0.0429$, two-sided paired *t* test.

(**l-n**) The same as (**i-k**) but for recordings made in the VTA (n =7 mice). (**n**) $P = 0.8275$, two-sided paired *t* test.

(**o**) Statistical comparison of peak fluorescence signals during social fear recorded in the LHb and in the VTA. n = 6 mice for LHb group; n = 7 mice for VTA group. $P = 0.0210$, two-sided unpaired *t* test.

(**p**) Statistical comparison of area under curve (0-4 s) of fluorescence signals during social fear recorded in the LHb and in the VTA. n = 6 mice for LHb group; n = 7 mice for VTA group. $P = 0.0305$, two-sided unpaired *t* test.

Error bars indicate mean ± SEM. ns, no significant difference, $^{*}P < 0.05$. Source data are provided as a Source Data file.

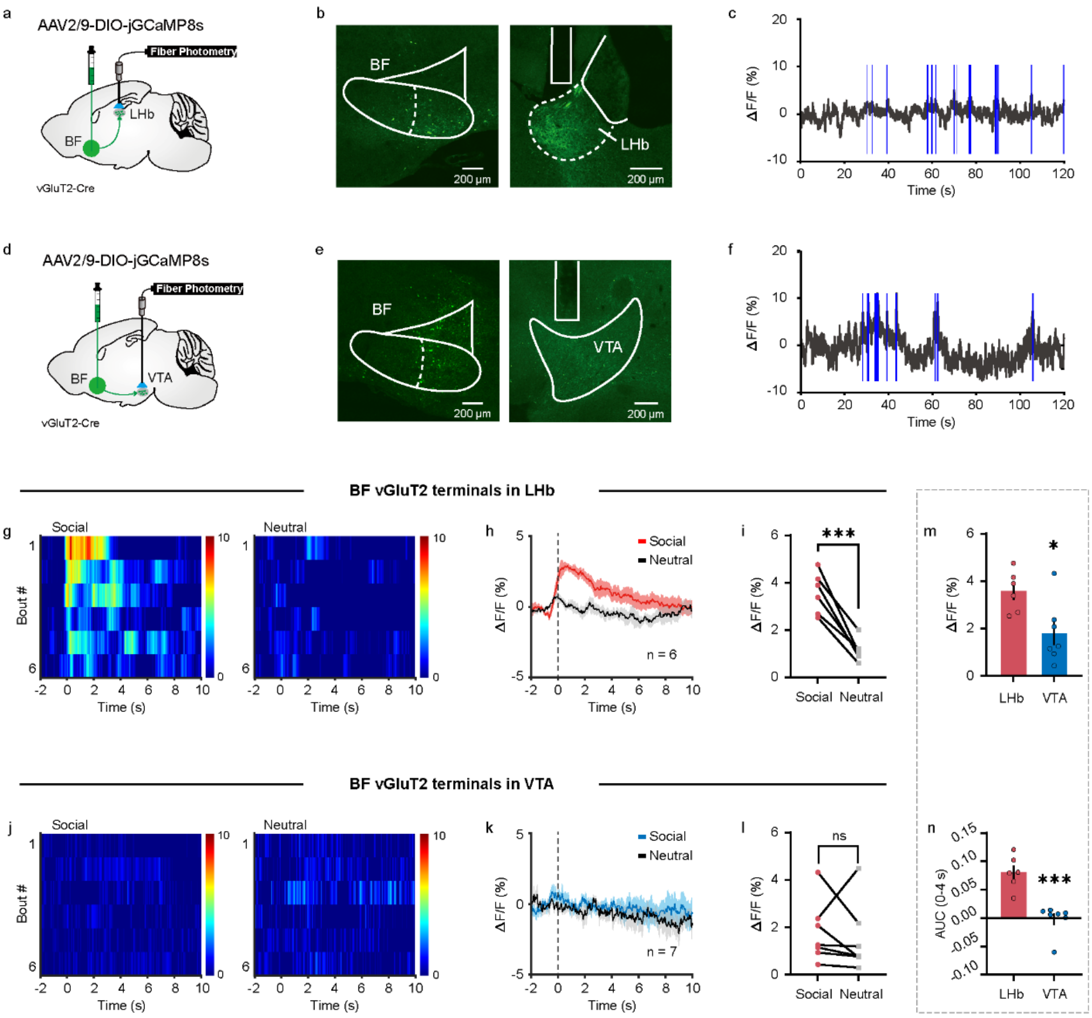

Supplementary Fig. 11 Social fear expression activates axon terminals in the LHb but not the VTA of BF vGluT2 neurons.

(**a**) Schematic illustration of AAV-hSyn-DIO-jGCaMP8s virus injection in the BF and fiber photometry recording of the LHb-projecting BF vGluT2 axon terminals in a three-chamber social interaction test.

(**b**) Representative images showing virus expression in the BF (left) and axon terminals expression and placement of optical fiber in the LHb (right).

(**c**) A representative trace of fluorescence signals recorded from axon terminals of BF vGluT2 neurons in the LHb during habituation session. Blue lines indicate periods with spontaneous calcium events.

(**d-f**) The same as the (**a-c**) but for VTA recording.

(**g**) Heatmap of Ca^{2+} signals from axon terminals in the LHb in a conditioned mouse. Ca^{2+} signals were aligned to the onset of individual interactions with a social stimulus (left) or an empty neutral cage (right). Each row represents one interaction bout, and the color scale at the right indicates $\Delta F/F$.

(**h**) The peri-event plot of the mean Ca^{2+} transient during social or neutral interactions for the entire test group (n = 6 mice). The thick line indicates the mean, and the shaded area indicates SEM.

(**i**) Statistical comparison of peak fluorescence signals between social and neutral interactions. n = 6 mice. $P = 0.007$, two-sided paired t test.

(**j-l**) The same as (**g-i**) but for recordings made in the VTA (n =7 mice). (**l**) $P = 0.5740$, two-sided paired t test.

(**m**) Statistical comparison of peak fluorescence signals recorded in the LHb and in the VTA. n = 6 mice for LHb group; n = 7 mice for VTA group. $P = 0.0159$, two-sided unpaired t test.

(**n**) Statistical comparison of area under curve (0-4 s) of fluorescence signals recorded in the LHb and in the VTA. n = 6 mice for LHb group; n = 7 mice for VTA group. $P = 0.0003$, two-sided unpaired t test.

Error bars indicate mean ± SEM. ns, no significant difference, $^{*}P < 0.05$, $^{***}P < 0.001$.

Source data are provided as a Source Data file.

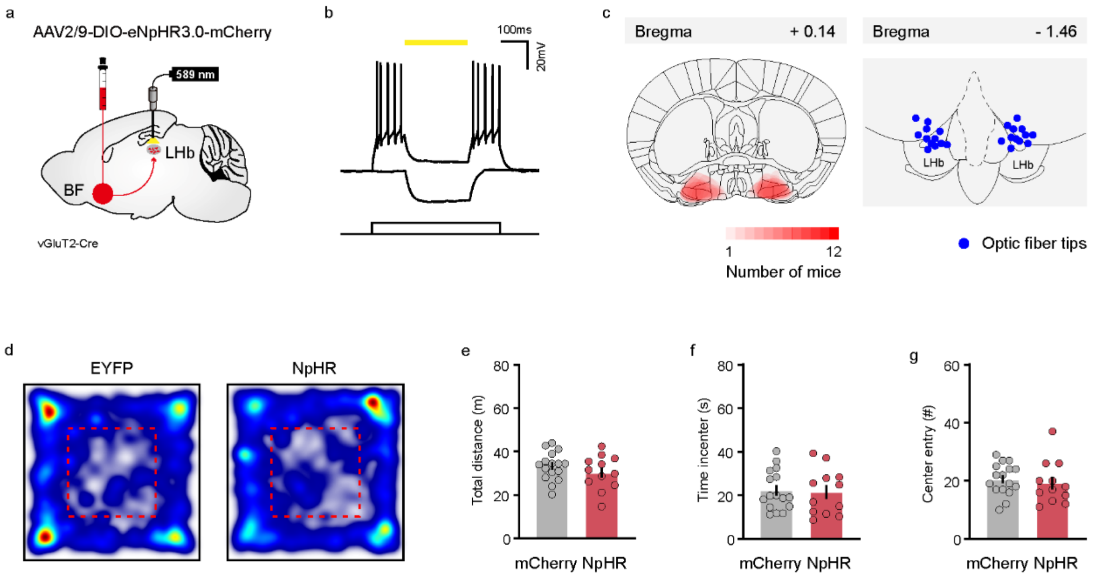

Supplementary Fig. 12 Optogenetic inhibition of BF-LHb glutamatergic projections does not alter locomotion or general anxiety-like behavior.

(**a**) Schematic illustration of eNpHR3.0-mCherry virus injection in the BF of vGluT2-Cre mice and optical fiber implantation in the LHb.

(**b**) Example traces showing yellow light induced a hyperpolarization and inhibited the action potential evoked by depolarizing current in an NpHR-expressing BF vGluT2 neuron.

(**c**) Overlay of NpHR-mCherry expression in the BF (left), and placement of optical fibers in the BF of 12 vGluT2-Cre mice (right). Red, viral targeting. Blue dots: tips of optical fibers.

(**d**) Representative heatmaps showing the movement traces of an mCherry-expressing mouse (left) and an NpHR-expressing mouse (right) in an open field test.

(**e-g**) Quantification of the total distance (**e**) ($P = 0.2875$), time in center (**f**) ($P = 0.8488$) and center entries (**g**) ($P = 0.6102$) of mCherry and NpHR mice in open field tests. n = 16 for mCherry group, n = 12 for hM4D group. There is no significant difference between two groups, two-sided unpaired t test.

Error bars indicate mean ± SEM. Source data are provided as a Source Data file.

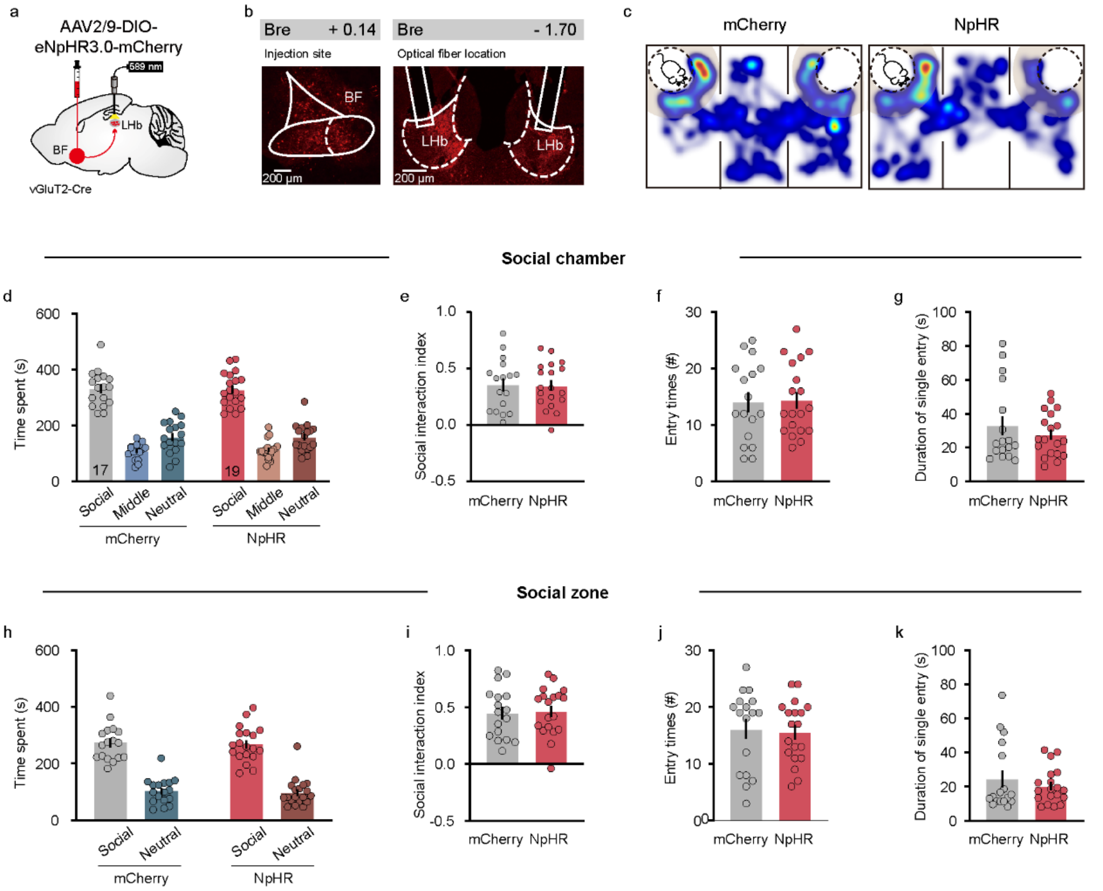

Supplementary Fig. 13 Optogenetic inhibition of BF-LHb glutamatergic projections does not affect social interaction in unconditioned mice.

(**a**) Schematic illustration of eNpHR3.0-mCherry virus injection and optical fibers implantation in the bilateral LHb of vGluT2-Cre mice.

(**b**) Representative image showing eNpHR3.0-mCherry expression and placement of optical fiber in the LHb.

(**c**) Representative heatmaps showing the movement traces of an mCherry-expressing mouse (left) and an NpHR-expressing mouse (right) in a three-chamber social interaction test.

(**d**) Quantification of time spent by mCherry- and NpHR-expressing mice in each chamber. n = 17 for mCherry group; n = 19 for NpHR group. $F_{interaction}(2, 68) = 0.03$, $P = 0.9656$; $F_{chamber}(2, 68) = 127.60$, $P < 0.0001$; $F_{group}(1, 34) = 0.01$, $P = 0.9222$; two-way ANOVA followed by Bonferroni's multiple comparisons.

(**e-g**) Quantification of the social interaction index (**e**) ($P = 0.9422$), approach times (**f**)

(P = 0.8905) and investigation duration (**g**) (P = 0.4007) of mCherry and NpHR mice. n = 17 for mCherry group; n = 19 for NpHR group. There is no significant difference between two groups, two-sided unpaired t test.

(**h-k**) The same as (**d-g**) but for the 8 cm social zone. n = 17 for mCherry group; n = 19 for NpHR group. (**h**) $F_{interaction}(1, 34) = 0.001$, $P = 0.9735$; $F_{zone}(1, 34) = 116.10$, $P < 0.0001$; $F_{group}(1, 34) = 0.26$, $P = 0.46137$; two-way ANOVA followed by Bonferroni's multiple comparisons. (**i**) P = 0.8269, (**j**) P = 0.7998, (**k**) P = 0.4135; there is no significant difference between two groups, two-sided unpaired t test.

Error bars indicate mean ± SEM. Source data are provided as a Source Data file.

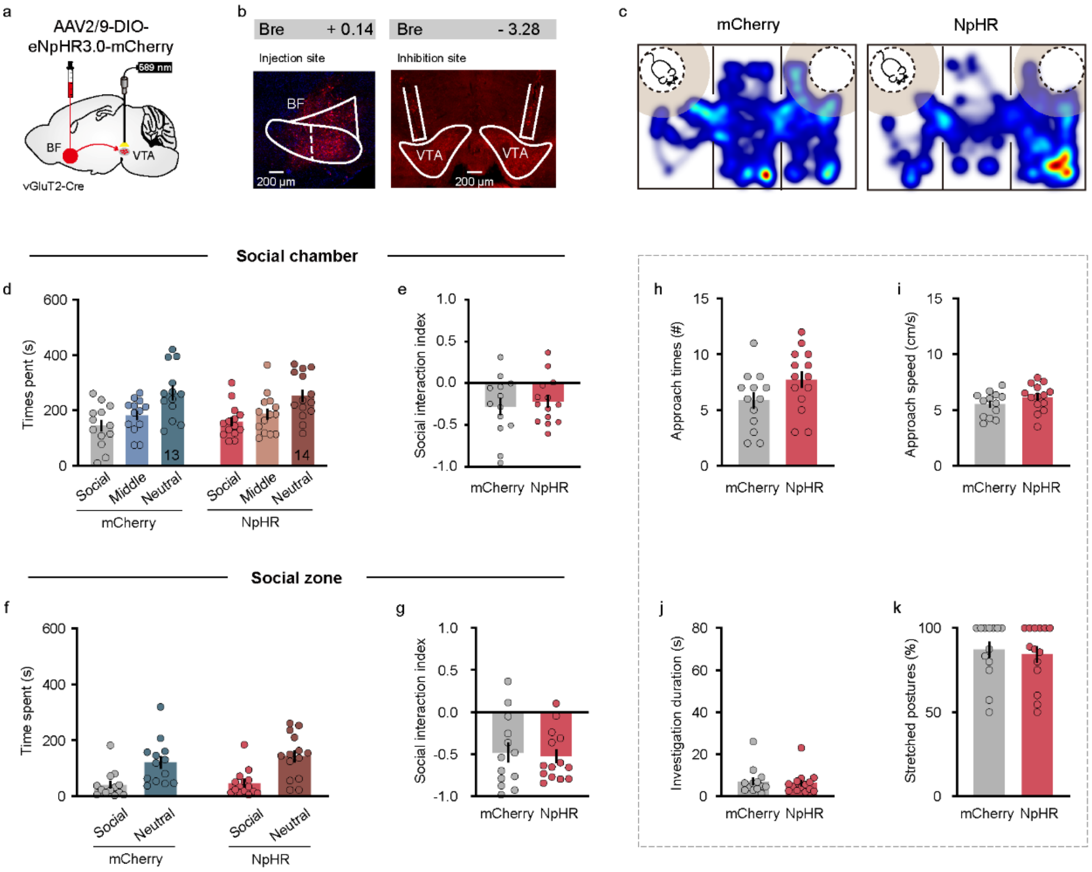

Supplementary Fig. 14 Optogenetic inhibition of BF-VTA glutamatergic projections does not alter social fear in conditioned mice.

(**a**) Schematic illustration of eNpHR3.0-mCherry virus injection in the BF of vGluT2-Cre mice and optical fiber implantation in the VTA.

(**b**) Representative images showing NpHR expression in the BF, and placement of optical fibers in the VTA with NpHR-expressing axon terminals.

(**c**) Representative heatmaps showing the movement traces of an mCherry-expressing mouse (left) and an NpHR-expressing mouse (right) in a three-chamber social interaction test.

(**d**) Quantification of time spent by mCherry- and NpHR-expressing mice in each chamber. n = 13 for mCherry group; n = 14 for NpHR group. $F_{interaction}(2, 50) = 0.13$, $P = 0.8757$; $F_{chamber}(2, 50) = 9.44$, $P = 0.0003$; $F_{group}(1, 25) = 1.08$, $P = 0.3091$; two-way ANOVA followed by Bonferroni's multiple comparisons.

(**e**) Social interaction index was not changed in NpHR mice compared to that in the mCherry group. n = 13 for mCherry group; n = 14 for NpHR group. *P* = 0.6038, two-sided unpaired *t* test.

(**f**) The same as (**d**) but for the 8 cm social zone. n = 13 for mCherry group; n = 14 for NpHR group. $F_{interaction}(1, 25) = 0.23$, $P = 0.6370$; $F_{zone}(1, 25) = 26.03$, $P < 0.0001$; $F_{group}(1, 25) = 0.53$, $P = 0.4739$; two-way ANOVA followed by Bonferroni's multiple comparisons.

(**g**) The same as (**e**) but for the 8 cm social zone. n = 13 for mCherry group; n = 14 for NpHR group. *P* = 0.7591, two-sided unpaired *t* test.

(**h-k**) Quantification of the approach times (**h**) (*P* = 0.0832), approach speed (**i**) (*P* = 0.1742), investigation duration (**j**) (*P* = 0.7494) and percentage of stretched postures (**k**) (*P* = 0.6918) of mCherry and NpHR mice. n = 13 for mCherry group; n = 14 for NpHR group. There is no significant difference between two groups, two-sided unpaired *t* test. Error bars indicate mean ± SEM. Source data are provided as a Source Data file.

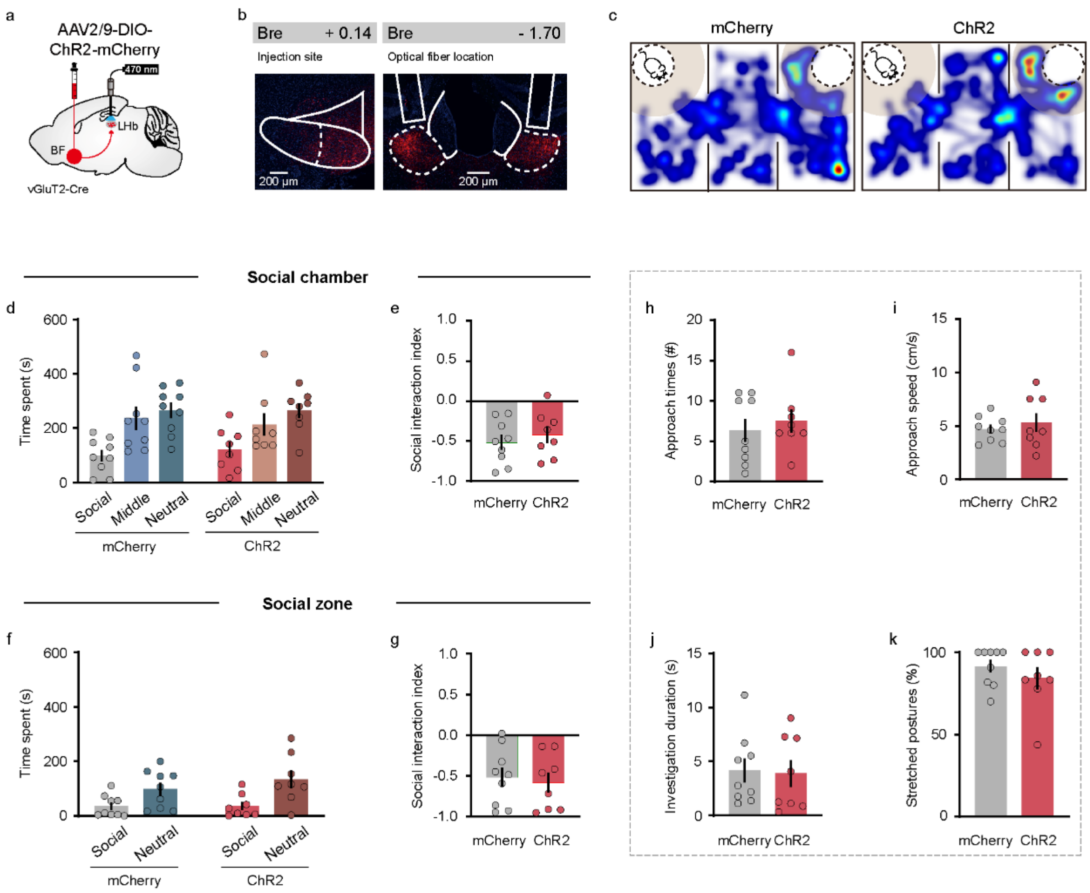

Supplementary Fig. 15 Optogenetic activation of BF-LHb glutamatergic projections does not alter social fear in conditioned mice.

(**a**) Schematic illustration of ChR2-mCherry virus injection in the BF of vGluT2-Cre mice and optical fiber implantation in the LHb.

(**b**) Representative images showing ChR2 expression in the BF, and placement of optical fibers in the LHb with ChR2-expressing axon terminals.

(**c**) Representative heatmaps showing the movement traces of an mCherry-expressing mouse (left) and an ChR2-expressing mouse (right) in a three-chamber social interaction test.

(**d**) Quantification of time spent by mCherry- and ChR2-expressing mice in each chamber. n = 9 for mCherry group; n = 8 for ChR2 group. $F_{interaction}(2, 30) = 0.17$, P = 0.8419; $F_{chamber}(2, 30) = 8.14$, P = 0.0015; $F_{group}(1, 15) = 1.77$, P = 0.2039; two-way ANOVA followed by Bonferroni's multiple comparisons.

(**e**) Social interaction index was not changed in ChR2 mice compared to that in the mCherry group. n = 9 for mCherry group; n = 8 for ChR2 group. *P* = 0.4826, two-sided unpaired *t* test.

(**f**) The same as (**d**) but for the 8 cm social zone. n = 9 for mCherry group; n = 8 for ChR2 group. $F_{interaction}(1, 15) = 0.73$, *P* = 0.4072; $F_{zone}(1, 15) = 15.07$, *P* = 0.0015; $F_{group}(1, 15) = 0.65$, *P* = 0.4326; two-way ANOVA followed by Bonferroni's multiple comparisons.

(**g**) The same as (**e**) but for the 8 cm social zone. n = 9 for mCherry group; n = 8 for ChR2 group. *P* = 0.6995, two-sided unpaired *t* test.

(**h-k**) Quantification of the approach times (**h**) (*P* = 0.5637), approach speed (**i**) (*P* = 0.5224), investigation duration (**j**) (*P* = 0.8549) and percentage of stretched postures (**k**) (*P* = 0.3476) of mCherry and ChR2 mice. n = 9 for mCherry group; n = 8 for ChR2 group. There is no significant difference between two groups, two-sided unpaired *t* test.

Error bars indicate mean ± SEM. Source data are provided as a Source Data file.
