# Supplementary material for: The basal forebrain to lateral habenula circuitry mediates social behavioral maladaptation
Source: Nat Commun. 2024 May 13;15:4013. doi: 10.1038/s41467-024-48378-y (PMC11091113; doi:10.1038/s41467-024-48378-y)
Supplement: Supplementary file 3 — Reporting Summary [file 41467_2024_48378_MOESM3_ESM.pdf]

nature portfolio

Corresponding author(s): Han Xu, Jun Wang

Last updated by author(s): Mar 1, 2024

Reporting Summary

Nature Portfolio wishes to improve the reproducibility of the work that we publish. This form provides structure for consistency and transparency in reporting. For further information on Nature Portfolio policies, see our Editorial Policies and the Editorial Policy Checklist.

Statistics

For all statistical analyses, confirm that the following items are present in the figure legend, table legend, main text, or Methods section.

n/a	Confirmed	
☐	☒	The exact sample size (*n*) for each experimental group/condition, given as a discrete number and unit of measurement
☐	☒	A statement on whether measurements were taken from distinct samples or whether the same sample was measured repeatedly
☐	☒	The statistical test(s) used AND whether they are one- or two-sided<br>*Only common tests should be described solely by name; describe more complex techniques in the Methods section.*
☒	☐	A description of all covariates tested
☐	☒	A description of any assumptions or corrections, such as tests of normality and adjustment for multiple comparisons
☐	☒	A full description of the statistical parameters including central tendency (e.g. means) or other basic estimates (e.g. regression coefficient) AND variation (e.g. standard deviation) or associated estimates of uncertainty (e.g. confidence intervals)
☐	☒	For null hypothesis testing, the test statistic (e.g. *F*, *t*, *r*) with confidence intervals, effect sizes, degrees of freedom and *P* value noted<br>*Give P values as exact values whenever suitable.*
☒	☐	For Bayesian analysis, information on the choice of priors and Markov chain Monte Carlo settings
☒	☐	For hierarchical and complex designs, identification of the appropriate level for tests and full reporting of outcomes
☒	☐	Estimates of effect sizes (e.g. Cohen's *d*, Pearson's *r*), indicating how they were calculated

Our web collection on statistics for biologists contains articles on many of the points above.

Software and code

Policy information about availability of computer code

Data collection	Images were obtained using Olympus Fluoview Viewer software (version 4.2a, Olympus, Japan). Electrophysiology data were collected using MultiClamp 700B and Clampex data acquisition software (version 10.7, Molecular Devices, USA). Fiber photometry data were collected using a custom-written script in LabView (ThinkerTech, Nanjing, China). Multichannel electrophysiological signals and animals' behavior were simultaneously recorded using an OmniPlex neural recording data acquisition system and a CinePlex behavioral research system (Plexon Inc., USA). Behavioral data were collected using Ethovision XT video tracking software (version 11.5, Noldus, Netherland).
Data analysis	Image analyses were conducted using ImageJ (version 1.52, NIH, USA). Slice electrophysiology data were analyzed using Clampfit (version 10.7, Molecular Devices, USA). Fiber photometry data were collected using a custom-written script in Matlab (version R2019b, MathWorks, USA, the script was provided by ThinkerTech, Nanjing, China). Spike signals were sorted using Offline Sorter software (Plexon Inc.). Behavioral data were analyzed using Ethovision XT (version 11.5, Noldus, Netherland). Statistical analyses were performed using Graphpad Prism (version 7, GraphPad, USA).

For manuscripts utilizing custom algorithms or software that are central to the research but not yet described in published literature, software must be made available to editors and reviewers. We strongly encourage code deposition in a community repository (e.g. GitHub). See the Nature Portfolio guidelines for submitting code & software for further information.

Data

Policy information about availability of data

All manuscripts must include a data availability statement. This statement should provide the following information, where applicable:

- Accession codes, unique identifiers, or web links for publicly available datasets
- A description of any restrictions on data availability
- For clinical datasets or third party data, please ensure that the statement adheres to our policy

There are no restrictions on data availability in the manuscript. The data generated in this study are provided in the Source Data file. Source data are provided with this paper.

Research involving human participants, their data, or biological material

Policy information about studies with human participants or human data. See also policy information about sex, gender (identity/presentation), and sexual orientation and race, ethnicity and racism.

Reporting on sex and gender	N/A
Reporting on race, ethnicity, or other socially relevant groupings	N/A
Population characteristics	N/A
Recruitment	N/A
Ethics oversight	N/A

Note that full information on the approval of the study protocol must also be provided in the manuscript.

Field-specific reporting

Please select the one below that is the best fit for your research. If you are not sure, read the appropriate sections before making your selection.

☒ Life sciences ☐ Behavioural & social sciences ☐ Ecological, evolutionary & environmental sciences

For a reference copy of the document with all sections, see nature.com/documents/nr-reporting-summary-flat.pdf

Life sciences study design

All studies must disclose on these points even when the disclosure is negative.

Sample size	No statistical methods were used to pre-determine sample sizes, instead sample sizes were based upon previously published variability of assays, including our own published experiments of a similar nature, and sufficient numbers for duplication (PMID: 30898376, 32832654, 33563763).
Data exclusions	Animals were excluded from behavioral assays based on incorrect targeting of virus or fiber implantation.
Replication	All behavioral assays were repeated multiples times with independent mice. Data for fiber photometry and electrophysiology experiments were pooled from at least three individual animals, collected from at least two rounds of experiments, with successful replication of results. Numbers of replicates (n) are indicated in the figure legends.
Randomization	All animals were arbitrarily assigned to control or experimental groups.
Blinding	All investigators were blinded to group allocation during data collection and analysis.

Reporting for specific materials, systems and methods

We require information from authors about some types of materials, experimental systems and methods used in many studies. Here, indicate whether each material, system or method listed is relevant to your study. If you are not sure if a list item applies to your research, read the appropriate section before selecting a response.

Materials & experimental systems

n/a	Involved in the study
☒	☐ Antibodies
☒	☐ Eukaryotic cell lines
☒	☐ Palaeontology and archaeology
☐	☒ Animals and other organisms
☒	☐ Clinical data
☒	☐ Dual use research of concern
☒	☐ Plants

Methods

n/a	Involved in the study
☒	☐ ChIP-seq
☒	☐ Flow cytometry
☒	☐ MRI-based neuroimaging

Animals and other research organisms

Policy information about studies involving animals; ARRIVE guidelines recommended for reporting animal research, and Sex and Gender in Research

Laboratory animals	Adult vGluT2-Cre (Jackson Laboratory Strain 016963), vGAT-Cre (Jackson Laboratory Strain 016962), ChAT-Cre (Jackson Laboratory Strain 006410), aggressive CD1 mice (7-9 months old, retired breeders) and C57BL/6J wild-type mice (2-4 months old) were used in this study. All animals were group housed in a 12 h light/dark cycle with food and water ad libitum except for those allocated to social fear conditioning (SFC) or implanted with chronic microelectrodes as described below. All mice were housed in a stable environment (23-25 °C ambient temperature and 50% humidity).
Wild animals	The study did not involve wild animals.
Reporting on sex	Only male mice were used in this study.
Field-collected samples	This study did not involve samples collected from the field.
Ethics oversight	Animal care and use were under the guidelines approved by the Animal Care and Use Committee of Zhejiang University.

Note that full information on the approval of the study protocol must also be provided in the manuscript.

Plants

Seed stocks	N/A
Novel plant genotypes	N/A
Authentication	N/A
