# Supplementary material for: The basal forebrain to lateral habenula circuitry mediates social behavioral maladaptation
Source: Nat Commun. 2024 May 13;15:4013. doi: 10.1038/s41467-024-48378-y (PMC11091113; doi:10.1038/s41467-024-48378-y)
Supplement: Supplementary file 2 — Peer Review File [file 41467_2024_48378_MOESM2_ESM.pdf]

nature portfolio

Peer Review File

The basal forebrain to lateral habenula circuitry mediates social behavioral maladaptation

Open Access This file is licensed under a Creative Commons Attribution 4.0 International License, which permits use, sharing, adaptation, distribution and reproduction in any medium or format, as long as you give appropriate credit to the original author(s) and the source, provide a link to the Creative Commons license, and indicate if changes were made. In the cases where the authors are anonymous, such as is the case for the reports of anonymous peer reviewers, author attribution should be to 'Anonymous Referee' followed by a clear attribution to the source work. The images or other third party material in this file are included in the article's Creative Commons license, unless indicated otherwise in a credit line to the material. If material is not included in the article's Creative Commons license and your intended use is not permitted by statutory regulation or exceeds the permitted use, you will need to obtain permission directly from the copyright holder. To view a copy of this license, visit http://creativecommons.org/licenses/by/4.0/.

REVIEWER COMMENTS

Reviewer #1 (Remarks to the Author):

The authors investigated circuit mechanisms of social fear responses with a focus on the potential role of the basal forebrain (BF) in mice. Using cell type-specific recording, they found that social fear conditioning increased the proportion of glutamatergic cells in the BF that were strongly activated during social interaction. Inhibiting the activity of these cells either by chemogenetics or optogenetics reduced the social avoidance of the conditioned mice. Moreover, the authors showed that the manipulation of BF glutamatergic cells projecting to the lateral habenula (LH) but not the ventral tegmental area (VTA) also weakened the social avoidance. This effect paralleled post- and pre-synaptic changes in BF glutamatergic-LH synapses. Based on these findings, the authors pinpoint glutamatergic projections from the BF to the LH as an essential circuit component that controls the expression of social fear response.

The manuscript contains substantial data from multiple levels of investigations that consistently support the authors' main argument. The experiments are well-designed and appear to be conducted carefully. The reported changes in various outcome measures are generally robust. Below, I list several suggestions to improve the clarity of the manuscript regarding the experimental design and data analysis.

1) The experimental schedule used in in vivo recording experiments is unclear. Were the unconditioned and conditioned groups two separate cohorts of mice? Or, was the recording conducted from a single cohort of mice before and after the social fear conditioning? Please clarify. Please also include a figure depicting the number of social approaches in each group during the recording session. I assume the number was lower for the conditioned group than the unconditioned group. If so, does the difference in the number of social approaches affect the likelihood of detecting cells with “Increase” and “Decrease” rates? I recommend that the authors confirm the results in the unconditioned group (Figures 1f and 3f) by down-sampling the number of social approaches to that in the conditioned mice.

2) The description of the fiber photometry experiment is very brief. It is not clear how the authors isolated a calcium-dependent fluorescence from a calcium-independent fluorescence. This point is essential to demonstrate that the large change in dF/F in LHb-projectors (Figure 5g, h) was not due to motion artifacts. Also, do the authors have any control data showing that the photometric recording from VTA-projectors was working?

3) During the three-chamber social interaction test, an unfamiliar stimulus mouse was placed in the acrylic cage in the social chamber. This induced a strong social avoidance response in the conditioned mice. However, I wonder why the authors used the new stimulus mouse rather than the original stimulus mouse used during the social fear conditioning. Please justify this chosen design.

4) To analyze the time spent in different components during the three-chamber social interaction test, the authors used two-way ANOVA (assuming it was two-way mixed ANOVA with the compartments as a within-subject factor and the group as a between-subject factor.) Please report the interaction and main effects in the results or figure legends (Fig. 2, 6, Extended data Fig, 1, 3, 4, 5, 6, 9, and 10.)

5) In the results of the optogenetic experiments, please specify the timing and temporal patterns of the photo-stimulation. Was it applied for the entire duration of the test? Also, please justify why the different temporal patterns were used for NpHR and GtACR1.

6) In Lines 419-420, the authors wrote “Fear behavior relies on complex cognitive processes that involve sensory perception, risk assessment, behavioral decision making and so on.” Can the authors find and cite review papers that support this view? It is a divisive point that fear behavior is supported by a top-down, goal-directed mechanism or bottom-up, habitual mechanism.

7) In Line 197, the authors wrote “Taken together, both chemogenetic and optogenetic studies demonstrate that BF vGluT2 neurons play a causal role in the regulation of social fear behavior…” Because the authors only conducted the loss-of-function experiment at this point, it is too strong to claim causality. I recommend toning it down to “…neurons are necessary for the regulation…”.

Reviewer #2 (Remarks to the Author):

In this manuscript titled: “The basal forebrain to lateral habenula circuitry mediates social behavioral maladaptation”, the authors investigate the role of basal forebrain glutamatergic neurons in social fear responses in mice. Using in vivo electrophysiology, chemogenetics, optogenetics, and behavioral monitoring, they identify basal forebrain glutamatergic neurons as critical for social fear behavior. The authors provide evidence that the circuit projections from the basal forebrain to the lateral habenula are specifically involved in the social fear responses using electrophysiology and optogenetic inhibition techniques, revealing a novel unexplored function for this circuit connection. Overall, this is an interesting study that has potential to provide insight and impact to the field, but some notable shortcomings should be addressed prior to consideration of publication.

First, the authors do a thorough job of narrowing down the basal forebrain population to glutamatergic neurons by excluding ChAT and vGAT positive neurons. Second, the combined use of contemporary

molecular genetic approaches (i.e. viral targeting, optogenetics, chemogenetics, electrophysiology in vivo and in slice, as well as in vivo calcium imaging) are appreciated. Also, there was a good effort to exclude projections to the VTA as candidate circuit. Regarding critiques, weaknesses or points to be addressed are itemized below:

• What about activation of this circuit? Although the loss-of-function approaches are valid, gain-of-function analysis should be incorporated (or at very least discussed/considered).

• Are there any collaterals that could have been missed that could be involved/responsible? What about projections to the periaqueductal gray? This region is known to mediate aspects of fear/social interaction.

• Also, related to the previous 2 points above- there was a significant oversight of references to other previously published works investigating basal forebrain projections/collaterals, or basal forebrain to habenular circuits.

• More details, evidence, and/or selective coordinates regarding basal forebrain targeting domains (subnuclei) should be included.

• Although the electrophysiological recording recordings in basal forebrain to show hyperactivity are appreciated, these data may be biased considering that only responsive neurons are monitored/detected. Showing alternate validation of cell type-specific responses would be helpful.

• Some data are not robustly sampled/described. For example, Figure 5 - is 6 bouts enough? How many bouts were recorded?

Reviewer #3 (Remarks to the Author):

In this study, the authors identified an important role of the glutamatergic BF-LHb projection in social fear expression after social fear conditioning. They demonstrated that the inhibiting BF glutamatergic cells but not GABAergic or cholinergic cells reduced social fear expression. Furthermore, the percentage of BF cells responding to social cues increases after social fear conditioning and the glutamatergic cells contribute to the increased response. The authors further demonstrated synaptic connection from BF to LHb and VTA using slice recording. Fiber photometry recording shows that BF to LHb projecting cells respond to social cues after social fear conditioning and nhibiting the BF-LHb connection is sufficient to reduce social fear. Lastly, the pathway from BF-LHb is strengthened after social fear conditioning. Overall, the study is interesting and used a wide array of circuit dissection tools. However, there are some important questions to be addressed to clarify the function of BF-LHb in fear expression.

Here are some specific comments:

1. SFC paradigm is a hybrid of classic fear conditioning (CS and US pairing) and typical social experience (e.g. defeat) induced social fear. Thus, it is difficult to determine whether the engaged circuit is socially relevant under more naturalistic conditions or whether the circuit is also involved in non-social fear learning. To address these questions, some additional experiments are needed. First, if the BF Vglut2 cells are inhibited, will defeat-induced social avoidance or CS-US fear conditioning-induced fear response, e.g. freezing, during CS presentation be reduced? Second, will BF vglut2 cells increase responses to social cues after defeat or CS (e.g. replace the mouse with an object with odor) after US-CS pairing?

2. It is difficult to conclude whether BF cells projecting to VTA and LHb are indeed distinct based on the dual-color retrograde virus strategy because it is hard to tell the labeling efficiency of the virus. If only a small fraction of Vglut2 cells are infected and only a small percentage of VTA positive axons take up rabies virus, the chance for the same cell being labeled with retrograde viruses from both VTA and LHb will be low. The authors should count the percentage of BF Vglut2 cells that are retrogradely labeled from each region and the predict the level of overlap if just by chance, and compare it with the observed results to better understand whether the number of double labeled cells is high or low.

3. Rabies is quite toxic. Please perform cell count in the recording side and the contralateral side to confirm no obvious cell loss.

4. Fiber photometry is well suited for longitudinal recording. It will be interesting to see that the response of BF-LHb Vglut2 cells are low during social interaction before fear conditioning and increase afterwards. The social vs. neutral comparison does not address whether the response to social cue emerges after social fear conditioning.

5. Please show individual animal/cell data for all plots.

Reviewer #1:

The authors investigated circuit mechanisms of social fear responses with a focus on the potential role of the basal forebrain (BF) in mice. Using cell type-specific recording, they found that social fear conditioning increased the proportion of glutamatergic cells in the BF that were strongly activated during social interaction. Inhibiting the activity of these cells either by chemogenetics or optogenetics reduced the social avoidance of the conditioned mice. Moreover, the authors showed that the manipulation of BF glutamatergic cells projecting to the lateral habenula (LH) but not the ventral tegmental area (VTA) also weakened the social avoidance. This effect paralleled post- and pre-synaptic changes in BF glutamatergic-LH synapses. Based on these findings, the authors pinpoint glutamatergic projections from the BF to the LH as an essential circuit component that controls the expression of social fear response.

The manuscript contains substantial data from multiple levels of investigations that consistently support the authors' main argument. The experiments are well-designed and appear to be conducted carefully. The reported changes in various outcome measures are generally robust. Below, I list several suggestions to improve the clarity of the manuscript regarding the experimental design and data analysis.

(Response) We sincerely appreciate the positive and helpful evaluation on our study and constructive suggestions from the Reviewer, which are very helpful

in significantly improving our study. We have performed multiple sets of new experiments and data analyses to address the Reviewer's comments and substantially revised our manuscript. A detailed point-by-point response to the Reviewer's comments is presented below.

1. *The experimental schedule used in in vivo recording experiments is unclear. Were the unconditioned and conditioned groups two separate cohorts of mice? Or, was the recording conducted from a single cohort of mice before and after the social fear conditioning? Please clarify. Please also include a figure depicting the number of social approaches in each group during the recording session. I assume the number was lower for the conditioned group than the unconditioned group. If so, does the difference in the number of social approaches affect the likelihood of detecting cells with "Increase" and "Decrease" rates? I recommend that the authors confirm the results in the unconditioned group (Figures 1f and 3f) by down-sampling the number of social approaches to that in the conditioned mice.*

(Response) Thank you for raising this question regarding experimental schedule for *in vivo* recording experiments. We performed multichannel electrophysiological recordings in two separate cohorts of mice (unconditioned and conditioned groups). As suggested, we have provided more information to clarify our experimental procedures in the revised manuscript (lines 103-106, 256-257).

To clarify the difference in the number of social approaches between unconditioned and conditioned mice, we analyzed the number of social approaches in each group during the recording session, and found that conditioned mice approached less than unconditioned mice (new Fig. 1c and 3c). As the Review suggested, we down-sampled the number of social approaches in unconditioned mice by examining spiking activity of the first six interaction bouts for all recordings, and then classified cells with “Increase”, “Decrease” or “No change”. As a result, we confirmed our prior findings and showed that the fraction of “Increase” cells was significantly higher in conditioned mice as compared to unconditioned controls. These new analyzed results are now presented in the revised manuscript (new Fig. 1 and 3).

2. *The description of the fiber photometry experiment is very brief. It is not clear how the authors isolated a calcium-dependent fluorescence from a calcium-independent fluorescence. This point is essential to demonstrate that the large change in dF/F in LHb-projectors (Figure 5g, h) was not due to motion artifacts. Also, do the authors have any control data showing that the photometric recording from VTA-projectors was working?*

(Response) We thank the Reviewer for raising the technique issue. As suggested, we have provided detailed information to describe fiber photometry experiment in the revised manuscript (lines 809-817).

By taking advantage of genetically-encoded Ca^{2+} indicators such as

GCaMP proteins, fiber photometry is widely used to monitor the activity of genetically-defined neuron populations in freely-behaving animals[1]. To exclude the potential influence of motion artifacts, we added a control experiment by expressing enhanced yellow fluorescent protein (EYFP) in the BF vGluT2 neurons and performed fiber photometry during social fear behavioral expression. We found that EYFP mice did not show any clear fluorescence change following both neutral and social investigations (new Extended Data Fig. 2q-t), indicating that the signals in BF vGluT2 GCaMP6-expressing mice were Ca^{2+} in nature but not motion artifacts. Indeed, this control experiment was routinely used by to exclude the potential influence of motion artifacts[2-4].

For each photometric recording, we verified the effectiveness of our recordings by checking spontaneous calcium events[5]. Only those data with spontaneous calcium events during the first 10 min habituation session were included for further analysis. Besides, post-mortem examination was also conducted in all mice, and only those with virus expression under optical fiber as well as correct placement of the optical fiber were included. For clarity, we incorporated spontaneous calcium event analysis result for both LHb- and VTA-projecting BF vGluT2 neurons in the revised manuscript (new Fig. 5g and j).

3. *During the three-chamber social interaction test, an unfamiliar stimulus mouse was placed in the acrylic cage in the social chamber. This induced a*

strong social avoidance response in the conditioned mice. However, I wonder why the authors used the new stimulus mouse rather than the original stimulus mouse used during the social fear conditioning. Please justify this chosen design.

(Response) Thank the Reviewer for pointing out this concern. In clinical condition, patients with social phobia exhibit avoidance to general social situations rather than to a specific social subject[6, 7]. In this study, we therefore used an unfamiliar stimulus mouse rather than the original stimulus mouse used during the social fear conditioning. Indeed, this experimental design was widely used by different groups including ours in previous studies[8-10]. The justification for this experimental design is added in the revised manuscript (lines 93-96).

4. *To analyze the time spent in different components during the three-chamber social interaction test, the authors used two-way ANOVA (assuming it was two-way mixed ANOVA with the compartments as a within-subject factor and the group as a between-subject factor.) Please report the interaction and main effects in the results or figure legends (Fig. 2, 6, Extended data Fig, 1, 3, 4, 5, 6, 9, and 10.)*

(Response) Thank the Reviewer for the nice suggestion. We now provided both interaction and main effects for each two-way ANOVA analysis in the figure legends in the revised manuscript (Fig. 2e, 2g, 6d, 6f, and new Extended

Data Fig.1c, 1e, 4d, 4f, 5d, 5f, 6d, 6f, 7d, 7h, 8f, 9f, 9h, 13d, 13h, 14d, 14f, 15d, 15f).

5. *In the results of the optogenetic experiments, please specify the timing and temporal patterns of the photo-stimulation. Was it applied for the entire duration of the test? Also, please justify why the different temporal patterns were used for NpHR and GtACR1.*

(Response) Thank the Reviewer for his/her concern regarding the technique issue. An elegant study by Yizhar's group demonstrated that activation of light-gated chloride channels (GtACR1) allows robust inhibition of action potential firing in cultured hippocampal neurons; however, illumination of GtACR1-expressing presynaptic terminals evokes EPSCs and triggers neurotransmitter release upon light onset, potentially limiting the utility of these tools for temporally precise inhibition of presynaptic terminals[11]. Fortunately, their study suggested that eNpHR3.0 is the most suitable existing tool for synaptic terminal silencing[11]. Therefore, we used GtACR1 to inhibit BF vGluT2 cell bodies and eNpHR3.0 for axon terminal inhibition in this study.

Several reasons were taken into account in choosing different temporal patterns of the photo-stimulation for eNpHR3.0 and GtACR1. (1) The action spectrum of NpHR (near 580 nm) allows more safety for constant light illumination due to limited thermal effect to the brain tissue[12, 13], whereas blue light for activating GtACR1 may cause higher thermal effect if it delivered

constantly. (2) Faster kinetics and higher light sensitivity allow GtACR1 as a robust and long-term optogenetic inhibition tool via brief pulses of blue light[14-16]. (3) Among literatures, many *in vivo* long-term inhibition studies used constant yellow light for NpHR[17-19] and blue light pulses for GtACR1[20, 21].

We have provided detailed information to specify the timing and temporal patterns of the photo-stimulation in the revised manuscript (lines 214-215, 441-442, 786-787).

6. *In Lines 419-420, the authors wrote "Fear behavior relies on complex cognitive processes that involve sensory perception, risk assessment, behavioral decision making and so on." Can the authors find and cite review papers that support this view? It is a divisive point that fear behavior is supported by a top-down, goal-directed mechanism or bottom-up, habitual mechanism.*

(Response) As the Reviewer suggested, we have cited review papers in the revised manuscript (line 504). Although early theory emphasized a unitary response mechanism for fear, recent evidence suggests a complex neural network that involves multiple, independent circuits contributes to process different types of fear[22, 23]. For instance, a two-system framework was suggested by Prof. Joseph E. LeDoux that behavioral responses and conscious feeling states elicited by threats may engage distinct neural circuits[24]. In this perspective, the threat-elicited social responses discussed in

our study relies on multiple cognitive circuits including sensory system, defensive survival circuits, and defensive action circuits[22, 24]. Each of these processes may involve complicate mechanisms, such as top-down goal-directed mechanism and bottom-up habitual mechanism. For example, the prefrontal cortex (PFC) mediates top-down control to improve goal-directed behavior through attention, decision-making and higher cognitive integration[25]. At the same time, bottom-up processing contributes critically to facilitate threat cue sensory perception[26].

7. *In Line 197, the authors wrote "Taken together, both chemogenetic and optogenetic studies demonstrate that BF vGluT2 neurons play a causal role in the regulation of social fear behavior…" Because the authors only conducted the loss-of-function experiment at this point, it is too strong to claim causality. I recommend toning it down to "…neurons are necessary for the regulation…".*

(Response) As the Reviewer recommended, we changed the sentence to "BF vGluT2 neurons are necessary for the regulation of social fear behavior" (lines 234-236).

Reviewer #2:

In this manuscript titled: “The basal forebrain to lateral habenula circuitry mediates social behavioral maladaptation”, the authors investigate the role of basal forebrain glutamatergic neurons in social fear responses in mice. Using in vivo electrophysiology, chemogenetics, optogenetics, and behavioral monitoring, they identify basal forebrain glutamatergic neurons as critical for social fear behavior. The authors provide evidence that the circuit projections from the basal forebrain to the lateral habenula are specifically involved in the social fear responses using electrophysiology and optogenetic inhibition techniques, revealing a novel unexplored function for this circuit connection. Overall, this is an interesting study that has potential to provide insight and impact to the field, but some notable shortcomings should be addressed prior to consideration of publication.

First, the authors do a thorough job of narrowing down the basal forebrain population to glutamatergic neurons by excluding ChAT and vGAT positive neurons. Second, the combined use of contemporary molecular genetic approaches (i.e. viral targeting, optogenetics, chemogenetics, electrophysiology in vivo and in slice, as well as in vivo calcium imaging) are appreciated. Also, there was a good effort to exclude projections to the VTA as candidate circuit. Regarding critiques, weaknesses or points to be addressed are itemized below:

(Response) Thank the Reviewer for his/her enthusiasm and insightful comments on our manuscript. We have conducted a series of new experiments and data analyses to carefully address these questions. As presented in our revised manuscript, our new results have significantly strengthened the study. A detailed point-by-point response to the Reviewer's comments is presented below.

1. *What about activation of this circuit? Although the loss-of-function approaches are valid, gain-of-function analysis should be incorporated (or at very least discussed/considered).*

(Response) As the Reviewer suggested, we conducted gain-of-function analysis of the BF to LHb glutamatergic circuit. We found that in mice experienced SFC, optogenetic activation of this circuit did not exacerbate animals' fear behavioral responses (new Extended Data Fig. 15). This observation is likely due to a ceiling effect. In other words, the social fear conditioning paradigm already heavily potentiated the BF-LHb projection (please see original Fig. 7), which occluded further activation of this projection with optogenetics. The new data have been incorporated in the revised manuscript (new Extended Data Fig. 15), and we discussed accordingly in the main text (lines 436-451).

2. *Are there any collaterals that could have been missed that could be involved/responsible? What about projections to the periaqueductal gray? This region is known to mediate aspects of fear/social interaction.*

(Response) We appreciated the Reviewer for raising the collaterals issue and paid special attention to the periaqueductal gray (PAG) given its known function in mediating social fear [8] and other types of fear responses[22, 27]. We conducted brain slice recording to examine the glutamatergic connection between BF and PAG, and compared its strength and probability with BF-LHb and BF-VTA glutamatergic connections. We found that the amplitude of evoked EPSCs was much smaller in BF-PAG connection than those of BF-LHb connection or BF-VTA connection (PAG, 40.23 ± 3.01 pA; LHb, 257.2 ± 61.53 pA; VTA, 79.93 ± 14.05 pA). Moreover, the connection probability was much lower in BF-PAG connection than those of BF-LHb connection or BF-VTA connection (PAG, 18.75%, 3 out of 16; LHb, 78.26%, 18 out of 23; VTA, 63.16%, 12 out of 19). These new set of data is well consistent with a prior report that showed sparse axon terminals of BF vGluT2 neurons in the PAG region[28]. Together, these evidences indicate a weak connection between BF vGluT2 neurons and the PAG. We have discussed this issue in the revised manuscript (lines 639-640).

3. *Also, related to the previous 2 points above- there was a significant oversight of references to other previously published works investigating basal forebrain projections/collaterals, or basal forebrain to habenular circuits.*

(Response) Thank the Reviewer for making this valuable point. The BF contains three major genetically defined cell types: cholinergic neurons, glutamatergic neurons and GABAergic neurons[29, 30]. Although previous studies mainly focused on the function of BF cholinergic neurons, recent studies started to unravel the role of glutamatergic neurons and GABAergic neurons. For example, it is found that BF glutamatergic neurons were activated by various threatening stimuli and participated in mediating of wakefulness[31] and anorexia-like phenotypes[32] via their projections to the VTA. In addition, an excitatory projection from the BF to the lateral hypothalamus is activated by food-odor related stimuli, and potently drives hypophagia[33]. As for BF GABAergic neurons, it is found that GABAergic projections from the BF to the LHb modulate aggression reward[34]. Besides, current studies revealed projections from different subregions of the BF (see also response to your Comment 4) to the LHb contribute to emotion relevant responses[35-37].

We extended our discussion on the basal forebrain projections/collaterals and cited references accordingly (lines 624-627, 637-645).

4. *More details, evidence, and/or selective coordinates regarding basal forebrain targeting domains (subnuclei) should be included.*

(Response) Thank the Reviewer for the constructive suggestion. Traditionally, the BF contains subregions including the medial septum (MS), vertical and horizontal limbs of the diagonal band (VDB and HDB), and back to the magnocellular preoptic nucleus (MCPO) and substantia innominata (SI). However, there is no consensus on the definition of the BF as it is anatomically complex and lacks clear boundaries. For instance, some studies suggest that the SI overlaps with the nucleus basalis, the ventral pallidum (VP), extended amygdala, and other structures in this region[38], which may also be included in the BF. In this study, we targeted the BF subregions including the HDB, MCPO and part of SI, which were previously linked to multiple functions with cell-type specificity including sleep-wake control[29], high-fat food intake[39] and social behavior[40].

Existing literature reveals distinct functions of BF subregions. For example, the SI encodes aversive information and bi-directionally modulates negative reinforcement learning[41]. In comparison, it was found that the SI is activated by aversive stimuli and inhibited by reward stimuli, which is involved in modulating depressive-like behaviors[37]. Moreover, the MS was found to mediate aversion induced by both auditory and somatosensory stimuli[35], and can transmit innately aversive signals via a bottom-up multimodal sensory pathway, allowing animals to efficiently avoid unfavorable environments[36]. Except for these emotional functions, a recent study demonstrated that vGluT2 neurons

mainly in the VDB are important for anorexia-like phenotypes elicited by external threat[32].

Apart from well-studied functions of the BF cholinergic neurons, including arousal, attention, sleep-wake circle, learning and memory[29, 42-44], there is an accumulating literature that starts to uncover non-canonical functions of the BF, especially in emotion process. As suggested, we have expanded our discussion on this valuable point in the revised manuscript (lines 510-529).

5. *Although the electrophysiological recording recordings in basal forebrain to show hyperactivity are appreciated, these data may be biased considering that only responsive neurons are monitored/detected. Showing alternate validation of cell type-specific responses would be helpful.*

(Response) We appreciated the Reviewer's suggestion, and carried out new experiments to address his/her concern. We selectively expressed GCaMP in BF vGluT2, ChAT and vGAT neurons and monitored the activity of these neuronal types during a social fear test. We found that vGluT2 neurons exhibited a robust activation during social fear expression compared to neutral cage investigation (new Extended Data Fig. 2e-h). In comparison, ChAT neurons exhibited only slight responses to both social and neutral interactions (new Extended Data Fig. 2i-l). Similarly, vGAT neurons exhibited mild responses to both social and neutral interactions, and the fluorescence signals were similar between social and neutral interactions (new Extended Data Fig.

2m-p). These findings are consistent with our original results that showed chemogenetic inhibition of BF vGluT2, but not ChAT or vGAT neurons, attenuates social fear expression (please see original Fig. 2 and new Extended Data Fig. 4 and 5). Together, these new data complement our electrophysiological recordings and confirm a critical role of BF vGluT2 neurons in regulation of social fear behavior. We have included these new data in the revised manuscript (lines 136-150).

6. *Some data are not robustly sampled/described. For example, Figure 5 - is 6 bouts enough? How many bouts were recorded?*

(Response) Thank the Reviewer for pointing out the sampling size issue. Indeed, following the social fear conditioning, the number of social approaches in conditioned mice was largely reduced during the three-chamber social interaction task (Please see Extended Data Fig. 1g). Therefore, for our fiber photometry experiments, the analyzed number of bouts varied between 5 and 10 for each mouse. For presentation, we illustrated a representative data with 8 bouts in the revised manuscript (new Fig. 5k, n).

Reviewer #3:

In this study, the authors identified an important role of the glutamatergic BF-LHb projection in social fear expression after social fear conditioning. They demonstrated that the inhibiting BF glutamatergic cells but not GABAergic or cholinergic cells reduced social fear expression. Furthermore, the percentage of BF cells responding to social cues increases after social fear conditioning and the glutamatergic cells contribute to the increased response. The authors further demonstrated synaptic connection from BF to LHb and VTA using slice recording. Fiber photometry recording shows that BF to LHb projecting cells respond to social cues after social fear conditioning and inhibiting the BF-LHb connection is sufficient to reduce social fear. Lastly, the pathway from BF-LHb is strengthened after social fear conditioning. Overall, the study is interesting and used a wide array of circuit dissection tools. However, there are some important questions to be addressed to clarify the function of BF-LHb in fear expression.

(Response) We would like to thank the Reviewer for his/her enthusiasm and insightful comments on our manuscript. We have conducted a series of new experiments and data analyses to carefully address these questions. We believe that these new results have significantly strengthened our study. A detailed point-by-point response to the Reviewer's comments is presented below.

1. *SFC paradigm is a hybrid of classic fear conditioning (CS and US pairing) and typical social experience (e.g. defeat) induced social fear. Thus, it is difficult to determine whether the engaged circuit is socially relevant under more naturalistic conditions or whether the circuit is also involved in non-social fear learning. To address these questions, some additional experiments are needed. First, if the BF Vglut2 cells are inhibited, will defeat-induced social avoidance or CS-US fear conditioning-induced fear response, e.g. freezing, during CS presentation be reduced? Second, will BF vglut2 cells increase responses to social cues after defeat or CS (e.g. replace the mouse with an object with odor) after US-CS pairing?*

(Response) We are glad that the Reviewer raised the specificity issue. As recommended, we first employed another paradigm, namely a three-day sub-chronic social defeat by an aggressive CD1 mouse, to induce social fear[8], and performed both fiber photometry recording and chemogenetic inhibition. We found that BF vGluT2 neurons were robustly activated during social interaction with a CD1 stimulus mouse as compared to neutral cage interaction (new Extended Data Fig. 8a-d). Moreover, chemogenetic inhibition of BF vGluT2 neurons largely reduced social fear expression in a social approach-avoidance test (new Extended Data Fig. 8e-h). Together, these findings indicate that BF vGluT2 neurons indeed respond and contribute to social fear behavior under more naturalistic conditions.

Second, we established a classical US-CS conditioning by pairing chocolate odor with foot shock. Similarly, we found that BF vGluT2 neurons were activated by chocolate odor after conditioning (new Extended Data Fig. 9a-d). Furthermore, chemogenetic inhibition of BF vGluT2 neurons reduced fear and avoidance behavior elicited by chocolate odor (new Extended Data Fig. 9e-k). These data indicate that BF vGluT2 neurons have a general role in both socially relevant fear behaviors and non-social fear learning.

These new results and relevant discussions have been incorporated in the revised manuscript (lines 237-248, 556-560).

2. *It is difficult to conclude whether BF cells projecting to VTA and LHb are indeed distinct based on the dual-color retrograde virus strategy because it is hard to tell the labeling efficiency of the virus. If only a small fraction of Vglut2 cells are infected and only a small percentage of VTA positive axons take up rabies virus, the chance for the same cell being labeled with retrograde viruses from both VTA and LHb will be low. The authors should count the percentage of BF Vglut2 cells that are retrogradely labeled from each region and the predict the level of overlap if just by chance, and compare it with the observed results to better understand whether the number of double labeled cells is high or low.*

(Response) Thank the Reviewer for raising this important issue. To address this concern, we carefully repeated this retrograde labeling experiment in

another cohort of mice. Similarly, a relatively low overlap rate (5.65%) was found between VTA- and LHb-projecting BF vGluT2 neurons. We now pooled these two batches of data together and made a new Fig. 5c. Even so, we totally agree with the Reviewer's comment that labeling efficiency of the virus is an important factor that could affect the count of overlap. For the sake of rigor, we therefore removed our original description that BF cells projecting to VTA and LHb are distinct in the revised manuscript. Future studies using tracing virus with high infection efficiency are needed to eventually resolve this issue.

3. *Rabies is quite toxic. Please perform cell count in the recording side and the contralateral side to confirm no obvious cell loss.*

(Response) We agree with the Reviewer that Rabies virus is toxic, which may limit its application in high efficient tracing and functional recording/manipulation. For this very reason, we used a newly developed virus based on rabies virus CVS-N2c strain in this study, which has reduced cytotoxicity and enhanced efficiency[45]. The same virus was also used for two-photo calcium imaging and fiber photometry recently[46, 47]. To follow the Reviewer's instruction, we performed NeuN staining and compared the number of neurons between the recording side and the contralateral side. It turned out that there is no significant difference in cell numbers between two sides (new Extended Data Fig. 10c, d, g, h), suggesting no obvious cell loss in

our experimental conditions. These new results and discussions have been incorporated in the revised manuscript (new Extended Data Fig. 10c, d, g, h).

4. *Fiber photometry is well suited for longitudinal recording. It will be interesting to see that the response of BF-LHb Vglut2 cells are low during social interaction before fear conditioning and increase afterwards. The social vs. neutral comparison does not address whether the response to social cue emerges after social fear conditioning.*

(Response) We appreciate this insightful instruction. As suggested, we have performed fiber photometry experiments using RV-GCaMP to monitor projection specific activity of BF vGluT2 neurons, and compared the fluorescent signals before and after the SFC. We observed a slight activation of LHb-projection BF vGluT2 neurons before the SFC. In contrast, an obviously larger activation was detected after the SFC (new Fig. 5). These data demonstrated that the response to social cue emerges after social fear conditioning. At the meantime, we did not detect a prominent activity of VTA-projection BF vGluT2 neurons during social interaction either before or after the SFC, suggesting a projection specific activity of BF vGluT2 neurons in social fear. These new results are now presented in the revised manuscript (new Fig. 5).

5. *Please show individual animal/cell data for all plots.*

(Response) We thank the Reviewer for the valuable suggestion. As recommended, we now showed individual animal/cell data in our new plots. For in vivo electrophysiology data in Fig. 1 and Fig. 3, we plotted the figures in violin style with quartiles because these two figures involve too many data points.

References:

1. Li, Y., Liu, Z.X., Guo, Q.C. & Luo, M.M. Long-term Fiber Photometry for Neuroscience Studies. *Neurosci Bull* **35**, 425-433 (2019).

2. Li, Y.*, et al.* Serotonin neurons in the dorsal raphe nucleus encode reward signals. *Nat Commun* **7** (2016).

3. Huang, L.*, et al.* A retinoraphe projection regulates serotonergic activity and looming-evoked defensive behaviour. *Nat Commun* **8** (2017).

4. Huang, L.*, et al.* A Visual Circuit Related to Habenula Underlies the Antidepressive Effects of Light Therapy. *Neuron* **102**, 128-+ (2019).

5. Bavley, C.C.*, et al.* Cocaine- and stress-primed reinstatement of drug-associated memories elicit differential behavioral and frontostriatal circuit activity patterns via recruitment of L-type Ca²⁺channels. *Mol Psychiatr* **25**, 2373-2391 (2020).

6. denBoer, J.A. Social phobia: epidemiology, recognition, and treatment. *Brit*

Med J **315**, 796-800 (1997).

7. Ruipérez, M.A., García-Palacios, A. & Botella, C. Clinical features and treatment response in social phobia:: axis II comorbidity and social phobia subtypes. *Psicothema* **14**, 426-433 (2002).

8. Franklin, T.B.*, et al.* Prefrontal cortical control of a brainstem social behavior circuit. *Nat Neurosci* **20**, 260-270 (2017).

9. Xu, H.F.*, et al.* A Disinhibitory Microcircuit Mediates Conditioned Social Fear in the Prefrontal Cortex. *Neuron* **102**, 668-+ (2019).

10. Toth, I., Neumann, I.D. & Slattery, D.A. Social Fear Conditioning: A Novel and Specific Animal Model to Study Social Anxiety Disorder. *Neuropsychopharmacol* **37**, 1433-1443 (2012).

11. Mahn, M., Prigge, M., Ron, S., Levy, R. & Yizhar, O. Biophysical constraints of optogenetic inhibition at presynaptic terminals. *Nat Neurosci* **19**, 554-+ (2016).

12. Fenno, L., Yizhar, O. & Deisseroth, K. The Development and Application of Optogenetics. *Annu Rev Neurosci* **34**, 389-412 (2011).

13. Yizhar, O., Fenno, L.E., Davidson, T.J., Mogri, M. & Deisseroth, K. Optogenetics in Neural Systems. *Neuron* **71**, 9-34 (2011).

14. Govorunova, E.G., Sineshchekov, O.A., Janz, R., Liu, X.Q. & Spudich, J.L. Natural light-gated anion channels: A family of microbial rhodopsins for advanced optogenetics. *Science* **349**, 647-650 (2015).

15. Li, N.*, et al.* Spatiotemporal constraints on optogenetic inactivation in

cortical circuits. *Elife* **8** (2019).

16. Huang, S., *et al.* Optogenetic control of the guard cell membrane potential and stomatal movement by the light-gated anion channel GtACR1. *Sci Adv* **7** (2021).

17. Gunaydin, L.A., *et al.* Natural Neural Projection Dynamics Underlying Social Behavior. *Cell* **157**, 1535-1551 (2014).

18. Siciliano, C.A., *et al.* A cortical-brainstem circuit predicts and governs compulsive alcohol drinking. *Science* **366**, 1008-+ (2019).

19. Dong, Y., *et al.* Stress relief as a natural resilience mechanism against depression-like behaviors. *Neuron* **111**, 3789-3801 e3786 (2023).

20. Sun, L., *et al.* Parabrachial nucleus circuit governs neuropathic pain-like behavior. *Nat Commun* **11** (2020).

21. Xie, Z.Y., *et al.* Mechanically evoked defensive attack is controlled by GABAergic neurons in the anterior hypothalamic nucleus. *Nat Neurosci* **25**, 72-+ (2022).

22. Gross, C.T. & Canteras, N.S. The many paths to fear. *Nat Rev Neurosci* **13**, 651-658 (2012).

23. Adolphs, R. The Biology of Fear. *Curr Biol* **23**, R79-R93 (2013).

24. Joseph E. LeDoux, Ph.D. , & Daniel S. Pine, M.D. Using Neuroscience to Help Understand Fear and Anxiety: A Two-System Framework. **173**, 1083-1093 (2016).

25. Marek, R., Sun, Y.J. & Sah, P. Neural circuits for a top-down control of fear

and extinction. *Psychopharmacology* **236**, 313-320 (2019).

26. Sussman, T.J., Jin, J. & Mohanty, A. Top-down and bottom-up factors in threat-related perception and attention in anxiety. *Biological Psychology* **121**, 160-172 (2016).

27. Tovote, P.*, et al.* Midbrain circuits for defensive behaviour. *Nature* **534**, 206-212 (2016).

28. Do, J.P.*, et al.* Cell type-specific long-range connections of basal forebrain circuit. *Elife* **5** (2016).

29. Xu, M.*, et al.* Basal forebrain circuit for sleep-wake control. *Nat Neurosci* **18**, 1641-1647 (2015).

30. Do, J.P.*, et al.* Cell type-specific long-range connections of basal forebrain circuit. *Elife* **5** (2016).

31. Cai, P.*, et al.* A glutamatergic basal forebrain to midbrain circuit mediates wakefulness and defensive behavior. *Neuropharmacology* **208** (2022).

32. Cai, J.*, et al.* An excitatory projection from the basal forebrain to the ventral tegmental area that underlies anorexia-like phenotypes. *Neuron* (2023).

33. Patel, J.M.*, et al.* Sensory perception drives food avoidance through excitatory basal forebrain circuits. *Elife* **8** (2019).

34. Golden, S.A.*, et al.* Basal forebrain projections to the lateral habenula modulate aggression reward. *Nature* **534**, 688-+ (2016).

35. Zhang, G.W.*, et al.* Transforming Sensory Cues into Aversive Emotion via Septal-Habenular Pathway. *Neuron* **99**, 1016-+ (2018).

36. Shen, L., *et al.* A bottom-up reward pathway mediated by somatostatin neurons in the medial septum complex underlying appetitive learning. *Nat Commun* **13** (2022).

37. Cui, Y.T., *et al.* Reward ameliorates depressive-like behaviors via inhibition of the substantia innominata to the lateral habenula projection. *Science Advances* **8** (2022).

38. Agostinelli, L.J., Geerling, J.C. & Scammell, T.E. Basal forebrain subcortical projections. *Brain Structure and Function* **224**, 1097-1117 (2019).

39. Zhu, C., *et al.* Somatostatin Neurons in the Basal Forebrain Promote High-Calorie Food Intake. *Cell Reports* **20**, 112-123 (2017).

40. Wang, J., *et al.* Basal forebrain mediates prosocial behavior via disinhibition of midbrain dopamine neurons. *Proceedings of the National Academy of Sciences* **118**, e2019295118 (2021).

41. Cui, Y., *et al.* A Central Amygdala-Substantia Innominata Neural Circuitry Encodes Aversive Reinforcement Signals. *Cell Reports* **21**, 1770-1782 (2017).

42. Jones, B.E. Modulation of cortical activation and behavioral arousal by cholinergic and orexinergic systems. *Annals of the New York Academy of Sciences* **1129**, 26-34 (2008).

43. Ballinger, E.C., Ananth, M., Talmage, D.A. & Role, L.W. Basal Forebrain Cholinergic Circuits and Signaling in Cognition and Cognitive Decline. *Neuron* **91**, 1199-1218 (2016).

44. Thiele, A. & Bellgrove, M.A. Neuromodulation of Attention. *Neuron* **97**,

769-785 (2018).

45. Lin, K., *et al.* Improved production and expanded application of CVS-N2c-ΔG virus for retrograde tracing. *bioRxiv* (2022).

46. Gu, M.Q., *et al.* Rabies virus-based labeling of layer 6 corticothalamic neurons for two-photon imaging. *Iscience* **26** (2023).

47. Chen, A.-X., *et al.* Specific Hypothalamic Neurons Required for Sensing Conspecific Male Cues Relevant to Inter-male Aggression. *Neuron* **108**, 763-774.e766 (2020).

REVIEWERS' COMMENTS

Reviewer #1 (Remarks to the Author):

The authors addressed all my comments. I do not have any further concerns about the manuscript. However, in future fiber photometry experiments, I suggest the authors consider simultaneously monitoring calcium-dependent and calcium-independent signals in each mouse. This two-channel approach isolates calcium activity more effectively from potential motion-related artifact. See the following papers for more details.

Hormigo et al., eLife, 2023, PMID: 38048270

Matias et al., eLife, 2017, PMID: 28322190

Reviewer #2 (Remarks to the Author):

Overall, I think the authors nicely address my raised concerns, as well as those raised by others. The addition of extensive new experimental data were appreciated, and thoughtful attention was paid to suggested modifications. I endorse publication.

Reviewer #3 (Remarks to the Author):

The authors have thoroughly addressed all the reviewer's comment. The new results have significantly strengthened the manuscript. The reviewer now supports its publication in Nature Communication.
